# Repurposing Drug Metabolites into Dual β‑Adrenergic Receptor–Carbonic Anhydrase Modulators as Potential Tools for Ocular Disorders

**DOI:** 10.1021/acs.jmedchem.5c01459

**Published:** 2025-08-26

**Authors:** Andrea Ammara, Alessandra Carone, Laura Lucarini, Silvia Sgambellone, Silvia Marri, Serafina Villano, Rosanna Matucci, Gerta Luga, Chiara Fittipaldi, Riccardo Pecori, Giuseppe Pieraccini, Claudia Di Serio, Andrea García-Llorca, Thor Eysteinsson, Stanislav Kalinin, Julius Aleksi Olavi Viita, Arto Urtti, Fabrizio Carta, Silvia Selleri, Claudiu T. Supuran

**Affiliations:** † NEUROFARBA Department, Sezione di Scienze Farmaceutiche e Nutraceutiche, 9300 University of Florence, Via Ugo Schiff 6, Sesto Fiorentino, Florence 50019, Italy; ‡ Department of Neuroscience, Psychology, Drug Research and Child Health (NEUROFARBA), Section of Pharmacology, 9300 University of Florence, Viale Gaetano Pieraccini, 6, Florence 50139, Italy; § 9151 German Cancer Research Center (DKFZ), Im Neuenheimer Feld 280, Heidelberg 69120, Germany; ∥ Department of Health Sciences, CISM Mass Spectrometry Centre, 9300 University of Florence, Viale Gaetano Pieraccini 6, Florence 50139, Italy; ⊥ Experimental and Clinical Medicine Department, Geriatric Intensive Care Unit, 9300 University of Florence, Azienda Ospedaliera Universitaria Careggi, Viale Gaetano Pieraccini 6, Florence 50139, Italy; # Department of Physiology, Biomedical Center, Faculty of Medicine, University of Iceland, Reykjavík 101, Iceland; ∇ School of Pharmacy, Faculty of Health Sciences, 163043 University of Eastern Finland, Yliopistonrinne 3, Kuopio 70211, Finland

## Abstract

We report the regioselective chemical derivatization of (*R*)-2-((4-aminophenethyl)­amino)-1-phenylethan-1-ol, the primary metabolite of the β_3_-Adrenergic Receptor (β_3_-AR) agonist mirabegron, with prototypical Carbonic Anhydrase Inhibitors (CAIs) to afford the carbamates **10–14** and the ureido derivatives **15–18**. Such compounds were endowed *in vitro* with distinct inhibition profiles for the human (h) Carbonic Anhydrases (CAs) and showed preferential agonisms for the β_3_-AR subtype. Among them, **14** induced remarkable intraocular pressure (IOP) lowering in an *in vivo* transient model of ocular hypertension, with the maximal effect at 120 min post-administration at 1% w/v concentration. Furthermore, the high stability of the compounds in rabbit plasma and their ability to induce full vasodilation in isolated porcine retinal arteries suggested that the observed *in vivo* effects likely result from a combination of conventional aqueous humor reduction and modulation of ocular vascular tone, both of which are mediated by CAs and β-ARs. The pronounced melanosomal accumulation of representative compounds **14** and **16** highlights their potential as ideal candidates for evaluating pharmacokinetic profiles in ophthalmic applications. The results of this study provide strong evidence for the biomedical repurposing of a neglected metabolite through a novel class of dual-targeting ligands, also offering a promising strategy to help counteract the ongoing decline in drug discovery.

## Graphical abstract

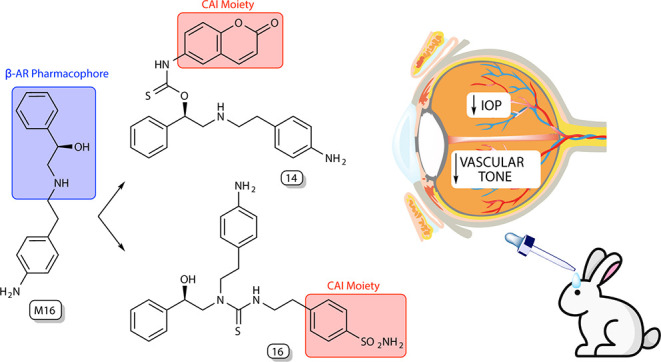

## Introduction

The constant need of finding new and effective drugs for the management of diseases affecting humans is only partially to ascribe to drug resistance phenomena as result of adaptive evolutionary patterns triggered from exposure to medications.^1–7^ A deep focus on the actual panorama of drug Research and Development (R&D) on a global scale brings to light that there is a remarkable crisis either in productivity or efficiency.^8,9^ These outputs indicate the number of new drugs brought to market per billion US dollars allocated to R&D expenditures.^8^ The current condition is the result of a steady decline since the 1950s, and it appears even worse considering that the pharmaceutical compartments do constantly absorb and rapidly benefit from major advances from scientific and technological sectors.^9,10^ Such a trend is properly referred to as Eroom’s law,^10^ which is the backward term of Moore’s law coined to describe the exponential increase in the number of transistors that can be placed at a reasonable cost within an integrated circuit.^10^ Metadata analyses are quite effective in dissecting the multitude of causes behind the drug R&D crisis,^10,11^ and they all indicate an emergency that is well perceived at various levels, yet the issue remains at a standstill.^10,11^ In such a context the repurposing/repositioning of approved drugs/investigational compounds outside the original scope of medication is the most realistic strategy to grant increase of any chances of finding new therapeutic tools.^12,13^ Variegate approaches are in use to the drug repurposing/repositioning needs and do include either computational or experimental techniques, as schematically reported in Figure 1.

**Figure 1:**
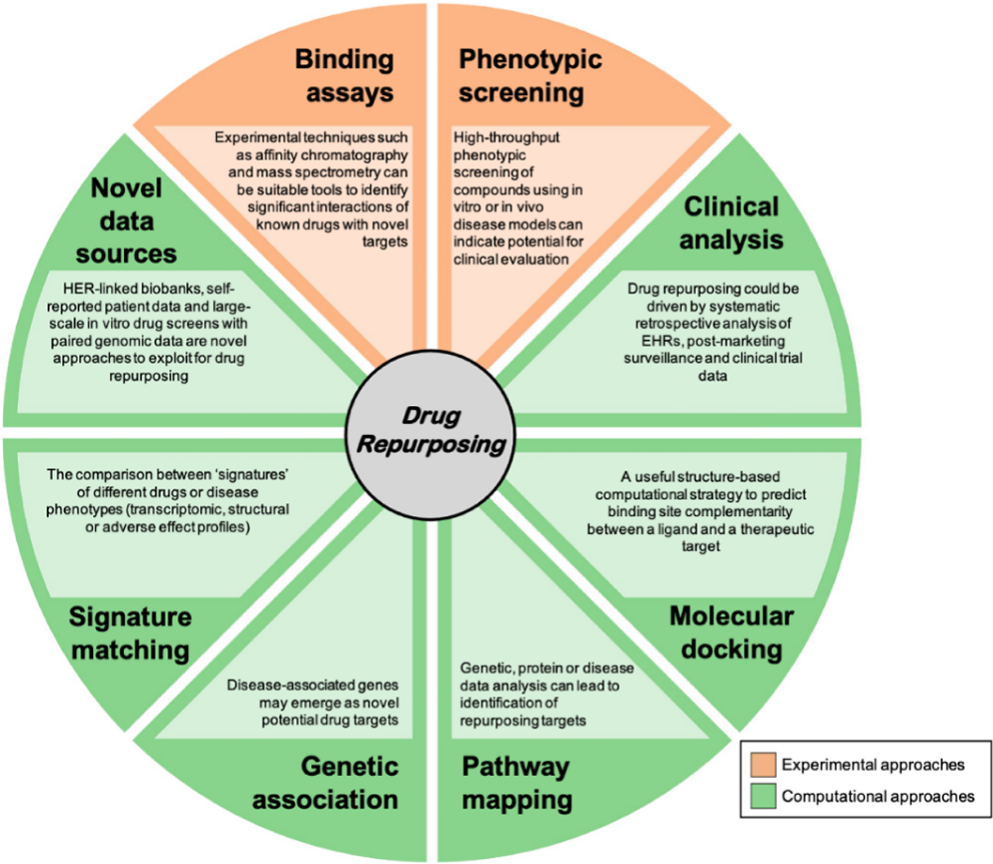
Graphical representation of drug repurposing-associated techniques.^13^

In the context of drug repositioning, we focused our attention on the compound **M16**, which is one of the metabolic intermediates recovered in humans when administered with the β_3_-adrenergic receptor agonist mirabegron^14,15^ for the management of overactive bladder.^16,17^ **M16** is the result of amide cleavage of mirabegron from human esterase enzymes, it is devoid of any biological activity and is usually recovered in the urine and plasma or further processed to the glucuronate **M17** (Figure 2).

**Figure 2:**
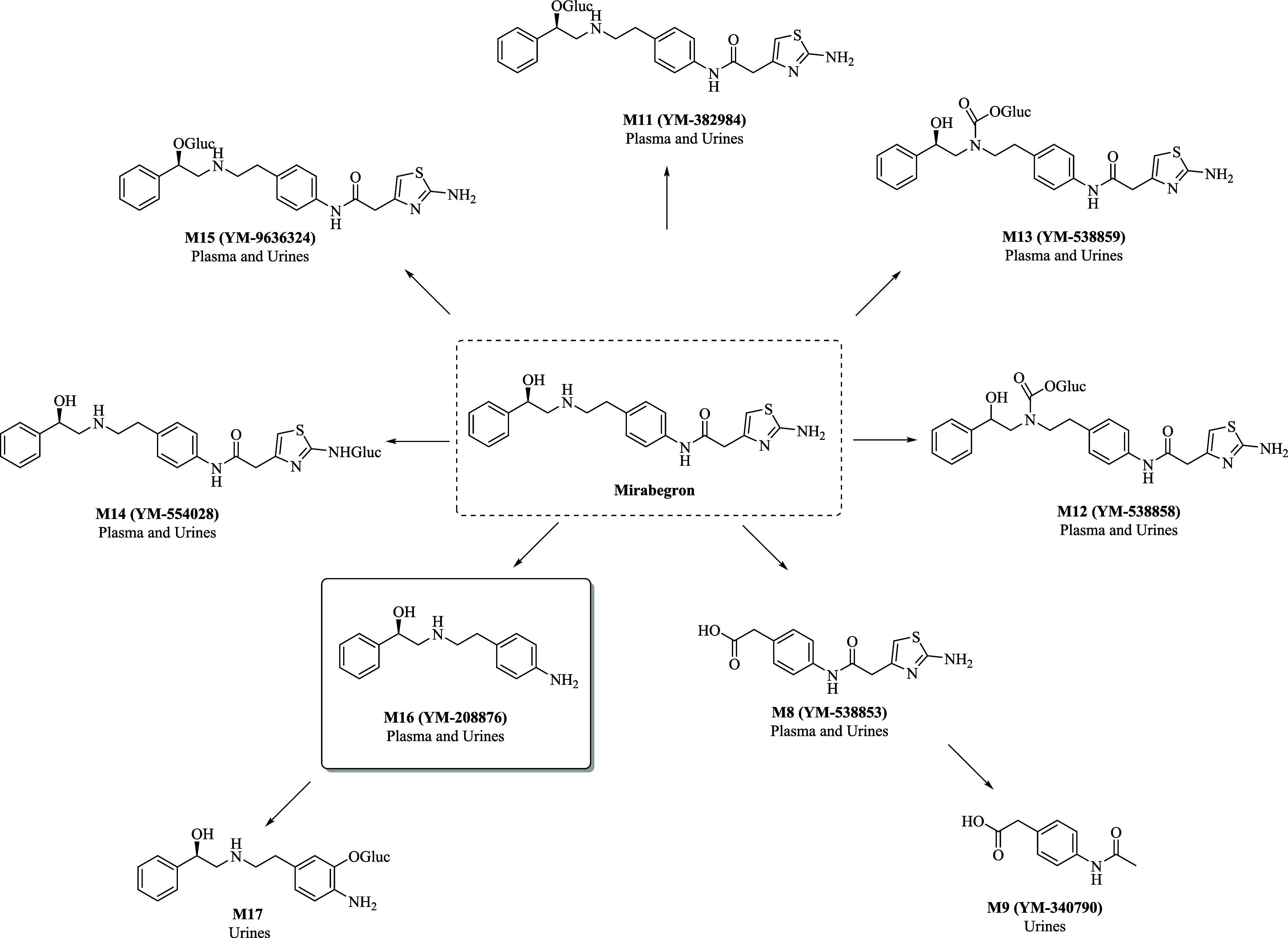
Metabolic pathways of **mirabegron** in humans.^14,15^

Even though **M16** is devoid of any biological activity, it still retains the minimal structural features for binding to any of the G-protein-coupled receptors (GPCRs) β_1_-β_3_ and includes: *i*) an aromatic ring; *ii*) an enantiospecific hydroxyl group; *iii*) a secondary amine; and *iv*) a bulky moiety.^18–22^ Plenty of scientific contributions report wide and detailed structure-activity relationships (SARs) for compounds endowed with affinities for each β-adrenergic receptor (β-AR) subtype, however triggering selective and reliable stimulations *in vivo* from a single signaling pathway remains far from being achieved.^23,24^ The plasticity of the β-AR system,^25,26^ the ligand-directed signaling effect^25,26^ as well as the lack of translationally reliable *in vivo* models (i.e., agonist vs antagonist vs inverse agonist in humans and rodents) are the main hurdles that contribute to make it highly difficult to develop druggable β-ARs directed therapeutics.^23,24^

We took advantage of the R&D gap in the field of β-ARs therapeutics to install on the chemically reactive sites of **M16** with prototypic chemical moieties valuable as modulators of the human (h) expressed Carbonic Anhydrase (CAs; EC 4.2.1.1) with the intent to investigate whether such a manipulation may: *i*) affect the binding ability of **M16** derivatives toward the β-ARs, *ii*) eventually discriminate among each receptor subtype, and *iii*) allowing to make use of such compounds as new tools potentially useful for the management of diseases by the modulation of both pharmacological targets (i.e., the β-ARs and the hCAs).

## Results and Discussion

### Design and Synthesis of Compounds

For the purposes of this study, we considered an investigational synthetic approach based on single-step derivatization of **M16**, obtained from commercial sources as a hydrochloride salt, with freshly prepared aryl- and alkyl-isothiocyanates **1–9** as reported (Schemes 1 and 2).

**Scheme 1:**
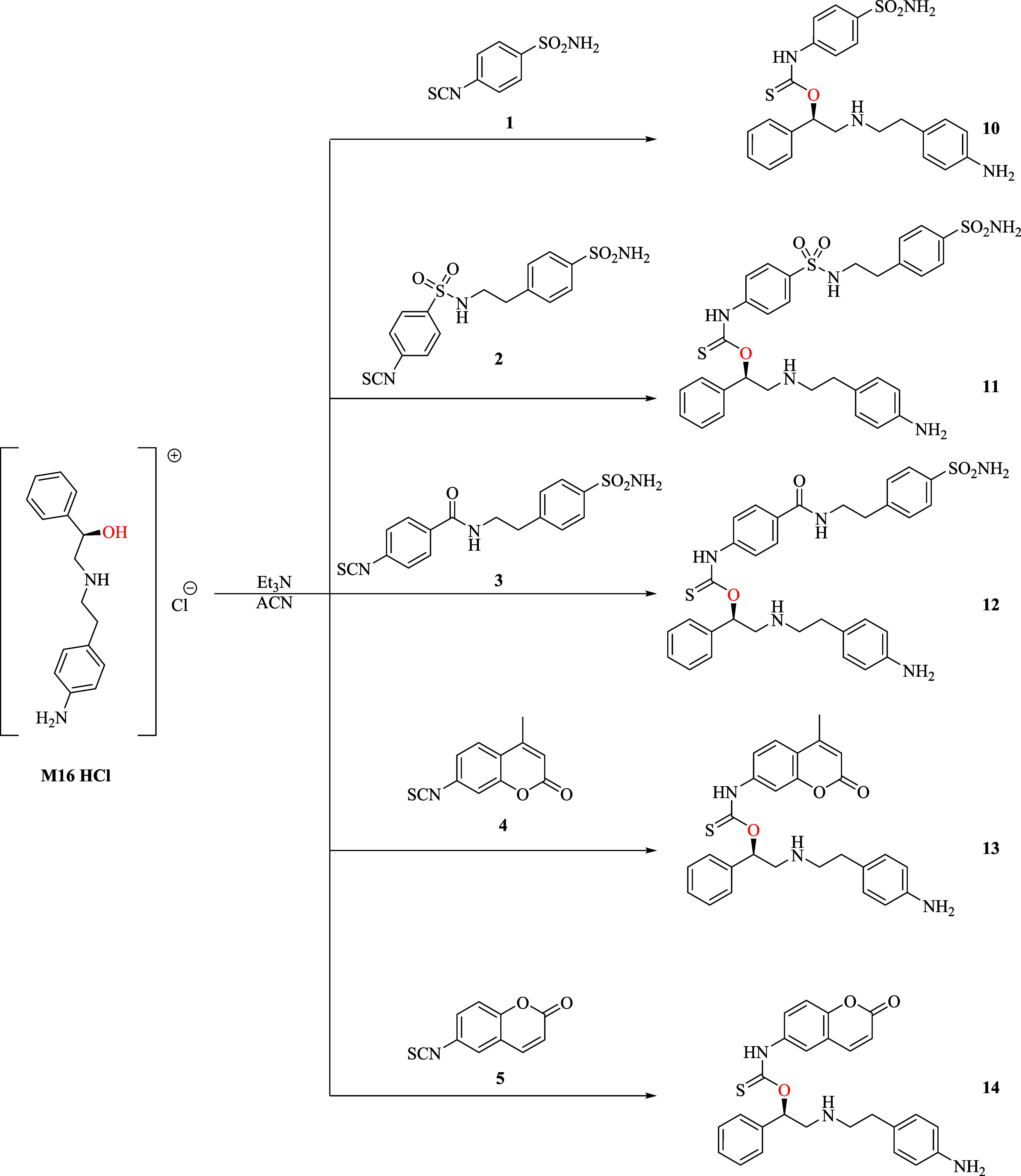
Addition of Aryl-isothiocyanates **1–5** to the Secondary Alcohol of **M16** to Afford **10–14**

**Scheme 2:**
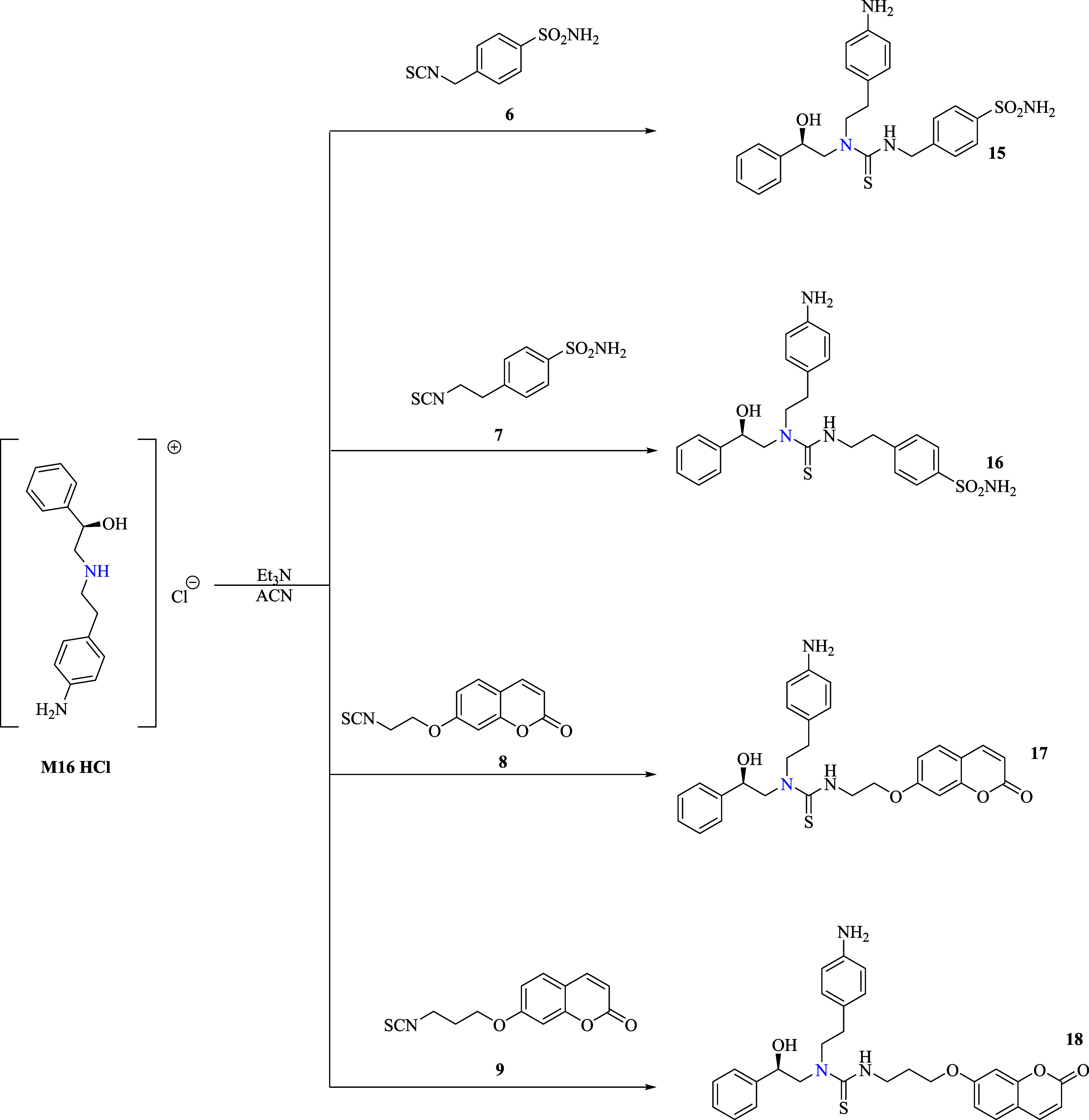
Addition of Alkyl-isothiocyanates **6–9** to the Secondary Amine of **M16** to Afford **15–18**

The appropriate electrophilic isothiocyanates **1–9** were all obtained in high yields from the corresponding primary anilines **1a**–**5a** or alkylamines **6a**–**9a** in agreement with reported synthetic procedures (Schemes 1A,B and 2A–C).^27–30^ Schemes 1 and 2 clearly showed that the addition of **M16** to aryl (i.e., **1–5)** and alkyl (i.e., **6–9)** isothiocyanates under identical reaction conditions afforded the thiocarbamates **10–14** and the thioureas **15–18** in good yields. The reaction outcomes that afforded exclusively the thiocarbamates **10–14** were unexpected and in conflict with our predictive models obtained by machine-learning (ML) approach constructed on single molecular fragment algorithms.^27,28^ Specifically, we dissected **M16** into Mayr’s tabulated molecular fragments bearing single nucleophilic heteroatoms,^31–34^ and their nucleophilicity (*N*) scale was calculated in acetonitrile (ACN) as the solvent at room temperature (i.e., 20 °C) (Figure 3A).

**Figure 3:**
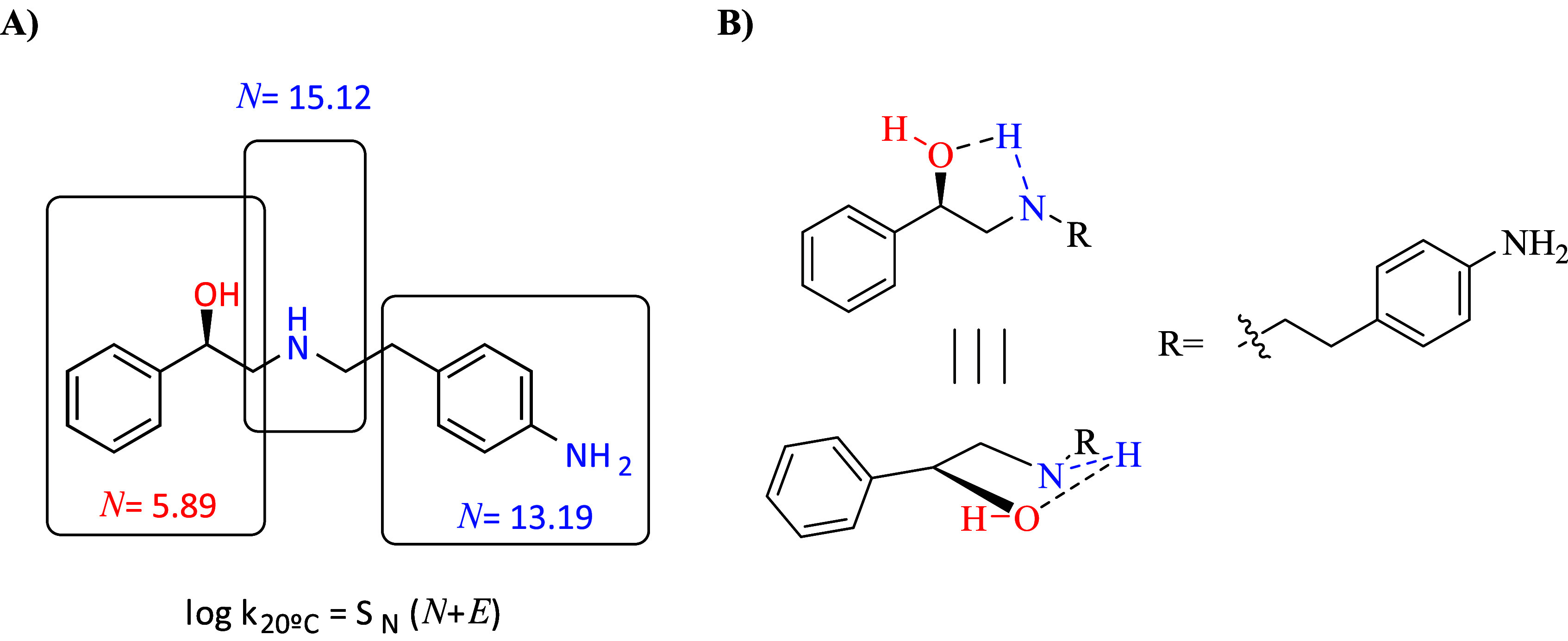
**A**) Predicted nucleophilicity (*N*) values as in agreement with Mayr’s equation in acetonitrile as the solvent and at room temperature; **B**) visualization of the intramolecular five-membered ring in **M16**.

According to the model, thioureas from direct attack of **M16**’s secondary amine or aniline to isothiocyanates were expected to be the preferential adducts or eventually in a mixture to each other when the simulated reaction conditions were applied (i.e., ACN and room temperature). A careful check of TLCs and ^1^H-NMRs of crude products and monitoring of ongoing reactions leading to the thiocarbamates **10–14** provided no evidence for alternative compounds other than the recovered thiocarbamates (see Supporting Information). Close inspection of **M16** allowed us to identify an intramolecular five-membered ring as highly favored (Figure 3B). Such a conformer exposes the benzylic alcohol for addition to the electrophilic isothiocyanates, and it is favorably oriented to transfer its proton to the forming thiocarbamate (Figure 3B and Scheme 3).

Since all thioureido derivatives were exclusively obtained with the alkyl-spaced isothiocyanates **6–9** (see Scheme 2), it is reasonable to speculate that their formation by an intramolecular acyl transferring was triggered from the vicinal secondary amine toward the reactive thiocarbamate group (Scheme 3). Such a transformation is prohibited when aryl isothiocyanates are used instead (see **1–5** in Scheme 1) as the bulkiness of the aryl moieties along with their intense anisotropic effects determines unfavorable orientation and electronic deactivation of the thiocarbamate moiety, which is therefore shielded from any attack of the vicinal secondary amine (i.e., intramolecular fashion; Scheme 3). The same reasons rule out any byproduct formed by a hypothetical intermolecular pathway, despite Mayr’s equation accounting for the **M16**’s aniline being a good nucleophile. Subjection of thiocarbamate **10** to the same reaction conditions (i.e., ACN) and temperatures up to 60 °C for up to 2 h did not afford any secondary products of the thioureidic type, thus confirming our mechanistic hypothesis for the compounds reported in this study.

**Scheme 3:**
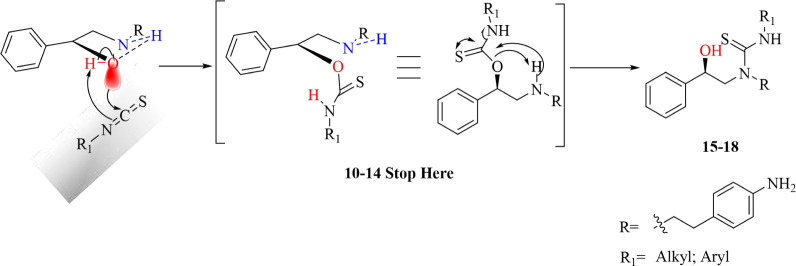
Postulated Mechanisms for the Obtainment of **10–18**

All the final compounds herein reported were purified by silica gel column chromatography using the appropriate eluting mixtures, followed by trituration or recrystallization as needed (see Experimental Section). Full characterization was conducted in solution ^1^H- and ^13^C NMR. Elemental analyses account for a purity grade of ≥95%.

### *In Vitro* Carbonic Anhydrase Inhibition

*In vitro* inhibition profiles of compounds **10–18** and the reference drug acetazolamide (**AAZ**) on the physiologically and catalytically active hCAs I, II, IV, IX, and XII were determined through the stopped-flow CO_2_ hydrase assay^35^ and are reported in Table 1 as *K*_I_ values.

**Table 1:** Inhibition Data of **10–18**, the Reference Drugs Acetazolamide (**AAZ**) and **M16** on hCA Isoforms I, II, IV, IX, and XII by the Stopped Flow CO_2_ Hydrase Assay^35^

*K*_I_ (nM)^a^					
Compound	hCA I	hCA II	hCA IV	hCA IX	hCA XII
**10**	2.1	2.0	104.2	249.0	12.0
**11**	47.3	49.6	145.5	>10000	85.3
**12**	240.3	36.0	98.0	>10000	59.6
**13**	>10000	66.0	224.0	>10000	>10000
**14**	5288.2	75.0	1924.2	>10000	53.0
**15**	9.0	5.0	70.8	452.1	>10000
**16**	17.0	4.8	72.2	505.1	77.0
**17**	>10000	247.3	2488.0	>10000	>10000
**18**	>10000	591.0	1528,1	>10000	45.0
**AAZ**	250	12.1	74.0	25.7	5.7
**M16**	>10000	120.0	>10000	>10000	>10000

a Mean of 3 different assays, by the stopped-flow technique (errors were in the range of the reported values) of ±5–10%.

Based on the reported data in Table 1, structure–activity relationship (SAR) considerations can be drawn:

i) As for the cytosolic hCA I isoform, elongation of **M16** with aryl isothiocyanates **1**, **2**, and **3** to afford the thiocarbamic derivatives **10**, **11**, and **12** respectively, resulted in a progressive reduction of the inhibition potency. The shortest in the series (i.e., compound **10**) was a highly effective inhibitor with a *K*_I_ of 2.1 nM and thus 119-fold more potent than the standard **AAZ**. Although **11** and **12** were equal in length, the observed differences in *K*_I_ values (i.e., *K*_I_s of 47.3 and 240.3 nM respectively) are to ascribe to the interconnecting intramolecular moiety of the sulfonyl type for the former and amide for the latter. Conversely, the coumarin-containing warheads **13** and **14** were ineffective on the hCA I isoform (*K*_I_s of >10000 and 5288.2 nM respectively). A similar *in vitro* kinetic trend was obtained also for the thioureido-containing derivatives. Specifically, elongation of the phenylsulfonamide moiety with one and two carbon atoms as in **15** and **16** halved the inhibition potency toward the hCA I isoform (*K*_I_s of 9.0 and 17.0 nM respectively). The insertion of the coumarin scaffold resulted in suppression of the activity (*K*_I_s of >10 000 nM for **17** and **18**).
ii) Among the thiocarbamate series, compound **10** showed inhibition potency for the hCA II equal to the isoform I (*K*_I_ of 2.0 nM) and again insertion on **M16** of a spacer, as in **11** and **12**, spoiled the ligand affinity (*K*_I_s of 49.6 and 36.0 nM for **11** and **12** respectively). Data in Table 1 for the hCA II account for the opposite kinetic trend of compounds **11** and **12** when compared to the isoform I, being the latter 1.4-fold more potent than its corresponding sulfonyl counterpart **11** (*K*_I_s of 36.0 and 49.6 nM respectively). The thiocarbamic-substituted coumarins **13** and **14** had inhibition potencies slightly lower and comparable to the aryl sulfonamides **11** and **12** (Table 1). As for the thioureido derivatives, it is interesting to note that both primary sulfonamide derivatives **15** and **16** were effective inhibitors of the hCA II with closely matching *K*_I_ values (i.e., 5.0 and 4.8 nM respectively) and comparable to the strongest thiocarbamate derivative **10** (i.e., *K*_I_ of 2.0 nM). The introduction of the coumarin moieties to afford derivatives **17** and **18** was detrimental for the hCA II inhibition as high nanomolar *K*_I_ values were obtained (i.e., *K*_I_s of 247.3 and 591.0 nM respectively).
iii) As for the membrane-associated hCA IV, all compounds tested in this study did not result in particularly effective hCA IV inhibitors and showed *K*_I_ values spanning from the medium nanomolar to low micromolar range and thus higher than the reference **AAZ** (Table 1). Among the thiocarbamate derivatives the shortest **10** was 1.4-fold more effective in inhibiting the hCA IV when compared to its longer counterpart **11** (*K*_I_s of 104.2 and 145.5 nM respectively). Quite interestingly, switching the sulfonamidic linker in **11** with an amide moiety instead to afford **12** resulted in an increase in inhibition potency by up to 1.5-fold (*K*_I_s of 145.5 and 98.0 nM for **11** and **12** respectively). The coumarin derivatives **13** and **14** showed scarse affinity for the hCA IV isoform being their *K*_I_s of 224.0 and 1924.2 nM respectively. Among the thioureido derivatives the sulfonamide-bearing warheads **15** and **16** were the most potent in inhibiting the hCA IV with closely matching *K*_I_ values (i.e., 70.8 and 72.2 nM respectively). Again, the introduction of the coumarin moiety heavily affected the inhibition effectiveness, as clearly demonstrated from the *K*_I_ values of **17** and **18** in Table 1 (i.e., 2488.0 and 1528.1 nM respectively).
iv) The sulfanilamide **10** was the only thiocarbamate derivative endowed with affinity for the tumor-associated hCA IX although its *K*_I_ value of 249.0 nM was 9.7-fold higher than the reference **AAZ** (Table 1). Not dissimilar results were obtained for the thioureido derivatives as the sulfonamide-containing moieties **15** and **16** were high nanomolar hCA IX inhibitors (i.e., *K*_I_s of 452.1 and 505.1 nM respectively) whereas the coumarin resulted to be ineffective with *K*_I_s > 10 000 nM.
v) Better results were obtained for the secondary tumor-associated isoform hCA XII. Data in Table 1 report that the thiocarbamate functionalized with the arylsulfonamide moiety (i.e., compounds **10**, **11**, and **12**) shares a similar trend with the hCA II isoform. Specifically, the shortest derivative **10** was the most potent hCA IX inhibitor with a *K*_I_ of 12.0 nM. Elongation of the CAI-directed warhead, as in **11**, determined reduction of the inhibition potency by up to 7.1-fold, which was partially restored when replacement of the sulfonyl group with an amide was operated. Compound **12** was a more effective hCA XII inhibitor with a *K*_I_ of 59.6 nM, thus 1.4-fold lower when compared to **11**. Introduction of the 4-methyl-7-amino coumarinyl moiety suppressed the affinity for such an enzymatic isoform (i.e., *K*_I_ > 10 000 nM for **13**) whereas the less bulky coumarin substituted at position 6 allowed to regain inhibition potency up to a medium nanomolar level (*K*_I_ of 53.0 nM for **14**). As for the thiourea compounds, the derivatives **16** and **18** were the only ones effective for the hCA XII with *K*_I_s of 77.0 and 45.0 nM respectively. It is interesting to note that for the hCA XII minimal structural changes in the ligands do result in remarkable kinetic effects (Table 1).

Overall, the compounds reported showed distinctive inhibition profiles on the hCAs considered and therefore are ideal experimental tools for the investigations herein pursued.

### β_1_-, β_2_-, and β_3_-Adrenergic Receptor Binding Studies

The final compounds **10–18** were tested *in vitro* at fixed concentrations (i.e., 0.1, 10, and 100 μM) to assess their affinity for the β-AR subtypes expressed on cell membranes, by means of competition studies using the commercially available [^3^H]-CGP 12177 radioligand for the β_1_ and β_2_-ARs^36^ and the [^125^I]-cyanopindolol ([^125^I]-CYP) for the β_3_-AR subtype^37^ respectively. The results obtained are reported in Table S1 and accounted for the synthesized compounds **10–18** to compete with the radioligand binding in a concentration-dependent manner with a clear trend in favor of the β_3_-AR over the β_1_ and β_2_ subtypes. Specifically, all screened compounds were scarcely affine for the β_1_-AR as the amount of displaced radioligand was <20% and <50% at 10 and 100 μM concentrations, respectively (Table S1). The exception was the derivative **17**, which showed an inhibition percentage of specific binding for the β_1_-AR of 68.2 ± 3.5 with no selectivity for the remaining AR subtypes when tested at the maximum concentration (i.e., 85.1 ± 1.4 and 80.6 ± 2.1 for β_2_- and β_3_-ARs respectively, Table S1).

Remarkable β_3_-AR selectivity was featured for **11** and **12** at 1 and 10 μM concentrations despite the fact that they were unable to entirely displace the binding of the [^125^I]-CYP radioligand when tested at 100 μM (Figure 4).

**Figure 4:**
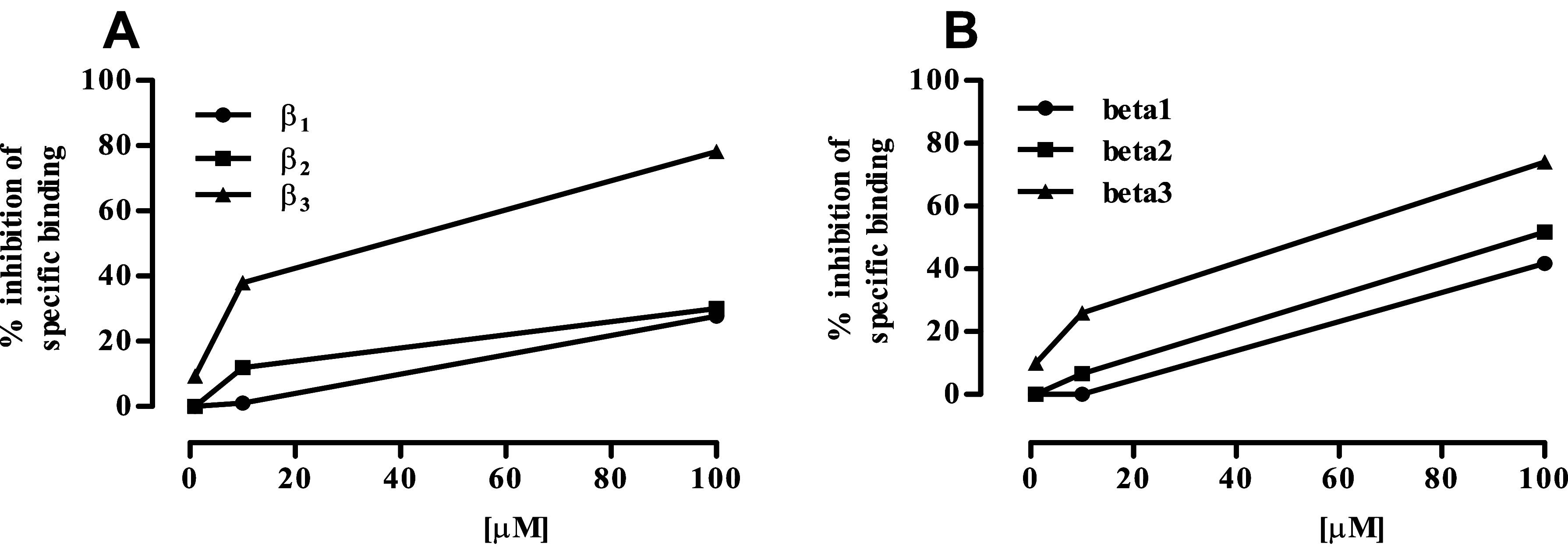
Percent inhibition of specific binding of radioligand to β_1_-, β_2_-, and β_3_-ARs expressed on stably transfected cells for **11 A**) and **12 B**) is shown as a function of the three reported concentrations (i.e., 1, 10, and 100 μM). Values represent the mean from 3 to 4 independent experiments; the error bars are omitted for clarity of the graph.

Among all, the derivative **10** showed good selectivity for β_3_-AR subtype with a specific binding percentage of inhibition of 91.5 ± 1.8 at a 100 μM concentration, whereas the values were far lower for the β_1_ and β_2_ subtypes (i.e., 37.2 ± 2.4 and 65.2 ± 1.8 for β_1_- and β_2_-ARs respectively at 100 μM). Remarkable β_3_-AR selectivity was also reported for compound **14**, which showed at 10 μM concentration inhibition percentages of 9.8 ± 2.9, 14.7 ± 3.0, and 41.5 ± 2.7 for β_1_-, β_2_-, and β_3_-ARs respectively. Far higher percentage values toward the β_1_–β_3_-ARs were obtained when **14** was screened at the maximal concentration of 100 μM (i.e., 27.6 ± 3.1, 62.5 ± 3.2, and 84.2 ± 1.3 for β_1_-, β_2_-, and β_3_-ARs respectively, Table S1). The β_3_-AR subtype selectivity exhibited by **10** and **14** is better appreciated as graphics, as shown in Figure 5.

**Figure 5:**
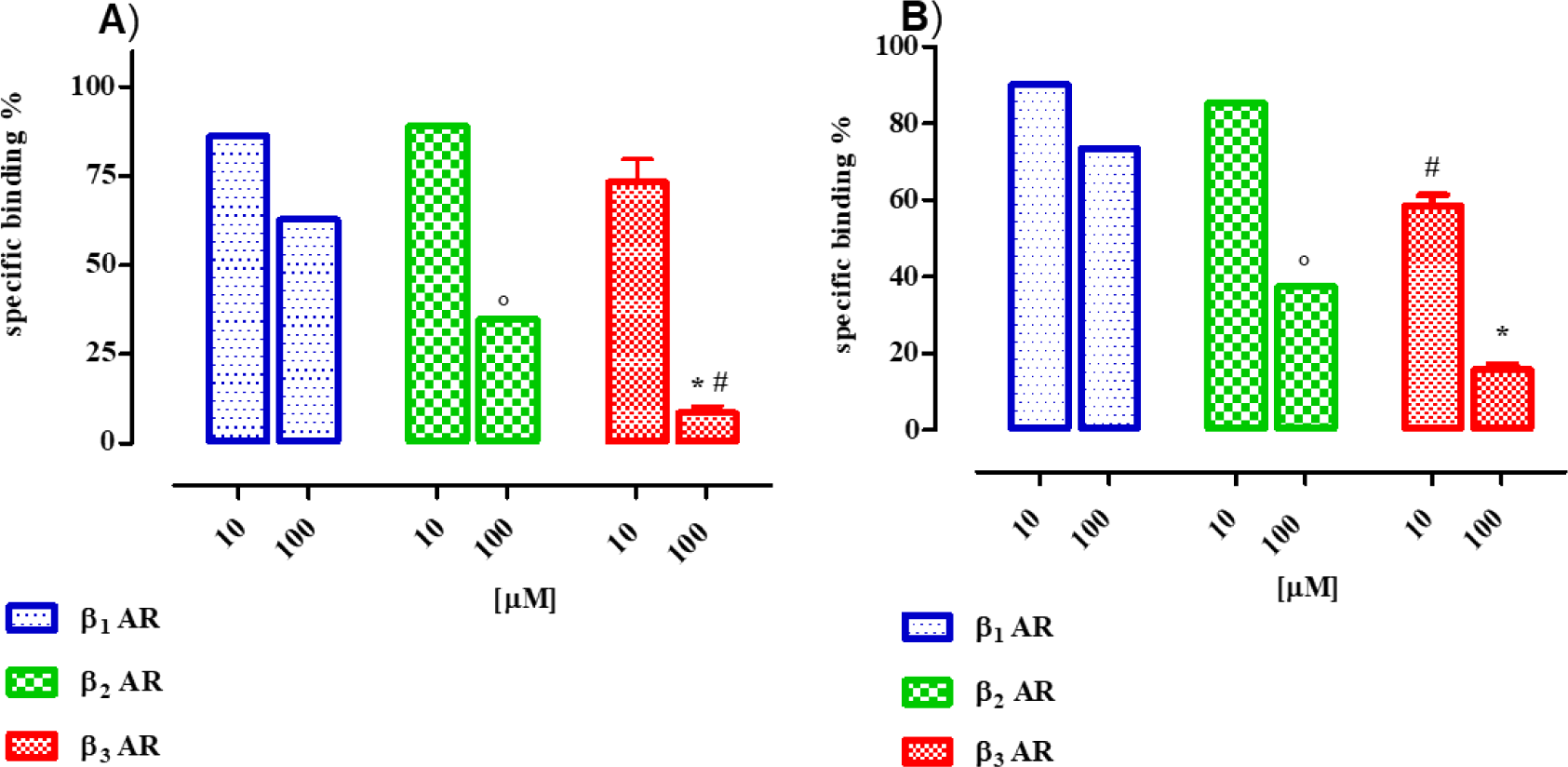
Percent of specific binding of radioligand to β_1_-, β_2_-, and β_3_-ARs expressed on stably transfected cells for **10 A**) and **14 B**) at 10 and 100 μM concentrations. Data are illustrated as a percentage of specific binding toward each β-AR subtype and are presented as mean ± SEM of three to four experiments, each one was performed in duplicate. Parameters were statistically evaluated with one-way ANOVA followed by Tukey’s Multiple Comparison Test. β_2_ vs β_1_ *p* < 0.0001. β_3_ vs β_1_ and β_2_ * ^#^ *p* < 0.0001.

In addition, our experiments showed that **M16** was weaker than **10–18** in binding to β_1_–β_3_-ARs with very closely matching pIC_50_ values (i.e., 4.93 ± 0.27, 5.19 ± 0.09, and 4.94 ± 0.22 for β_1_-, β_2_-, and β_3_-ARs, respectively) and therefore in agreement with the lack of biological activity reported for such a compound (Table S1 and Figure S1).

### β_1_-, β_2_-, and β_3_-Adrenergic Receptor Functional Studies

Functional responses for the best-performing β-AR ligands **10**, **13**, **14**, **16**, and **M16** were determined in each cell line by means of a commercially available intact cell cAMP accumulation assay (AlphaScreen cAMP kit) to allow direct comparison among the three receptor subtypes. The results obtained are summarized in Figure 6 and clearly show that all investigated compounds induced the formation of cAMP. The establishment of the concentration-response curves and the pEC_50_ values (−logEC_50_) accounted for all compounds being of the agonist β-AR type.

**Figure 6:**
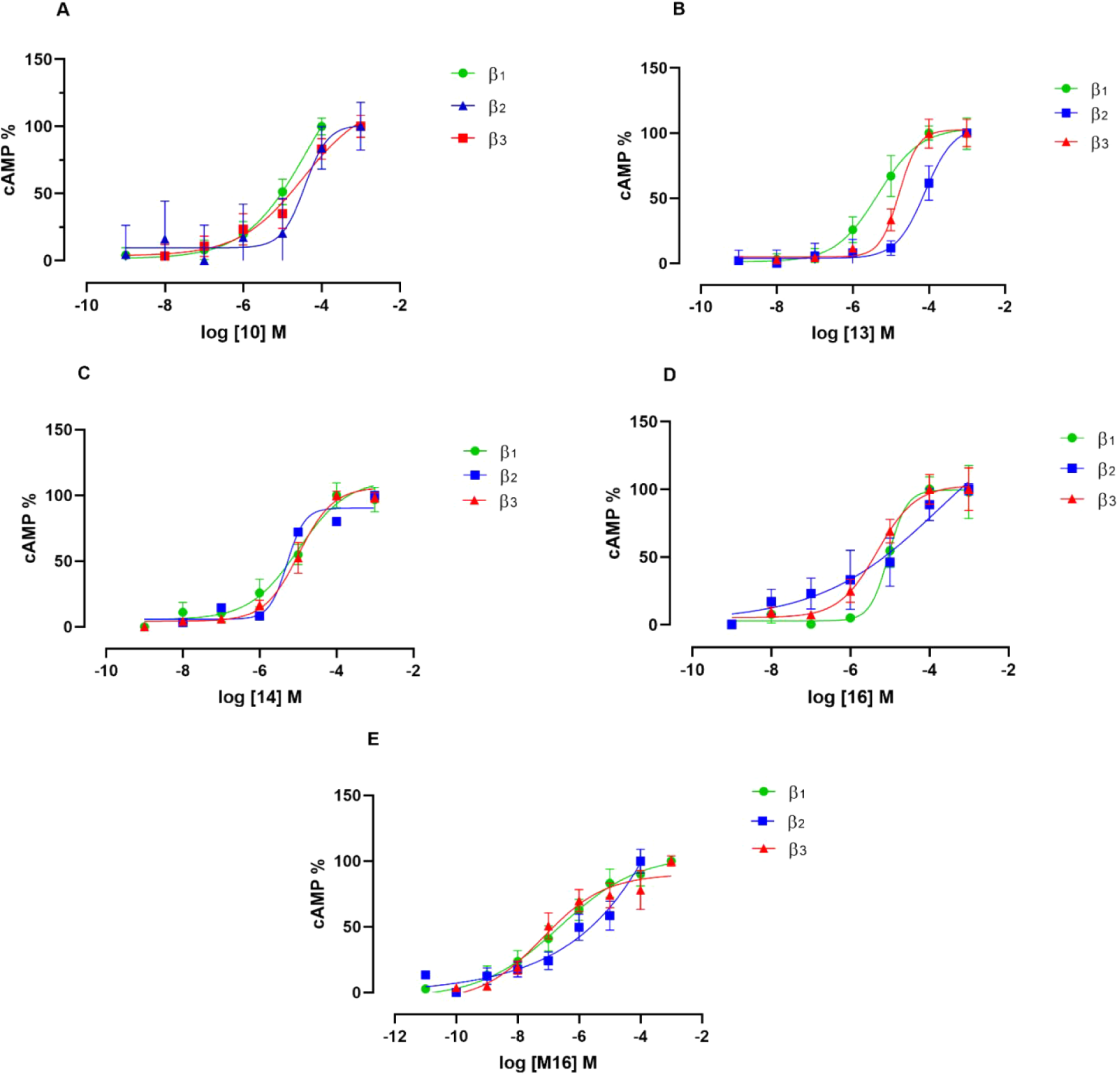
Concentration–response curves of **A**) **10**, **B**) **13**, **C**) **14**, **D**) **16**, and **E**) **M16** cAMP production in HEK293T cells stably expressing β_1_, β_2_, and β_3_-adrenergic receptor subtypes. The results on the *y*-axes were normalized to better clarity. Data are shown as the mean ± SEM of ≥3 independent experiments in triplicates.

The dose–response curves toward the β_2_ receptor are not perfectly resolved in the case of compounds **16** and **M16**. Furthermore, these compounds demonstrate no discernible selectivity for a specific receptor subtype over the others. Particularly, compound **10** induced an increase of cAMP levels from all three receptors equally, with a pEC_50_ value of 4.38 ± 1.38 in β_1_ receptor, 4.43 ± 0.64 in β_2_, and 4.47 ± 0.65 in β_3_ (Figure 6A). Similarly, compound **14** in Figure 6C showed a lack of selectivity for any β-AR subtype although it had enhanced efficacy when compared to **10** (pEC_50_ = 5.00 ± 0.32; 5.29 ± 0.35; 4.98 ± 0.13 in β_1_, β_2_, and β_3_ respectively). As for **13**, a high degree of efficacy for the β_1_-AR when compared to the β_3_ and β_2_ subtypes was observed (pEC_50_ = 5.35 ± 0.25 > 4.78 ± 0.17 > 4.10 ± 0.22 respectively in Figure 6B). The differences in pEC_50_ between β_1_ and β_3_ in compound **16** were negligible (i.e., 5.04 ± 0.09 and 5.32 ± 0.17 for β_1_ and β_3_ respectively; Figure 6D). Quite interestingly, Figure 6E accounted for **M16** with a higher degree of efficacy on β_1_- and β_3_-AR subtypes when compared to the panel of compounds investigated being the associated pEC_50_s of 7.19 ± 0.44 and 6.63 ± 0.52 respectively.

### Intraocular Pressure Determination

The ability of selected compounds to reduce intraocular pressure (IOP) was evaluated *in vivo* in a transient model of ocular hypertension in New Zealand White (NZW) rabbits, and it was compared to the effects induced by the reference drug dorzolamide (DRZ). The data obtained are reported in Figure 7. All derivatives were topically administered as eye drops at 1% w/v concentration, and a vehicle solution composed of 0.9% NaCl + 1% dimethyl sulfoxide (DMSO) was used as the control. The animals were pretreated by injection of 0.05 mL of a hypertonic saline solution (5% NaCl in distilled H_2_O) into the vitreous of both eyes to induce ocular hypertension. The IOP basal value of 20.6 ± 0.3 mmHg was stabilized at 34.2 ± 0.7 mmHg after saline injection.

**Figure 7:**
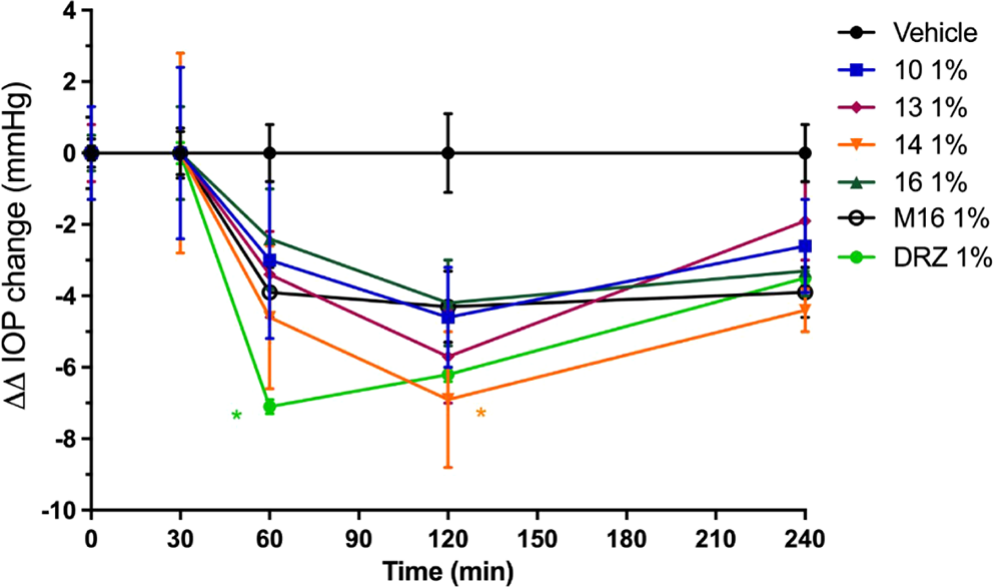
Plot of ΔΔIOP (mmHg) versus time (min) in transient hypertensive rabbit eyes treated with 30 μL of 1% solution of each compound and **DRZ** as the reference drug. *n* = 8 eyes per treatment. Data ± SEM are analyzed with 2 way ANOVA followed by Bonferroni multiple comparison test. *p < 0.05 vs vehicle.

Data in Figure 7 showed that all selected compounds induced appreciable IOP reduction well over the experimental time frame of 4 h. Significant ΔΔIOP reduction was observed with the compound **14**, which exhibited the maximum effect at 120 min post-administration with reduction of IOP in comparison to the vehicle (ΔΔIOP; *p* < 0.05) of −6.9 ± 1.9 mmHg. Although a slight increase in ΔΔIOPs was registered at 240 min (ΔΔIOP= −4.4 ± 0.6 mmHg), an evident biological effect was still persistent. The derivative **13** also showed maximal activity at 120 min post-administration (ΔΔIOP= −5.7 ± 1.3 mmHg) and similar results were reported for **16** (ΔΔIOP= −4.2 ± 1.2 mmHg) and **10** (ΔΔIOP= −4.6 ± 1.4 mmHg). Overall, the set of compounds considered in the experiment retained valuable IOP reduction potencies up to 240 min with very different profiles when compared to those of the reference drug **DRZ** and compound **M16** at the same concentrations (Figure 7). For instance, **M16** showed IOP reduction activity at 60 min post-administration (ΔΔIOP= −3.9 ± 0.7 mmHg) that was maintained almost unchanged over the experimental time frame (ΔΔIOP at 120 min = −4.3 ± 1.0 mmHg; ΔΔIOP at 240 min = −3.9 ± 0.7 mmHg). As for the **DRZ** reference, we registered a maximal activity at 60 min post-administration (ΔΔIOP= −7.1 ± 0.2 mmHg), which steadily diminished up to a ΔΔIOP value of −3.1 ± 0.3 mmHg. The IOP data reported in Figure 7 clearly show that the compounds considered herein do induce IOP effects in a synergistic fashion when compared to their constituent single parts endowed with specific physiological effects.

### Compounds Stability Assessment

The chemical stability of **10**, **13**, **14**, and **16** was assessed by means of HPLC-MS/MS measurements either in phosphate-buffered saline (PBS) solution (i.e., pH ∼ 7.4) and rabbit plasma for all the time necessary to execute the IOP *in vivo* experiments (i.e., up to 240 min). The data obtained are represented in Figure 8 as the chromatographic peak areas recording the common transition of each analyte at various time points (i.e., from the precursor ion, the proton adduct of each compound, to *m*/*z* 120, the common fragment ion).

**Figure 8:**
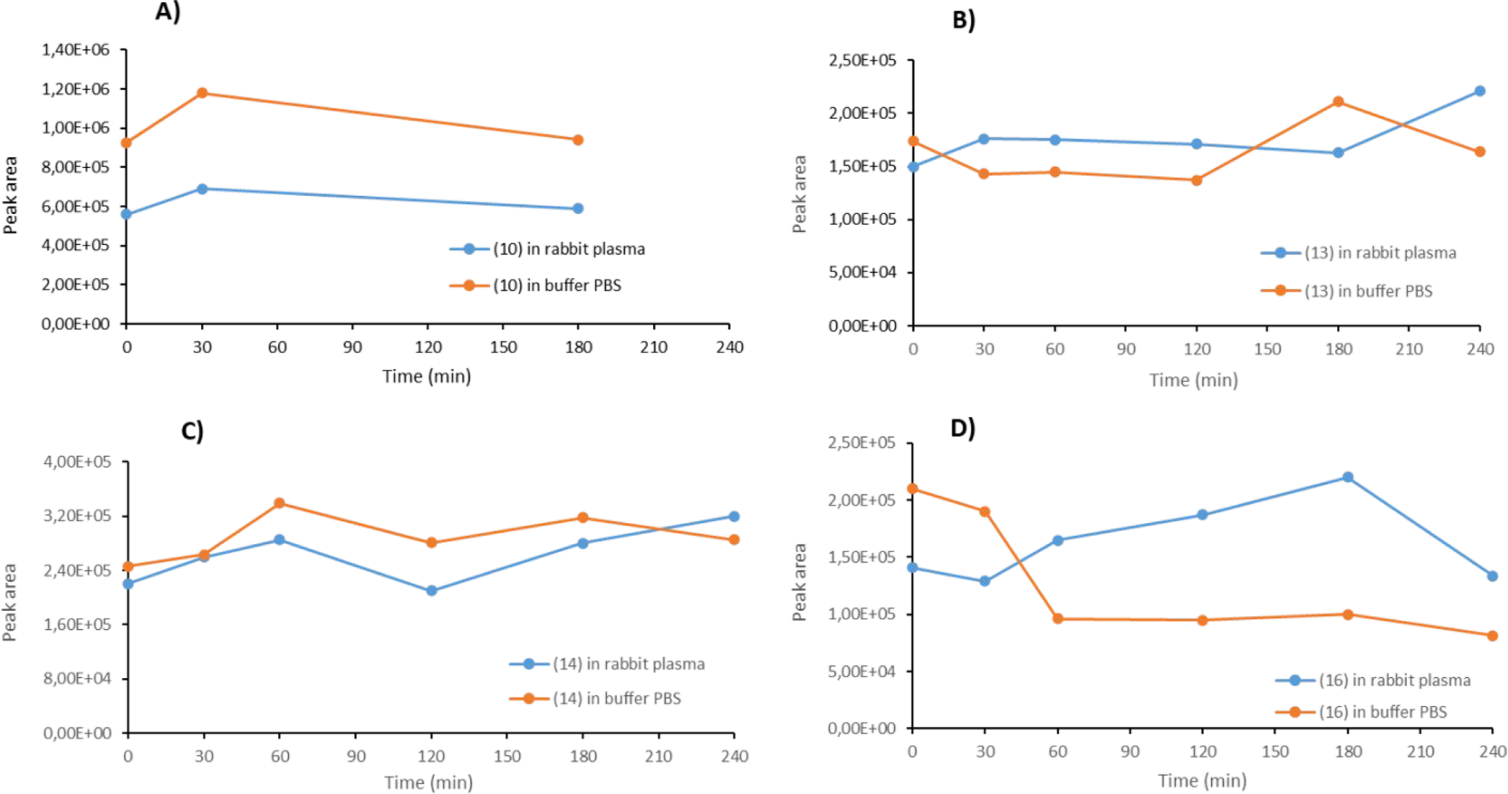
Stability of compounds **A**) **10**, **B**) **13**, **C**) **14**, and **D**) **16** at 1.0 μM concentration in PBS (orange line) and rabbit plasma (blue line) at 37 °C for all of the time necessary to execute the IOP *in vivo* experiments (for compound **10**, stability was assessed until 180 min). Peak area corresponds to the chromatographic peak area (in counts) obtained from the MRM transition as reported in Table S1, measuring the 120 *m*/*z* product ion.

Figure 8A–D shows that each single compound maintained constant concentration values throughout the experimental times either in PBS or after incubation with rabbit plasma at 37 °C. In addition, the concentration trend in both conditions was almost superimposable with small and statistically not significant fluctuations. Samples of **10**, **13**, and **14** revealed traces of **M16**, which were constant in concentration up to the end of the experiments and thus clearly attributable to residuals from synthetic procedures or contamination. The clinically used drug β-AR agonist mirabegron was exposed to the above experimental conditions (i.e., 1.0 μM concentration in PBS and rabbit plasma at 37 °C) for up to 90 min and showed signal intensities that were almost identical for all the time of the experiment. Conversely, human plasma determined the reduction of the drug signal with a proportional increase of metabolite **M16** with no matrix effect observed. Compound **13** was also tested in human plasma in parallel with mirabegron. In this case, the increase in metabolite **M16** was not observed up to 90 min. Overall, HPLC-MS/MS data do agree with the differential esterase/amidase activity of plasmatic proteins (i.e., human vs rabbits)^38–40^ and more importantly clearly account for all the molecules investigated being stable under our experimental conditions, thus giving clear indication of any potential limit to the translational application of our study.

### Wall Tension Assessment of Isolated Retinal Arteries

The effects of **10**, **13**, **14**, and **16** on the wall tension of isolated porcine retinal arterial (PRA) segments precontracted with the thromboxane-A2 analog **U-46619** were assessed by continuous recording with the small wire myography technique. Each PRA was placed in a bath, treated with **U-46619** at 10^–6^ M and once the vasoconstriction effect reached a peak, the selected compound was added at a dose of 10^–6^ M followed by a stepwise increase until the vessel segment was completely dilated (Figure 9).

**Figure 9:**
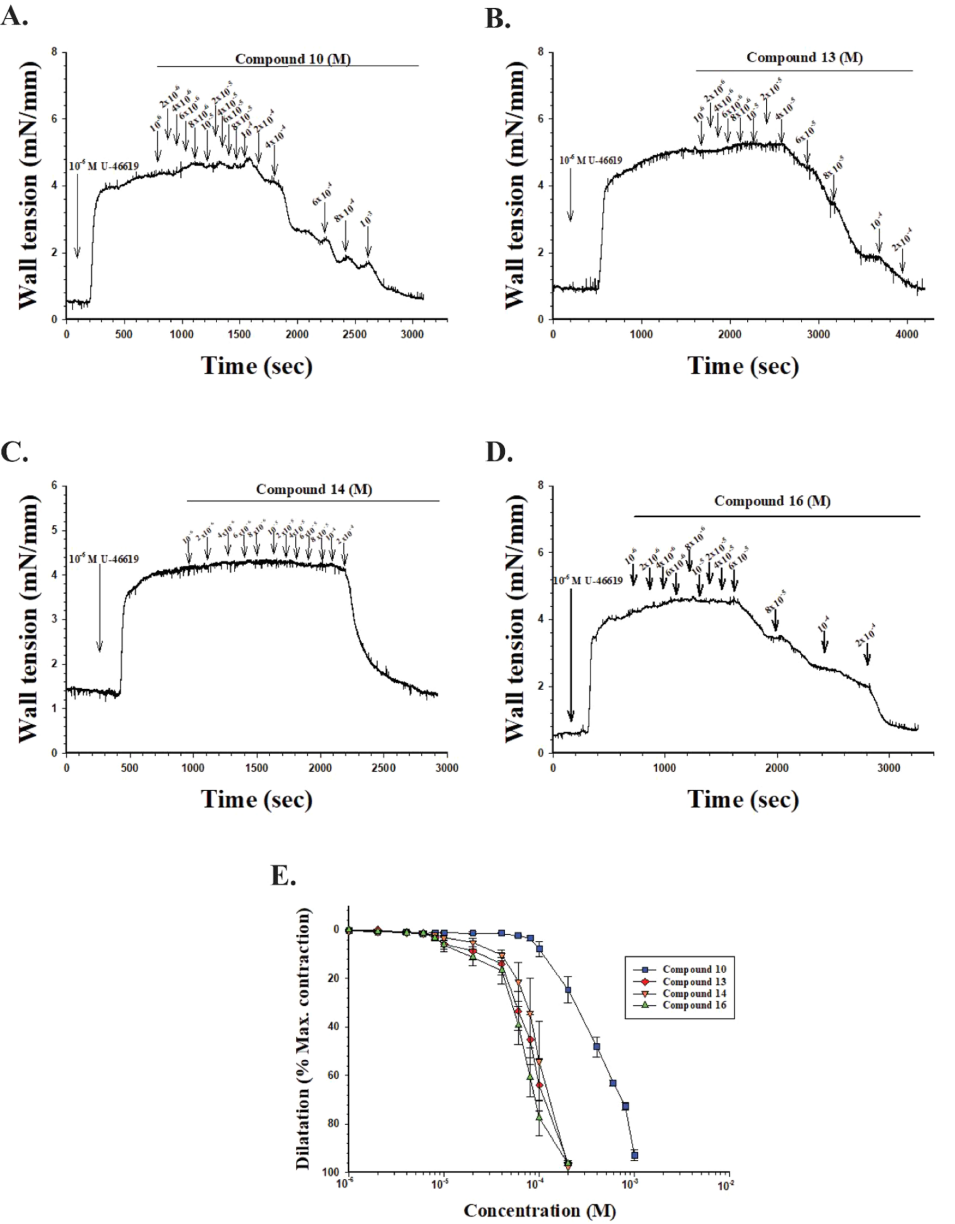
Effects of increasing doses of compounds **10 A**), **13 B**), **14 C**), and **16 D**) on the wall tension of precontracted isolated porcine retinal arterial segments. The arrows indicate the point in time when a new dose of the compound was added, and the values above them report the concentration used in the bath at that point. Panel **E** shows four separate mean concentration–response curves, for each of the compounds tested. The abscissa expresses the log concentration of the compound, and the ordinate reports vasodilation as a percentage of **U-46619**-induced maximum contraction.

Data in Figure 9A–D clearly account for all compounds tested to induce complete vasodilation in all vessel segments (*n* = 7) at varying concentrations as clearly reported by the concentration–response curves in Figure 9E. The concentration dependency of the effect of each compound on vascular tone was assessed by estimating the mean EC_50_ (Table 2) of the vasodilation of each compound from normalized concentration–response curves and calculated by best fit.

**Table 2:** Mean EC_50_ of the Vasodilation Induced by **10**, **13**, **14**, and **16**

Compound	EC_50_(M)
**10**	6.7 × 10^–4^
**13**	9.5 × 10^–5^
**14**	1.1 × 10^–4^
**16**	7.1 × 10^–5^

The mean values of EC_50_ for the four compounds differ significantly (two-way ANOVA, *p* < 0.001) overall, but post hoc comparisons revealed that the mean EC_50_ for the vasodilation induced by compound **10** differed significantly from those of all the other compounds, while that of compound **13** did not differ significantly from that of compound **14** (*p* = 0.59), or that of compound **16** (*p* = 0.06). However, the mean EC_50_ values for the vasodilation induced by compounds **14** and **16** were significantly different (*p* = 0.0136). Thus, compound **10** is significantly less potent as a vasodilator than the other three compounds. Similar experiments with the clinically used β_3_-AR agonist mirabegron and its metabolite **M16** are reported in Figure S2. Specifically, a dose of 60 μM mirabegron was added to the bath, which induced a vasodilation. A concentration–response curve of this effect is presented in Figure S2C, showing the mean ± SEM vasodilation (*n* = 7) as a percentage of the peak vasoconstriction induced by **U-46619**, and it demonstrates that the vasodilation of mirabegron reaches a peak around 10^–4^ M and does not exceed around 40% vasodilation. As for **M16** the compound was added to the tissue bath containing the vessel in a stepwise fashion with increasing doses from 10^–6^ to 10^–3^ M. When the effect of 10^–3^ **M16** on wall tension reached its peak (at about 40% dilation) a single dose of compound **16** was added to the bath (10^–4^ M) which fully dilated the vessel segment. A concentration–response curve of the effect of **M16** on wall tension is presented in Figure S2C, with the mean ± SEM vasodilation (*n* = 7) shown as a percentage of the peak vasoconstriction induced by **U-46619**. As for mirabegron, the vasodilatory effect of **M16** does not exceed more than about 40% vasodilation, but it is a less potent dilator than the clinically used drug as its peak vasodilatory effect was reached at 10^–3^ M concentration.

### Melanosomal Accumulation Assessment

The melanosomal uptake of two best-performing compounds from distinct structural classes, such as coumarin **14** and primary sulfonamide **16**, was performed by using freshly isolated melanosomes from porcine retinal pigment epithelium (RPE). Briefly, each compound was incubated at a final concentration of 4 μM with melanosome suspensions (0.1 μg/μL) in Hanks’ Balanced Salt Solution (HBSS) buffer (pH 7.4) for 2 h at 37 °C. Following incubation, samples were centrifuged, and supernatants were collected. The unbound drug fraction was quantified by LC-MS analysis, enabling the calculation of melanosomal uptake. Control samples lacking melanosomes were used to account for potential nonspecific drug losses. The high- and intermediate-affinity melanin binders propranolol and timolol respectively were used as reference compounds (Figure S3).^41^ Interestingly, compounds **14** and **16** exhibited substantial melanosomal accumulation comparable to that of the known melanin-binding drug propranolol. After 2 h, over 50% of the administered compounds were absorbed by the melanosomes. Significant accumulation within pigmented tissues can be viewed as a promising property of these novel dual-action agents in the context of their ophthalmic applications. In fact, enhanced melanosomal uptake may significantly augment drug exposure in both the anterior and posterior ocular segments and ensure an extended duration of pharmacological effects in these compartments.

## Conclusions

We reported an investigational approach based on the derivatization of the principal metabolite of the β_3_-AR agonist mirabegron, i.e., **M16**, with prototypical CAIs of the primary sulfonamide and coumarin type. The use of the aryl **1–5** and alkyl **6–9** isothiocyanate connecting moieties was revealed an efficient and reliable method to address regioselectivity toward the secondary alcohol and the secondary amine groups in **M16** to afford the carbamates **10–14** and the ureido derivatives **15–18** respectively. The obtained compounds showed marked preferences in inhibiting the hCAs II, IV, and XII, which are constitutively expressed within human eyes and are involved in regulating the aqueous humor dynamics in such organs and increasing the blood flow to the optic nerve and retina.^42–46^ All derivatives showed good binding affinities for the human β-ARs expressed in stably transfected cells, with significant preference for the β_3_-AR subtype. Functional studies accounted for the tested compounds as *pan*-β-AR agonists. Among them, **10, 13, 14**, and **16** were endowed with acceptable selectivity for the β_3_-subtype and were successfully tested in an animal model of transient glaucoma, for the ability to induce appreciable IOP reduction. The stability of **10**, **13**, **14**, and **16** under the assay conditions (i.e., rabbit plasma) and PBS was assessed and undoubtedly indicated that all compounds do not decompose up to the *in vivo* experimental time frame, thus suggesting that the observed *in vivo* IOP effects are attributable to the entire molecules reaching their targets (i.e., hCAs and β-ARs). Since the activity of β-AR subtypes affects vascular function in a variety of vasculatures, including that of the retina,^47–49^ the effects of **10, 13, 14**, and **16** on vascular tones of precontracted isolated retinal arterial segments were assessed and showed full vasodilation compared to mirabegron and **M16** reached only a maximum of 40% of the effect. Additionally, the remarkable melanosomal accumulation of **14** and **16** suggested that the CA/β-AR-directed prototypic structures are excellent candidates to significantly improve their pharmacokinetic profiles for ophthalmic applications.^50–53^ We are therefore confident to speculate that the *in vivo* IOP-lowering effects of our compounds are to be attributed to both the classical reduction of liquor production and the effects on the eye’s vasculature tones mediated by either the CAs or the β-ARs.

## Experimental Section

### Materials and Methods

Anhydrous solvents and all reagents were purchased from Sigma-Aldrich, VWR, and TCI. All reactions involving air- or moisture-sensitive compounds were performed under a nitrogen atmosphere. Nuclear magnetic resonance (^1^H NMR, ^13^C NMR) spectra were recorded by using a Bruker Advance III 400 MHz spectrometer in DMSO-*d*_6_. Chemical shifts are reported in parts per million (ppm), and the coupling constants (*J*) are expressed in Hertz (Hz). Splitting patterns are designated as follows: s, singlet; d, doublet; t, triplet; m, multiplet; bs, broad singlet; dd, doublet of doublets. The assignment of exchangeable protons (NH) was confirmed by the addition of D_2_O. Analytical thin-layer chromatography (TLC) was carried out on Merck silica gel F-254 plates. Flash chromatography purifications were performed on Merck silica gel 60 (230–400 mesh ASTM) as the stationary phase, and ethyl acetate, *n*-hexane, acetonitrile, and methanol were used as eluents. LC-MS grade acetonitrile (ACN), methanol (MeOH), water (H_2_O), ammonium formate (AmFo), formic acid (FoAc), and analytical grade dimethyl sulfoxide (DMSO) were purchased from Sigma-Aldrich (Sigma-Aldrich Italy, Merck, Milan, Italy). Separate stock solutions of chemical standards were prepared at a concentration of 10 mg/mL in DMSO and stored at −20 °C. A first dilution of the molecule solutions was done with ACN to obtain a concentration of 100 ng/μL, from which working solutions at 1 ng/μL were prepared by successive dilution with MeOH/H_2_O 1/1 (v/v) containing 10 mM AmFo. and 0.1% FoAc immediately before their use for analysis of actual samples and for calibration curves. The 1 ng/μL solution was used for optimizing the ESI-MS and MS/MS parameters by infusion in the ESI source.

The HPLC-MS/MS analysis was executed on a Series 200 HPLC, equipped with an autosampler and column oven (PerkinElmer Italy, Monza, Italy) coupled to a 4000 QTRAP mass spectrometer with a TurboV Ion Spray source (Sciex, Toronto, Canada). The LC column was a Kinetex PFP, 2.1 × 100 mm, 2.6 μm (Phenomenex Italy, Castel Maggiore, Bologna, Italy), maintained at 40 °C. The mobile phases were 10 mM AmFo in H_2_O (A) and MeOH (B), both containing 0.1% FoAc. The gradient elution program started at 20% B, held for 0.5 min, then linearly increased to 95% B in 4.5 min, and was maintained for 9 min; then the composition returned to the initial condition in 2 min, and the column was re-equilibrated for 19 min, for a total run time of 35 min. The column flow rate was 0.25 mL/min. The injection volume was 5 μL. The MS parameters were optimized by infusing 1 ng/μL solutions of the analytes into the TurboV Ion Spray source, operating in positive ion mode. The [M + H]^+^ ion was generated for each molecule. The ion spray potential was 4.6 kV, temperature 500 °C, curtain gas, and GS1 and GS2 gas were set at 40, 60, and 55 (arbitrary units), respectively. The gas was high purity N_2_, used also as the collision gas (CAD gas), which was set to medium. The analysis of samples was performed by HPLC-MS/MS in the MRM mode. All compounds reported were ≥95% of purity by elemental analysis.

### General Procedure for the Synthesis of Compounds **10–18**

A stirred suspension of (*R*)-2-((4-aminophenethyl)­amino)-1-phenylethan-1-ol hydrochloride (**M16 HCl**) (200 mg, 1.0 equiv) in anhydrous acetonitrile (5 mL) under an inert atmosphere was treated with triethylamine (1.2 equiv) and the appropriate isothiocyanate **1–9** (1.0 equiv). The reaction mixture was stirred overnight at room temperature and then quenched with slush, and the readily formed precipitate was collected by filtration. Purification by silica gel flash chromatography using MeOH/DCM afforded the titled compounds **10–18**.

### (*R*)-*O*-(2-((4-Aminophenethyl)­amino)-1-phenylethyl)-(4-sulfamoylphenyl)­Carbamothioate (**10**)

Obtained according to the above procedure using 4-isothiocyanatobenzenesulfonamide (**1**).

White solid; yield: 75%; m.p.: 181–183 °C; silica gel TLC Rf: 0.23 (MeOH/DCM 5% v/v); ^**1**^ **H NMR** (400 MHz, DMSO-*d*_6_): δ (ppm) 10.14 (bs, 1H, exchange with D_2_O, CSN*H*), 7.78 (d, 2H, *J* = 8.6 Hz, 2 × Ar-*H*), 7.55 (d, 2H, *J* = 8.4 Hz, 2 × Ar-*H*), 7.48 (d, 2H, *J* = 7.8 Hz, 2 × Ar-*H*), 7.40 (t, 2H, *J* = 7.3 Hz, 2 × Ar-*H*); 7.32 (m, 3H, exchange with D_2_O, SO_2_NH_2_ + Ar-*H*), 6.94 (d, 2H, *J* = 8.4 Hz, 2 × Ar-*H*), 6.52 (d, 2H, *J* = 7.8 Hz, 2 × Ar-*H*), 5.09 (s, 1H, C*H*), 4.91 (s, 2H, exchange with D_2_O, Ar–N*H*_2_), 3.86 (m, 4H, 2 × C*H*_2_), 2.79 (m, 2H, C*H*_2_); ^**13**^ **C NMR** (100 MHz, DMSO-*d*_6_): δ (ppm) 181.8, 147.4, 144.6, 143.2 139.2, 129.7, 128.7, 127.9, 126.4, 126.3, 126.1, 124.2, 114.5, 72.0, 54.8, 46.2, 32.0; **HRMS (** *m* **/** *z* **)** calculated for C_23_H_26_N_4_O_3_S_2_ ([M + H]^+^): 471.1446, found: 471.1444; Elemental analysis, calculated: C, 58.70; H, 5.57; N, 11.91; found: C, 58.76; H, 5.51; N, 11.94.

### (*R*)-*O*-(2-((4-Aminophenethyl)­amino)-1-phenylethyl) (4-(*N*-(4-sulfamoylphenethyl)­Sulfamoyl)­Phenyl)­Carbamothioate (**11**)

Obtained according to the above procedure using 4-isothiocyanato-*N*-(4-sulfamoylphenethyl) benzenesulfonamide (**2**). White solid; yield: 47%; m.p.: 182–185 °C; silica gel TLC Rf: 0.36 (MeOH/DCM 5% v/v); ^**1**^ **H NMR** (400 MHz, DMSO-*d*_6_): δ (ppm) 10.14 (bs, 1H, exchange with D_2_O, CSN*H*), 7.74 (m, 4H, 4 × Ar-*H*), 7.65 (t, 1H, *J* = 5.8 Hz, exchange with D_2_O, SO_2_N*H*), 7.59 (d, 2H, *J* = 8.4 Hz, 2 × Ar-*H*), 7.46 (d, 2H, *J* = 7.8 Hz, 2 × Ar-*H*), 7.38 (m, 4H, 4 × Ar-*H*), 7.29 (m, 3H, exchange with D_2_O, SO_2_N*H*_2_ + Ar-*H*), 6.91 (d, 2H, *J* = 8.4 Hz, 2 × Ar-*H*), 6.50 (d, 2H, *J* = 7.8 Hz, 2 × Ar-*H*), 5.07 (bs, 1H, C*H*), 4.87 (s, 2H, exchange with D_2_O, Ar–N*H*_2_), 3.83 (m, 4H, 2 × C*H*_2_), 3.03 (m, 2H, C*H*_2_), 2.80 (m, 4H, 2 × C*H*_2_); ^**13**^ **C NMR** (100 MHz, DMSO-*d*_6_): δ (ppm) 182.3, 148.0, 145.7, 144.1, 143.7, 143.3, 135.6, 130.3, 130.2, 129.6, 129.3, 128.5, 127.9, 127.0, 126.8, 124.6, 115.1, 72.6, 55.5, 44.7, 36.0, 32.6, 30.7; **HRMS** (*m* **/** *z*) calculated for C_31_H_35_N_5_O_5_S_3_ ([M + H]^+^): 654.1800, found: 654,1803; Elemental analysis, calculated: C, 56.95; H, 5.40; N, 10.71; found: C, 56.89; H, 5.33; N, 10.76.

### (*R*)-*O*-(2-((4-Aminophenethyl)­amino)-1-phenylethyl) (4-((4-sulfamoylphenethyl)­Carbamoyl)­Phenyl)­Carbamothioate (**12**)

Obtained according to the above procedure using 4-isothiocyanato-*N*-(4-sulfamoylphenethyl) benzamide (**3**). White solid; yield: 49%; m.p.: 186–189 °C; silica gel TLC Rf: 0.38 (MeOH/DCM 5% v/v); ^**1**^ **H NMR** (400 MHz, DMSO-*d*_6_): δ (ppm) 9.91 (bs, 1H, exchange with D_2_O, CSN*H*), 8.51 (t, 1H, *J* = 5.4 Hz exchange with D_2_O, CON*H*), 7.78 (m, 4H, 4 × Ar-*H*), 7.45 (m, 6H, 6 × Ar-*H*), 7.38 (t, 2H, *J* = 7.3 Hz, 2 × Ar-*H*), 7.29 (m, 3H, exchange with D_2_O, SO_2_N*H*_2_ + Ar-*H*), 6.92 (d, 2H, *J* = 8.4 Hz, 2 × Ar-*H*), 6.51 (d, 2H, *J* = 7.8 Hz, 2 × Ar-*H*), 5.07 (bs, 1H, C*H*), 4.87 (s, 2H, exchange with D_2_O, Ar–N*H*_2_), 3.81 (m, 4H, 2 × C*H*_2_), 3.54 (q, 2H, *J* = 6.3 Hz, CH_2_), 3.03 (t, 2H, *J* = 6.9 Hz, C*H*_2_), 2.78 (m, 2H, C*H*_2_); ^**13**^ **C NMR** (100 MHz, DMSO-*d*_6_): δ (ppm) 182.4, 166.9, 148.0, 144.9, 144.7, 143.8, 143.1, 130.6, 130.2, 130.1, 129.2, 128.5, 128.2, 127.4, 127.0, 126.8, 124.5, 115.1, 72.6, 55.4, 41.5, 36.0, 32.6, 30.8; **HRMS** (*m* **/** *z*) calculated for C_32_H_35_N_5_O_4_S_2_ ([M + H]^+^): 618.2130, found: 618.2134; Elemental analysis, calculated: C, 62.21; H, 5.71; N, 11.34; found: C, 62.27; H, 5.68; N, 11.30.

### (*R*)-*O*-(2-((4-Aminophenethyl)­amino)-1-phenylethyl)-(4-methyl-2-oxo-2*H*-chromen-7-yl)­Carbamothioate (**13**)

Obtained according to the above procedure using 7-isothiocyanato-4-methyl-2*H*-chromen-2-one (**4**). Pale yellow solid; yield: 81%; m.p.: 183–186 °C; silica gel TLC Rf: 0.36 (MeOH/DCM 5% v/v); ^**1**^ **H NMR** (400 MHz, DMSO-*d*_6_): δ (ppm) 10.36 (bs, 1H, exchange with D_2_O, CSN*H*), 7.72 (d, 1H, *J* = 8.6 Hz, Ar-*H*), 7.55 (bs, 1H, Ar-*H*), 7.46 (d, 2H, *J* = 7.8 Hz, 2 × Ar-*H*), 7.38 (m, 3H, 3 × Ar-*H*), 7.29 (t, 1H, *J* = 7.3 Hz, Ar-*H*), 6.92 (d, 2H, *J* = 8.4 Hz, 2 × Ar-*H*), 6.53 (d, 2H, *J* = 7.8 Hz, 2 × Ar-*H*), 6.31 (s, 1H, Ar-*H*), 5.07 (bs, 1H, C*H*), 4.89 (s, 2H, exchange with D_2_O, Ar–N*H*_2_), 3.84 (m, 4H, 2 × C*H*_2_), 2.78 (m, 4H, 2 × C*H*_2_); 2.44 (s, 3H, Ar–C*H*_3_); ^**13**^ **C NMR** (100 MHz, DMSO-*d*_6_): δ (ppm) 182.1, 161.1, 154.24, 154.18, 148.0, 145.7, 143.8, 130.2, 129.3, 128.5, 127.0, 126.7, 126.0, 120.4, 116.2, 115.1, 113.4, 110.7, 72.6, 59.9, 55.6, 32.6, 19.1; **HRMS** (*m* **/** *z*) calculated for C_27_H_27_N_3_O_3_S ([M + H]^+^): 474.1773, found: 474.1770; Elemental analysis, calculated: C, 68.48; H, 5.75; N, 8.87; found: C, 68.52; H, 5.71; N, 8.83.

### (*R*)-*O*-(2-((4-Aminophenethyl)­amino)-1-phenylethyl)-(2-oxo-2*H*-chromen-6-yl)­Carbamothioate (**14**)

Obtained according to the above procedure using 6-isothiocyanato-2*H*-chromen-2-one (**5**). Off-white solid; yield: 87%; m.p.: 186–188 °C; silica gel TLC Rf: 0.31 (MeOH/DCM 5% v/v); ^**1**^ **H NMR** (400 MHz, DMSO-*d*_6_): δ (ppm) 9.63 (bs, 1H, exchange with D_2_O, CSN*H*), 8.13 (d, 1H, *J* = 9.4 Hz, Ar-*H*), 7.64 (bs, 1H, Ar-*H*), 7.56 (dd, 1H, *J* = 8.8 Hz, 1.9 Hz, Ar-*H*), 7.48 (d, 2H, *J* = 7.8 Hz, 2 × Ar-*H*), 7.39 (m, 3H, 3 × Ar-*H*), 7.31 (t, 1H, *J* = 7.3 Hz, Ar-*H*), 6.95 (d, 2H, *J* = 8.4 Hz, 2 × Ar-*H*), 6.54 (m, 3H, 3 × Ar-*H*), 5.10 (s, 1H, C*H*), 4.91 (s, 2H, exchange with D_2_O, Ar–N*H*_2_), 3.85 (m, 3H, C*H*_2_ + C*H*), 3.71 (s, 1H, C*H*), 2.80 (m, 2H, C*H*_2_); ^**13**^ **C NMR** (100 MHz, DMSO-*d*_6_): δ (ppm) 182.8, 161.1, 151.5, 147.9, 145.2, 144.1, 138.5, 130.98, 130.3, 129.2, 128.4, 126.99, 126.81, 125.5, 119.4, 117.2, 116.9, 115.1, 72.5, 60.0, 55.3, 32.7; **HRMS (** *m* **/** *z* **)** calculated for C_26_H_25_N_3_O_3_S ([M + H]^+^): 460.1617, found: 460.1615; Elemental analysis, calculated: C, 67.95; H, 5.48; N, 9.14; found: C, 68.01; H, 5.54; N, 9.09.

### (*R*)-4-((3-(4-Aminophenethyl)-3-(2-hydroxy-2-phenylethyl)­Thioureido)­Methyl)­Benzenesulfonamide (**15**)

Obtained according to the above procedure using 4-(isothiocyanatomethyl)­benzenesulfonamide (**6**). Pale yellow solid; yield: 63%; m.p.: 182–185 °C; silica gel TLC Rf: 0.32 (MeOH/DCM 5% v/v); ^**1**^ **H NMR** (400 MHz, DMSO-*d*_6_): δ (ppm) 8.23 (bs, 1H, exchange with D_2_O, CSN*H*), 7.81 (d, 2H, *J* = 8.6 Hz, 2 × Ar-*H*), 7.50 (d, 2H, *J* = 8.4 Hz, 2 × Ar-*H*), 7.42 (d, 2H, *J* = 7.8 Hz, 2 × Ar-*H*), 7.37 (t, 2H, *J* = 7.3 Hz, 2 × Ar-*H*), 7.33 (bs, 2H, exchange with D_2_O, SO_2_N*H*_2_), 7.29 (m, 1H, Ar-*H*), 6.90 (d, 2H, *J* = 8.4 Hz, 2 × Ar-*H*), 6.51 (d, 2H, *J* = 7.8 Hz, 2 × Ar-*H*), 5.78 (bs, 1H, exchange with D_2_O, O*H*), 5.03 (bs, 1H, C*H*), 4.94 (m, 2H, C*H*_2_), 4.87 (s, 2H, exchange with D_2_O, Ar–N*H*_2_), 3.86 (bs, 1H, C*H*), 3.72 (m, 2H, 2 × C*H*_2_), 3.55 (m, 1H, C*H*), 2.71 (m, 2H, C*H*_2_); ^**13**^ **C NMR** (100 MHz, DMSO-*d*_6_): δ (ppm) 182.9, 147.9, 145.3, 144.5, 143.4, 130.3, 130.1, 129.1, 128.4, 128.2, 126.95, 126.6, 115.0, 72.2, 60.2, 54.8, 49.1, 32.8; **HRMS** (*m* **/** *z*) calculated for C_24_H_28_N_4_O_3_S_2_ ([M + H]^+^): 485.1603, found: 485.1607; Elemental analysis, calculated: C, 59.48; H, 5.82; N, 11.56; found: C, 59.40; H, 5.86; N, 11.63.

### (*R*)-4-(2-(3-(4-Aminophenethyl)-3-(2-hydroxy-2-phenylethyl)­Thioureido)­Ethyl)­Benzenesulfonamide (**16**)

Obtained according to the above procedure using 4-(isothiocyanatoethyl)­benzenesulfonamide (**7**). White solid; yield: 55%; m.p.: 185–188 °C; silica gel TLC Rf: 0.34 (MeOH/DCM 5% v/v); ^**1**^ **H NMR** (400 MHz, DMSO-*d*_6_): δ (ppm) 7.81 (d, 2H, *J* = 8.6 Hz, 2 × Ar-*H*), 7.74 (bs, 1H, exchange with D_2_O, CSN*H*), 7.49 (d, 2H, *J* = 8.4 Hz, 2 × Ar-*H*), 7.37 (m, 6H, exchange with D_2_O, SO_2_N*H*_2_ + 4 × Ar-*H*), 7.29 (m, 1H, Ar-*H*), 6.88 (d, 2H, *J* = 8.4 Hz, 2 × Ar-*H*), 6.51 (d, 2H, *J* = 7.8 Hz, 2 × Ar-*H*), 5.80 (bs, 1H, exchange with D_2_O, O*H*), 4.99 (bs, 1H, C*H*), 4.80 (s, 2H, exchange with D_2_O, Ar–N*H*_2_), 3.74 (m, 5H, 2 × C*H*_2_ *+* C*H*), 3.44 (m, 1H, C*H*), 3.01 (m, 2H, C*H*_2_), 2.63 (m, 2H, C*H*_2_); ^**13**^ **C NMR** (100 MHz, DMSO-*d*_6_): δ (ppm) 182.4, 147.9, 144.97, 144.5, 143.1, 130.23, 130.20, 129.1, 128.2, 126.94, 126.91, 126.8, 115.0, 72.3, 59.96, 54.5, 47.5, 35.7, 32.7; **HRMS** (*m* **/** *z*) calculated for C_25_H_30_N_4_O_3_S_2_ ([M + H]^+^): 499.1759, found: 499.1755; Elemental analysis, calculated: C, 60.22; H, 6.06; N, 11.24; found: C, 60.28; H, 6.00; N, 11.19.

### (*R*)-1-(4-Aminophenethyl)-1-(2-hydroxy-2-phenylethyl)-3-(2-((2-oxo-2*H*-chromen-7-yl)­oxy)­Ethyl)­Thiourea (**17**)

Obtained according to the above procedure using 7-(2-isothiocyanatoethoxy)-2*H*-chromen-2-one (**8**). Off-white solid; yield: 64%; m.p.: 191–194 °C; silica gel TLC Rf: 0.39 (MeOH/DCM 5% v/v); ^**1**^ **H NMR** (400 MHz, DMSO-*d*_6_): δ (ppm) 8.01 (d, 1H, *J* = 9.4 Hz, Ar-*H*), 7.89 (bs, 1H, exchange with D_2_O, CSN*H*), 7.65 (d, 1H, *J* = 8.9 Hz, Ar-*H*), 7.38 (d, 2H, *J* = 7.6 Hz, 2 × Ar-*H*), 7.33 (t, 2H, *J* = 7.3 Hz, 2 × Ar-*H*), 7.25 (m, 1H, Ar-*H*), 7.10 (d, 1H, *J* = 2.2 Hz, Ar-*H*), 7.03 (dd, 1H, *J* = 8.5 Hz, 2.2 Hz, Ar-*H*), 6.85 (d, 2H, *J* = 8.4 Hz, 2 × Ar-*H*), 6.47 (d, 2H, *J* = 7.8 Hz, 2 × Ar-*H*), 6.31 (d, 1H, *J* = 8.9 Hz, Ar-*H*), 5.82 (bs, 1H, exchange with D_2_O, O*H*), 4.97 (bs, 1H, C*H*), 4.84 (s, 2H, exchange with D_2_O, Ar–N*H*_2_), 4.30 (t, 2H, *J* = 6.0 Hz, C*H*_2_), 3.93 (m, 3H, C*H*_2_ *+* C*H*), 3.65 (t, 2H, *J* = 7.6 Hz, C*H*_2_), 3.45 (bs, 1H, C*H*), 2.64 (t, 2H, *J* = 7.6 Hz, C*H*_2_); ^**13**^ **C NMR** (100 MHz, DMSO-*d*_6_): δ (ppm) 182.8, 162.8, 161.3, 156.5, 147.8, 145.4, 144.4, 130.6, 130.2, 129.1, 128.2, 126.94, 126.93, 126.89, 115.0, 113.7, 113.6, 113.5, 102.5, 72.3, 67.7, 60.1, 54.8, 45.1, 32.6; **HRMS** (*m* **/** *z*) calculated for C_28_H_29_N_3_O_4_S ([M + H]^+^): 504.1879, found: 504.1884; Elemental analysis, calculated: C, 66.78; H, 5.80; N, 8.34; found: C, 66.70; H, 5.76; N, 8.38.

### (*R*)-1-(4-Aminophenethyl)-1-(2-hydroxy-2-phenylethyl)-3-(3-((2-oxo-2*H*-chromen-7-yl)­oxy)­Propyl)­Thiourea (**18**)

Obtained according to the above procedure using 7-(2-isothiocyanatopropoxy)-2*H*-chromen-2-one (**9**). Off-white solid; yield: 72%; m.p.: 196–199 °C; silica gel TLC Rf: 0.42 (MeOH/DCM 5% v/v); ^**1**^ **H NMR** (400 MHz, DMSO-*d*_6_): δ (ppm) 8.01 (d, 1H, *J* = 9.4 Hz, Ar-*H*), 7.70 (bs, 1H, exchange with D_2_O, CSN*H*), 7.65 (d, 1H, *J* = 8.9 Hz, Ar-*H*), 7.38 (d, 2H, *J* = 7.6 Hz, 2 × Ar-*H*), 7.33 (t, 2H, *J* = 7.3 Hz, 2 × Ar-*H*), 7.25 (m, 1H, Ar-*H*), 7.01 (d, 1H, *J* = 2.2 Hz, Ar-*H*), 6.98 (dd, 1H, *J* = 8.5 Hz, 2.2 Hz, Ar-*H*), 6.86 (d, 2H, *J* = 8.4 Hz, 2 × Ar-*H*), 6.47 (d, 2H, *J* = 7.8 Hz, 2 × Ar-*H*), 6.30 (d, 1H, *J* = 8.9 Hz, Ar-*H*), 5.80 (bs, 1H, exchange with D_2_O, O*H*), 4.97 (bs, 1H, C*H*), 4.84 (s, 2H, exchange with D_2_O, Ar–N*H*_2_), 4.17 (t, 2H, *J* = 6.0 Hz, C*H*_2_), 3.71 (m, 5H, 2 × C*H*_2_ *+* C*H*), 3.48 (m, 1H, C*H*), 2.65 (t, 2H, *J* = 7.6 Hz, C*H*_2_), 2.09 (m, 2H, C*H*_2_); ^**13**^ **C NMR** (100 MHz, DMSO-*d*_6_): δ (ppm) 182.6, 162.9, 161.4, 156.5, 147.8, 145.4, 144.5, 130.6, 130.2, 129.1, 128.2, 126.96, 126.91, 115.0, 113.8, 113.5, 113.4, 102.3, 72.4, 67.5, 59.9, 54.6, 43.1, 32.7, 29.5; **HRMS** (*m* **/** *z*) calculated for C_29_H_31_N_3_O_4_S ([M + H]^+^): 518.2035, found: 518.2039; Elemental analysis, calculated: C, 67.29; H, 6.04; N, 8.12; found: C, 67.22; H, 6.0; N, 8.06.

### Carbonic Anhydrase *In Vitro* Assessment

An Applied Photophysics stopped-flow instrument was used to assay the CA-catalyzed CO_2_ hydration activity.^35^ Phenol red (at a concentration of 0.2 mM) was used as an indicator, working at the absorbance maximum of 557 nm, with 20 mM 4-(2-hydroxyethyl)-1-piperazineethanesulfonic acid (HEPES) (pH 7.4) as a buffer, and 20 mM Na_2_SO_4_ (to maintain constant ionic strength), following the initial rates of the CA-catalyzed CO_2_ hydration reaction for a period of 10–100 s. The CO_2_ concentrations ranged from 1.7 to 17 mM for the determination of the kinetic parameters and inhibition constants. Enzyme concentrations ranged between 5 and 12 nM. For each inhibitor, at least six traces of the initial 5–10% of the reaction were used to determine the initial velocity. The uncatalyzed rates were determined in the same manner and subtracted from the total observed rates. Stock solutions of the inhibitor (0.1 mM) were prepared in distilled–deionized water and dilutions up to 0.01 nM were done thereafter with the assay buffer. Inhibitor and enzyme solutions were preincubated together for 15 min at r.t. prior to the assay, to allow for the formation of the E–I complex. The inhibition constants were obtained by nonlinear least-squares methods using PRISM 3 and the Cheng–Prusoff equation^54^ and represent the mean from at least three different determinations. Apart from commercial hCAs I and II, all CA isoforms were recombinant proteins obtained in house, as reported earlier.^55,56^

### Cell Cultures

HEK293T cells were stably transfected for the expression of β_1_ and β_2_ adrenergic receptors as described.^36^

#### Plasmids

The coding region sequence (CDS) of the β_3_ adrenergic receptor was cloned inside the AID- express-puro2 plasmid,^57^ replacing the coding sequence of Activation-Induced Deaminase (AID). We used this plasmid for the presence of an Internal Ribosome Entry Site (IRES) sequence, which allows the expression of a reporter gene (GFP) under the same promoter of our CDS, producing only one mRNA but two different proteins; this feature enables the analysis of the presence and the amount of the β_3_-AR by flow cytometry analysis. For cloning the β_3_-AR, we amplified the CDS from the genomic DNA of HEK293T cells (extracted using a Wizard Genomic DNA Purification Kit). HEK293T cells do not express the β_3_-AR. However, the ADRB3 gene comprises a first big exon and a second tiny one (22 bp). This gene structure allowed us to clone the coding sequence, amplifying the first exon with a couple of primers in which the reverse sequence contains the second exon sequence as a tail. The primers also include NheI and *Bam*HI restriction sites, allowing the cloning of the fragment inside AID-express-puro2 digested by NheI and *Bgl*II. (Forward primer: aaaGCTAGCatgGCTCCGTGGCCTCACG; reverse primer: aaaGGATCCtaagaaactccccaagaagccccgtcgagccgttggcaaa). Sanger sequencing confirmed correct cloning, and the plasmid was used for the transfections.

#### Cell Transfections

HEK293T cells were cultured at 37 °C, 5% CO_2_, in Dulbecco’s Modified Eagle Medium (DMEM, EuroClone Spa, Pero, Milano, Italy) supplemented with 10% fetal bovine serum (FBS; Carlo Erba Reagents, Cornaredo, Milano, Italy), 2 mM L-glutamine (Carlo Erba Reagents), and 1 mM penicillin/streptomycin (Carlo Erba Reagents). Transfections were performed in six-well plates (5 × 10^5^ cells) using Lipofectamine LTX (Invitrogen, Carlsbad, CA, USA) or GeneJuice (Novagen s.r.l., Podenzano, Piacenza, Italy) according to the manufacturer’s instructions. 48 h after transfections, cells were diluted in 96-well plates in medium supplemented with puromycin (1.5 μg/mL) to obtain single clones. Colonies were picked after 10–14 days, and only wells bearing single colonies were expanded for flow cytometer analysis for the presence of GFP. GFP-positive clones were then employed for further analysis.

### Radioligand Binding Studies

#### Membrane Preparation

HEK293T cells stably expressing either the human β_1_, the human β_2_ and the human β_3_-adrenoceptors were used throughout this study.^36^ Cells were grown at approximately 80% confluence and then harvested and homogenized in ice-cold 50 mM Tris-HCl buffer, pH 7.4 with an Ultra-Turrax at half of the maximum speed; the mixture was centrifuged for 10 min at 4 °C and 50,000 rcf. The pellet was carefully resuspended in the same ice-cold buffer, divided into aliquots, and stored at −80 °C. Further details have been described previously.^36^

#### Competition Binding Assay

For the radioligand binding experiments, the frozen samples were thawed and rehomogenized briefly in buffer. A competitive radioligand binding assay was performed in an assay volume of 250 μL under conditions for each ligand that allowed equilibrium conditions. For β_1_ and β_2_ receptor subtypes, [^3^H]-CGP12177 (Revvity Italia Spa, Milano, Italy) was used at the final concentration of 0.2 nM, 25 μg of protein and incubation for 90 min at 25 °C; for β_3_ receptors, [^125^I]-CYP (Revvity Italia Spa) binding was performed at the final concentration of 0.08 nM, using 40 μg of protein and incubation for 60 min at 37 °C. Nonspecific binding was defined using 10 and 100 μM of propranolol for β_1_/β_2_ and β_3_ respectively. All the experiments were performed in duplicates in 96-well plates, and incubations were terminated by rapid vacuum filtration over WhatmanGF/B using a FilterMate harvester (Revvity Italia Spa); each filter was abundantly washed with ice-cold Milli-Q water and dried, and an amount of 20 μL of Microscint 20 cocktail was added. Radioactivity was counted on a TopCount NXT (Revvity Italia Spa) microplate scintillation counter after 4 h. Binding competition data were elaborated using the nonlinear regression curve-fitting function log­(inhibitor) vs response (four parameter) in GraphPad Prism and normalized to the percentage of maximal specific binding for each radioligand. The percentage inhibition values of [^3^H]-CGP12177 and [^125^I]-CYP specific binding were reported.

#### Sample Preparation for LC-MS/MS Experiments

Sample preparation was performed as described by E.Y. Kim et al.^58^ with minor modifications. Frozen rabbit plasma samples were thawed at room temperature, centrifuged at 13,000 rpm for 10 min at 4 °C and 160 μL were transferred into a 1.5 mL Eppendorf tube containing 40 μL of phosphate-buffered saline (PBS). In parallel, 200 μL of PBS were transferred in a new Eppendorf tube. The Eppendorf tubes (plasma and PBS only) were left at 37 °C for 10 min, and then each standard was added to obtain a final 1 μM concentration and maintained at 37 °C. A 20 μL volume was taken at regular time intervals (0, 30, 60, 120, 180, and 240 min) and transferred in a 1.5 mL Eppendorf tube containing 80 μL of ACN with 0.1% FoAc, maintained on ice. After vortex mixing for 30 s, the sample was centrifuged for 10 min at 13,000 rpm in a refrigerated centrifuge (4 °C). A 50 μL volume of the supernatant was transferred in an autosampler vial and diluted with 150 μL of H_2_O with 0.1% FoAc for the LC-MS/MS analysis. For mirabegron and **13** only, a test using human plasma was performed. The procedure was the same as described for rabbit plasma; sampling times were 0, 15, 30, 60, and 90 min.

### Mass Fragmentation Experiments

The MRM transitions and other analyte-dependent parameters are reported in Table S1. Two transitions were acquired for each analyte, and one transition was acquired for mirabegron and its metabolic product **M16**.

### Measurements of Changes in Vascular Tone in Retinal Arterial Segments

Changes in the wall tension of short segments of porcine retinal arterioles were measured with a small vessel myography system, as has been described in detail previously.^46^ Briefly, the pig eyes were obtained from a local abattoir. The pigs were anesthetized with CO_2_ and then put down by exsanguination. All procedures involving animals adhered to the appropriate local and European Union laws and regulations and relevant ethical rules. For the experiments, only one eye was obtained from each animal. Once enucleated, the eyes were placed in a 4 °C oxygenated physiological saline solution (PSS) and transported to the laboratory as quickly as possible. The composition of the PSS used for both the transport of the eyes and as the extracellular bathing solution for myography recordings was as follows (in mM): 112.6 NaCl; 5.91 KCl; 24.9 NaHCO_3_; 1.19 MgCl_2_; 1.18 NaH_2_PO_4_; 2.0 CaCl_2_; 11.5 glucose, all dissolved in double distilled H_2_O. The PSS was oxygenated by a mixture of 95% O_2_ and 5% CO_2_ with the pH maintained at 7.4. A retinal arteriolar segment was obtained by first bisecting the eye with a razor blade at the equator and then removing the anterior segment and the vitreous. The posterior segment was then filled with oxygenated PSS and placed under a stereoscope. A straight arteriole was selected close to the optic disc, and an approximately 2 mm long segment was dissected with retinal tissue extending about 1 mm on either side of the vessel. The segment was then mounted in a DMT630MA wire myograph system (Aarhus, Denmark) to measure the wall tension and contractile activity. The myograph system consisted of four separate tissue baths, with a fluid volume of up to 10 mL, and in each bath there was a force transducer to measure tension to a tungsten wire, 25 μm in diameter, placed in the lumen of a vessel segment. The vessel segment with the wire was transferred to the bath, and the wire ends were attached to the force transducer with screws. A second wire was then guided through the lumen along the top of the first wire and then attached to the system with another set of screws. The heating unit of the myograph system was then turned on and set to a stable temperature of 37 °C. Once all four vessel segments had been placed in the system, the wall tensions of all of them were then continuously recorded. Normalization of wall tension in each segment was then done to ensure that results from vessel segments with different diameters were comparable, and considering the length of each segment. Following normalization, the vessel segments were precontracted with the thromboxane A_2_ analog U-46619 (9,11-dideoxy-9α,11α-methanoepoxy prostaglandin F_2_α) (Cayman Chemicals Inc., Tallinn, Estonia), at a final concentration of 10^–6^ M in the bath. Concentration–response curves for the compounds tested were obtained for changes in wall tension by adding the lowest concentration tested, in most cases 10^–6^ M or lower, to the bathing medium and then adding the next step (e.g., 2 × 10^–6^ M) when the change in wall tension induced by the previous concentration had reached a steady level, until a full effect of the compound on wall tension had been obtained. The estimation of EC_50_ from normalized concentration–response curves was calculated by best fit using GraphPad Prism 10 (GraphPad Software Inc., San Diego, CA, USA).

### Hypertensive Rabbit Intraocular Pressure Lowering Studies

Twenty-four adult male New Zealand White (NZW) rabbits, weighing 2–2.5 kg, were employed in this study. The animals were divided into groups of eight rabbits for each specific treatment, and each rabbit was tested twice after an appropriate period of washout. All experiments were performed in accordance with the European Community Guidelines for the Care and Use of Laboratory Animals (2010/63/EU) and the Italian Legislative Decree 26 (13/03/2014) and the study was approved by the local Animal Care Committee of the University of Florence (Italy) and the Italian Ministry of Health (authorization no. 110/2021-PR). Every effort was made to minimize animal suffering and to reduce the number of animals used. The rabbits were kept in individual cages, and food and water were provided ad libitum. The animals were identified with a tattoo on the ear, numbered consecutively, and maintained on a 12–12 h light/dark cycle in a temperature-controlled room (22–23 °C). All animals were examined before the beginning of the study and were determined to be normal on ophthalmic and general examinations. All the compounds were dissolved in pyrogen-free sterile 0.9% NaCl solution at 1% concentration plus 1% DMSO. Vehicle was 0.9% NaCl and 1% DMSO. The reference compound dorzolamide (**DRZ**) was used at 1% concentration (w/v). The safety assessment ocular test was used to establish if the formulation of each compound was suitable for dosing the animals used in the *in vivo* rabbit model by showing no acute toxicity.^59,60^ Ocular hypertension was induced by the injection of 0.05 mL of sterile hypertonic saline (5% NaCl in distilled H_2_O) into the vitreous bilaterally with local anesthesia provided by one drop of 0.4% oxybuprocaine hydrochloride in each eye 1 min before.^61^ All the compounds were instilled into the lower conjunctival pocket 10 min after the saline injection. Eight different animals were used for each tested compound (8 eyes). One eye was treated with 0.03 mL of drug solution, and the contralateral eye received the same volume of vehicle. IOP was measured using a Model 30 Pneumatonometer (Reichert Inc., Depew, NY, USA). IOP was registered before starting the experimental session to establish basal IOP, and successively 10 min after hypertonic saline injection, to allow the increase of IOP into the suitable experimental range (IOP > 30 and <40 mmHg), and after 60, 120, and 240 min in all groups after drug or vehicle treatment. One drop of 0.4% oxybuprocaine hydrochloride was instilled in each eye immediately before each set of pressure measurements. Data were analyzed with two-way ANOVA followed by the Bonferroni multiple comparison test. *A p* value of *p* < 0.05 was used to identify significance.

### Melanosomal Drug Uptake

Dissection of porcine eyes, collection of pRPE tissue, and melanosome isolation were performed as detailed elsewhere.^62^ The melanin content of isolated melanosomes was determined spectrophotometrically by absorbance at 595 nm (Hidex Sense multiplate reader, Hidex Oy, Finland). On a 96-well plate, 100 μL of melanosome suspension (0.1 μg/μL) in HBSS buffer (5 mM ATP, pH 7.4) was exposed to a 4 μM concentration of the studied drugs (0.2% DMSO) in duplicate. Control samples in HBSS without melanosomes were prepared accordingly in duplicate. The sample plate was incubated at 37 °C on gentle shaking (250 rpm) for 2 h. Two known melanin binders, propranolol (high binder) and timolol (intermediate binder), were used as controls to assess the affinity of novel compounds.^63^ Following drug exposure, the sample plate was centrifuged at 2750 *g* for 10 min and the supernatant was retrieved. The supernatant was diluted 1:4 with acetonitrile and centrifuged at 14,000 *g* for 10 min at 4 °C. The supernatant was retrieved and further diluted with ultrapure water to achieve a 10-fold dilution in the final sample, and sample composition of 50:50 MQ:ACN. Chromatographic separation was performed with Agilent 1290 HPLC system on a Waters UPLC BEH Shield (1.7 μm, 2.1 mm × 50 mm) column at 40 °C. A universal injection volume of 3 μL was chosen for all samples. The mobile phases used were 0.1% formic acid in MQ (A) and 0.1% formic acid in LC-MS grade ACN (B). The following gradient elution at a flow rate of 0.3 mL/min was used: 0–0.5 min 5% B, 0.5–3 min 95% B, 3–4 min 95% B, 4–4.1 min 5% B, 4.1–6 min 5% B. Mass spectrometric measurement was performed with Agilent 6540 Q-TOF mass spectrometer, utilizing electrospray ionization in positive mode for all analytes studied, at capillary voltage 3.5 kV. Nitrogen (Woikoski, Voikoski, Finland) was used as the desolvation gas (10 L/min) at a source temperature of 325 °C. The resulting spectra were analyzed and analytes were quantitated using Agilent Quant-My-Way software.

## Supplementary Material





## Notes

^1^H and ^13^C NMR Spectra of compounds **10–18**; Synthesis of aryl and alkyl isothiocyanates **1–9**; Synthesis of **2a, 3a, 8a** and **9a**; Specific binding inhibition percentage of **10–18** and **M16**; Competition curves of **M16;** MS parameters of compounds **10, 13, 14, 16,** mirabegron and **M16**. Small wire myography for mirabegron and **M16**; Melanosomal uptake for **14** and **16** (PDF)
SMILES representation for compounds (CSV)

## Abbreviations

AAZ: acetazolamide
CA: carbonic anhydrase
CAI: carbonic anhydrase inhibitor
DMSO: dimethyl sulfoxide
ESI: electrospray ionization source
HRMS: high-resolution mass spectrometry
K_*i*_: inhibitor constant
MS: mass spectrometry
NMR: nuclear magnetic resonance
SAR: structure–activity relationship
TLC: thin-layer chromatography
