# Supplementary material for: Repurposing Drug Metabolites into Dual β‑Adrenergic Receptor–Carbonic Anhydrase Modulators as Potential Tools for Ocular Disorders
Source: J Med Chem. 2025 Aug 26;68(17):18579–96. doi: 10.1021/acs.jmedchem.5c01459 (PMC12434671; doi:10.1021/acs.jmedchem.5c01459)
Supplement: Supplementary file 1 [file jm5c01459_si_001.pdf]

Supplementary material for

Repurposing Drug Metabolites Into Dual β-Adrenergic Receptor–Carbonic Anhydrase Modulators As Potential Tools For Ocular Disorders

Andrea Ammara,[1#] Alessandra Carone,[1#] Laura Lucarini,[2*] Silvia Sgambellone,[2] Silvia Marri,[2] Serafina Villano,[2] Rosanna Matucci,[2] Gerta Luga,[2] Chiara Fittipaldi,[2] Riccardo Pecori,[3] Giuseppe Pieraccini,[4] Claudia Di Serio,[5] Andrea García-Llorca,[6] Thor Eysteinsson,[6] Stanislav Kalinin,[7] Julius Aleksi Olavi Viita,[7] Arto Urtti,[7] Fabrizio Carta,[1*] Silvia Selleri,[1*] and Claudiu T. Supuran[1]

[1] NEUROFARBA Department, Sezione di Scienze Farmaceutiche e Nutraceutiche, University of Florence, Via Ugo Schiff 6, 50019 Sesto Fiorentino, Florence, Italy.

[2] Department of Neuroscience, Psychology, Drug Research and Child Health (NEUROFARBA), Section of Pharmacology, University of Florence, Viale Gaetano Pieraccini, 6, 50139 Florence, Italy.

[3] German Cancer Research Center (DKFZ), Im Neuenheimer Feld 280, 69120 Heidelberg, Germany.

[4] Department of Health Sciences, CISM Mass Spectrometry Centre, University of Florence, Viale Gaetano Pieraccini 6, 50139, Florence, Italy.

[5] Experimental and Clinical Medicine Department, Geriatric Intensive Care Unit, University of Florence, Azienda Ospedaliera Universitaria Careggi, Viale Gaetano Pieraccini 6, 50139, Florence, Italy.

[6] Department of Physiology, Biomedical Center, Faculty of Medicine, University of Iceland, 101 Reykjavík, Iceland.

[7] School of Pharmacy, Faculty of Health Sciences, University of Eastern Finland, Yliopistonrinne 3, Kuopio 70211, Finland

[#] These Authors contributed equally

Corresponding Authors

Laura Lucarini (L.L.); e-mail: laura.lucarini@unifi.it

Fabrizio Carta (F.C.); e-mail: fabrizio.carta@unifi.it

Silvia Selleri (S.S.); e-mail: silvia.selleri@unifi.it

Index

^{1}H and ^{13}C NMR Spectra of compounds **10-18**	S3-S11
Synthesis of aryl and alkyl isothiocyanates **1-9**	S12-S16
Synthesis of **2a, 3a, 8a** and **9a**	S17-S19
Specific binding inhibition percentage of **10-18** and **M16**	S20
Competition curves of **M16**	S21
**Table S1.** MS parameters of compounds **10, 13, 14, 16,** mirabegron and **M16**	S22
Small wire myography for mirabegron and **M16**	S23
Melanosomal uptake of **14** and **16**	S24
References	S25-S26

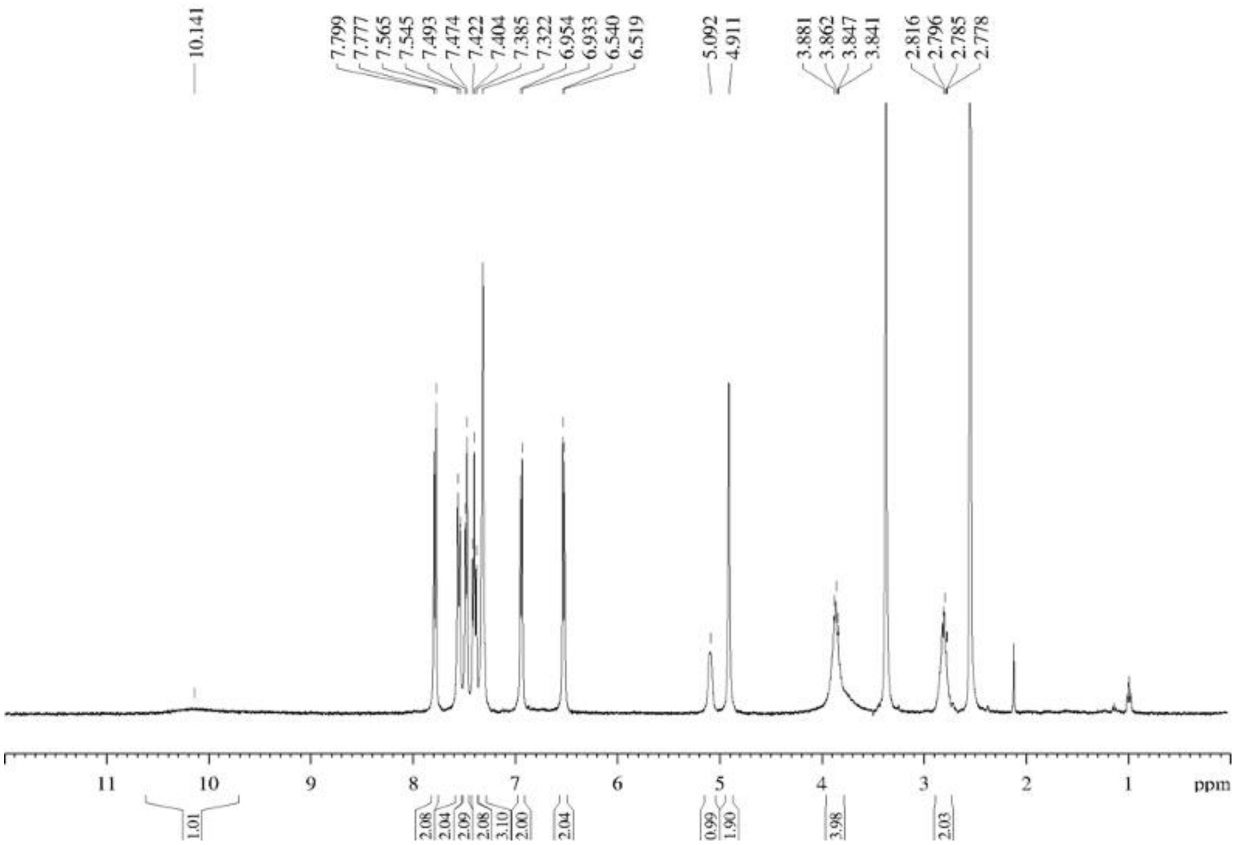

^{1}H NMR spectrum of compound **10** (400 MHz, DMSO-d_6)

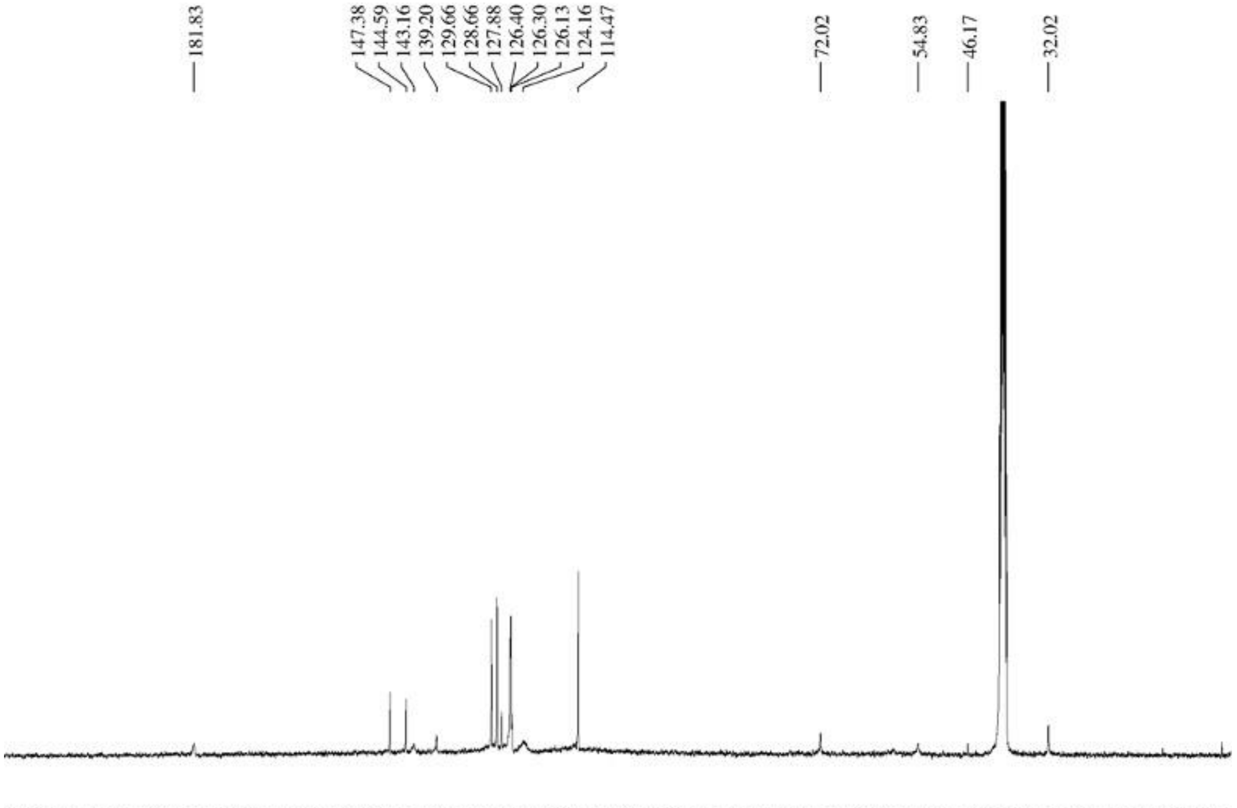

^{13}C NMR spectrum of compound **10** (100 MHz, DMSO-d_6)

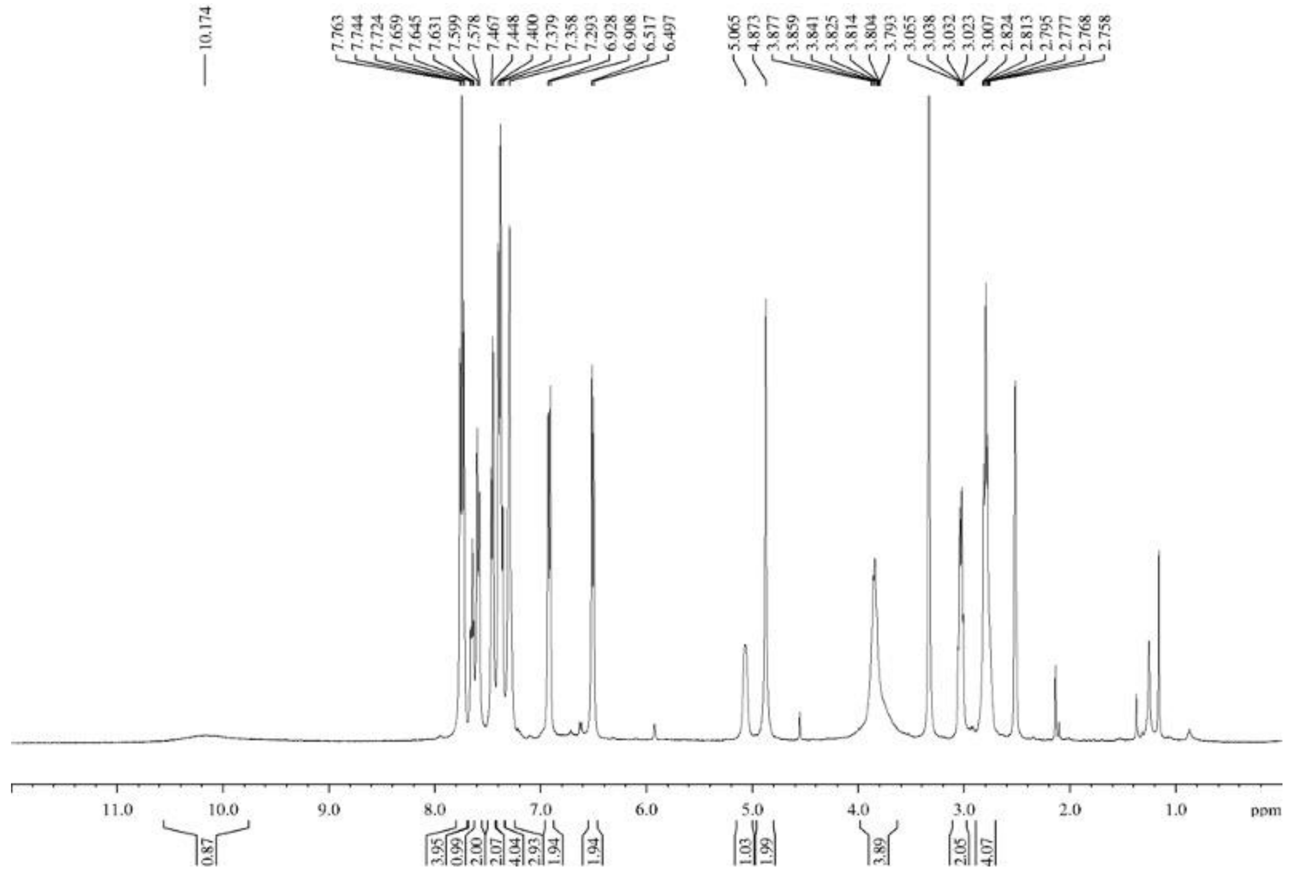

^{1}H NMR spectrum of compound **11** (400 MHz, DMSO-d_6)

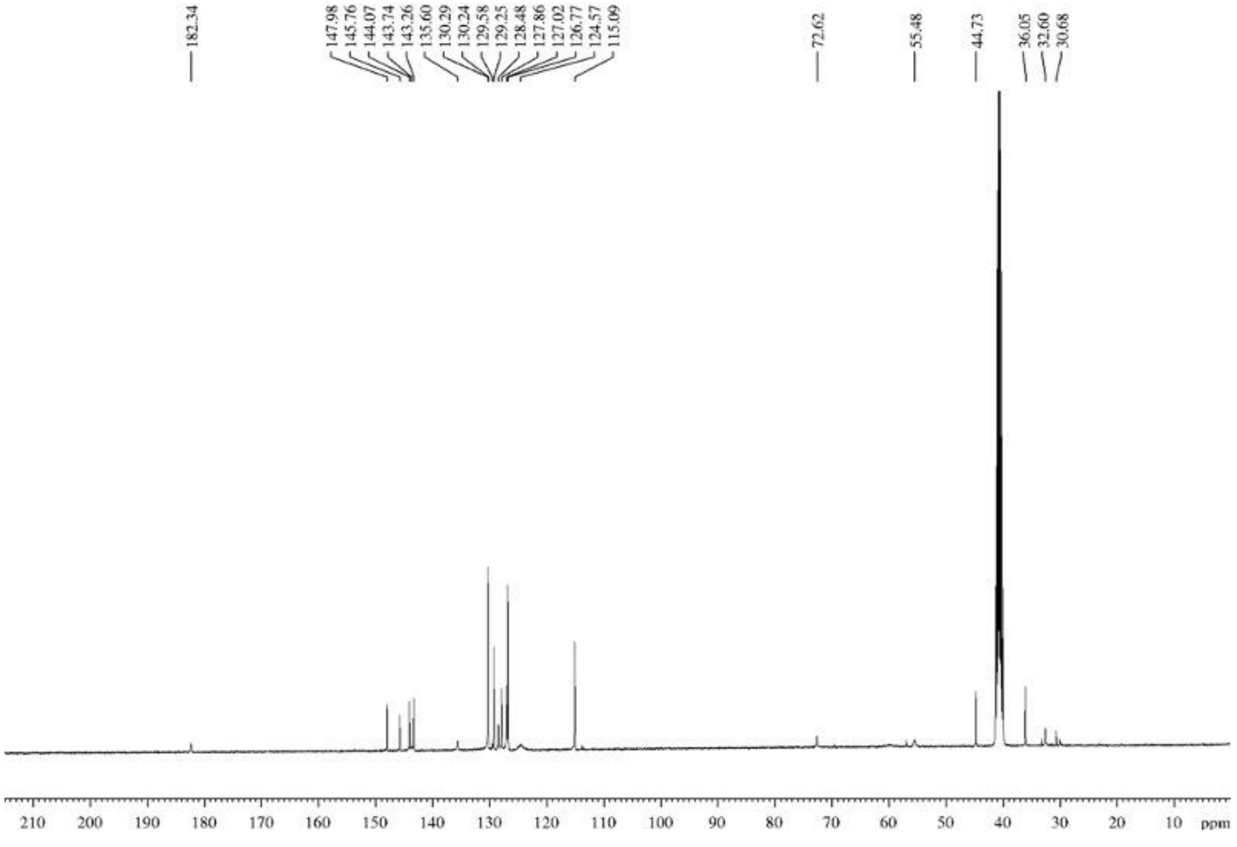

^{13}C NMR spectrum of compound **11** (400 MHz, DMSO-d_6)

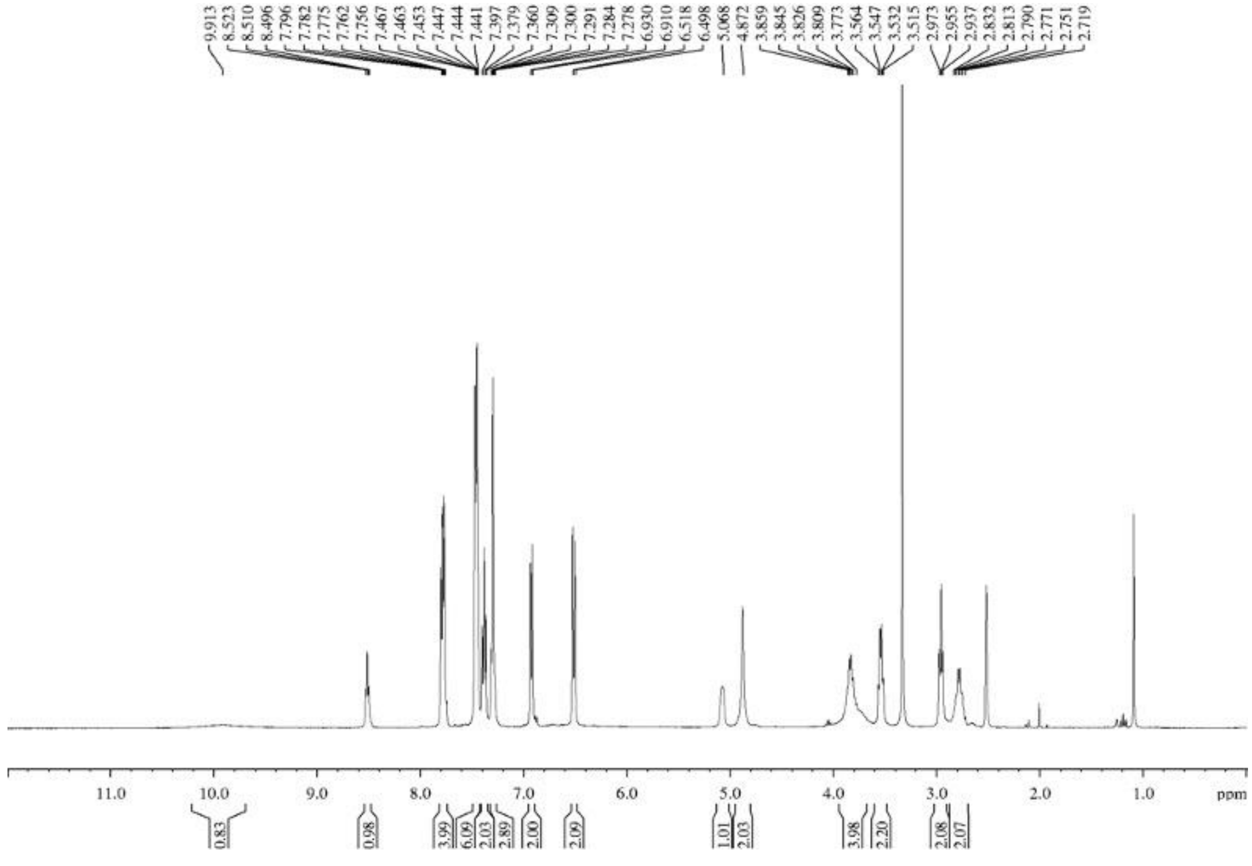

^{1}H NMR spectrum of compound **12** (400 MHz, DMSO-d_6)

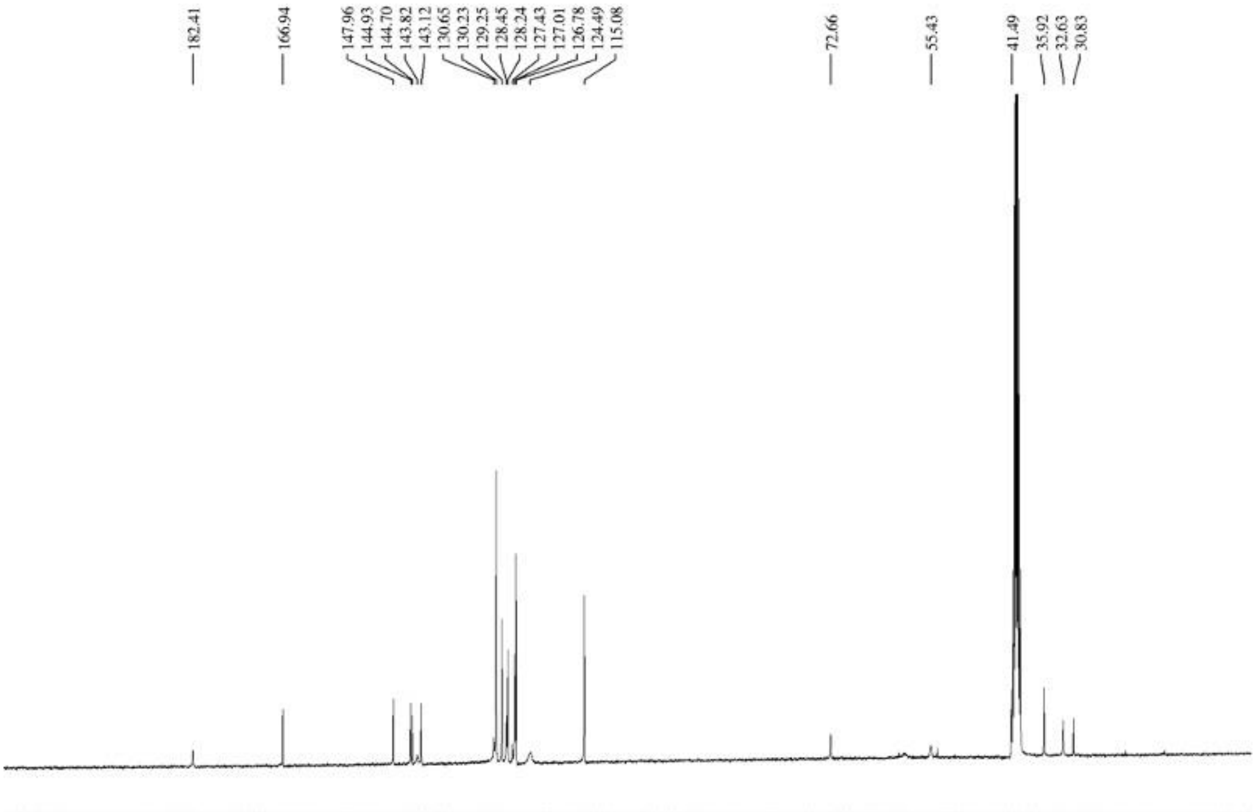

^{13}C NMR spectrum of compound **12** (400 MHz, DMSO-d_6)

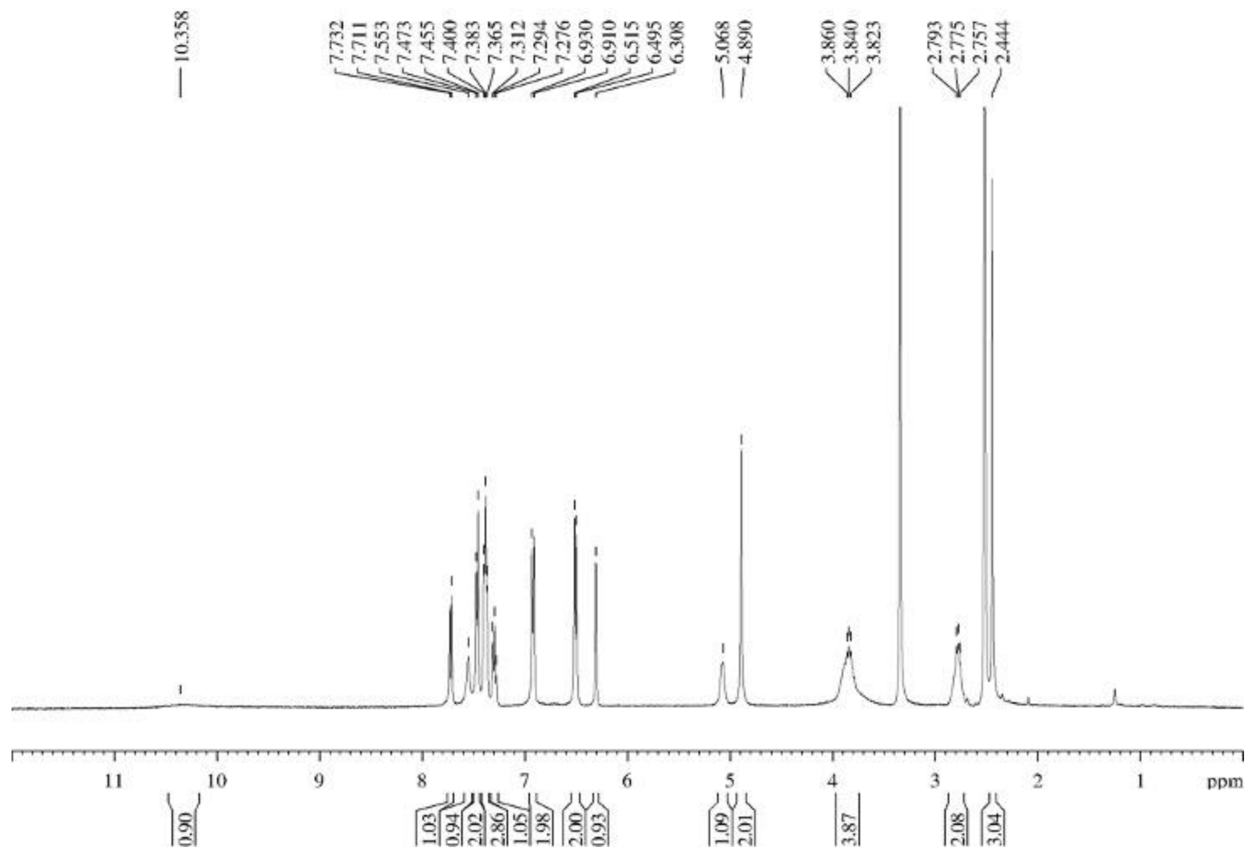

^{1}H NMR spectrum of compound **13** (400 MHz, DMSO-d_6)

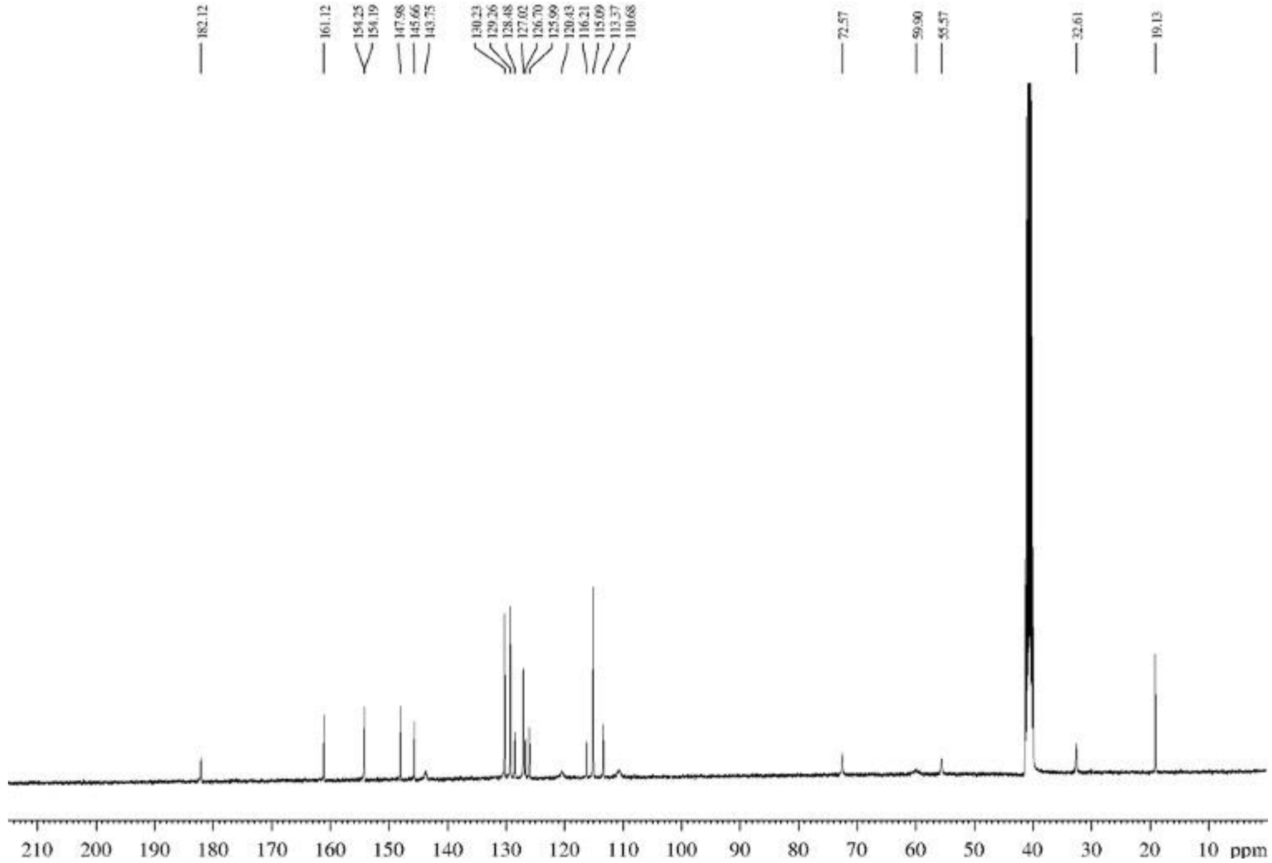

^{13}C NMR spectrum of compound **13** (100 MHz, DMSO-d_6)

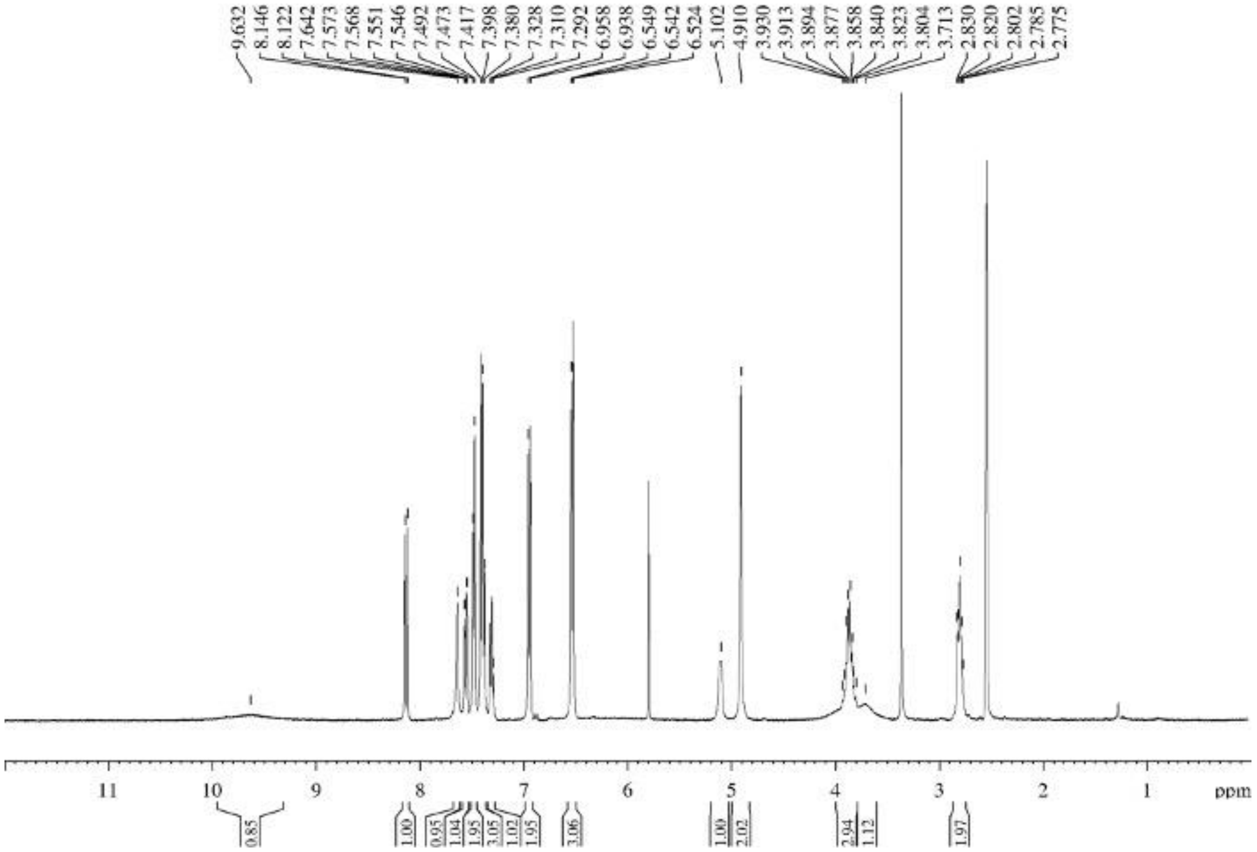

^{1}H NMR spectrum of compound **14** (400 MHz, DMSO-d_6)

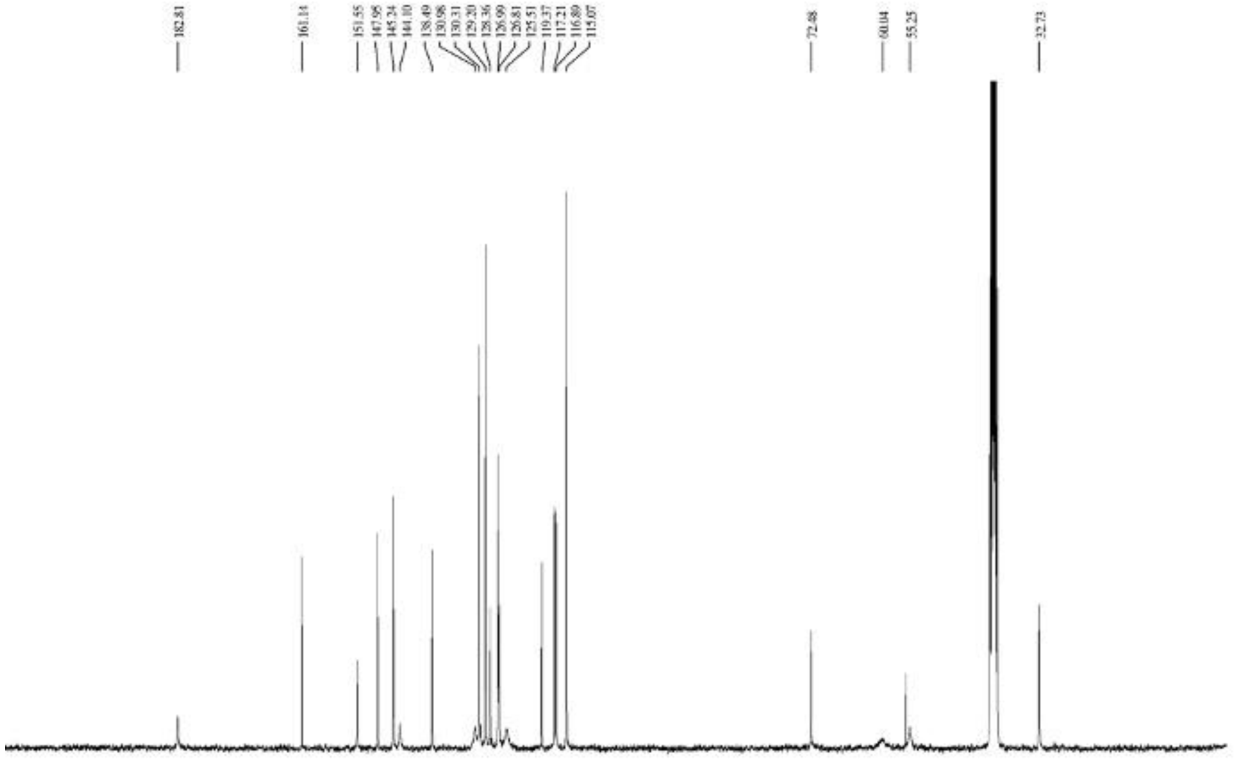

^{13}C NMR spectrum of compound **14** (100 MHz, DMSO-d_6)

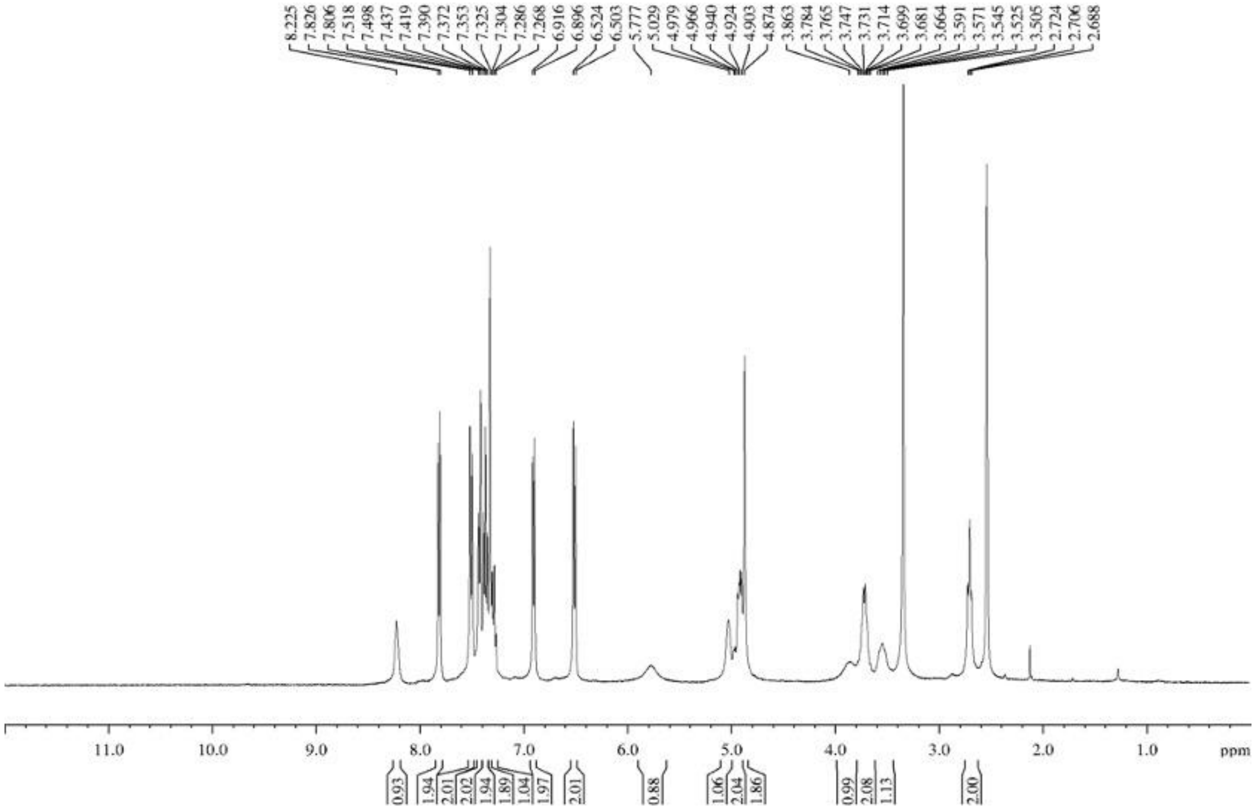

^{1}H NMR spectrum of compound **15** (400 MHz, DMSO-d_6)

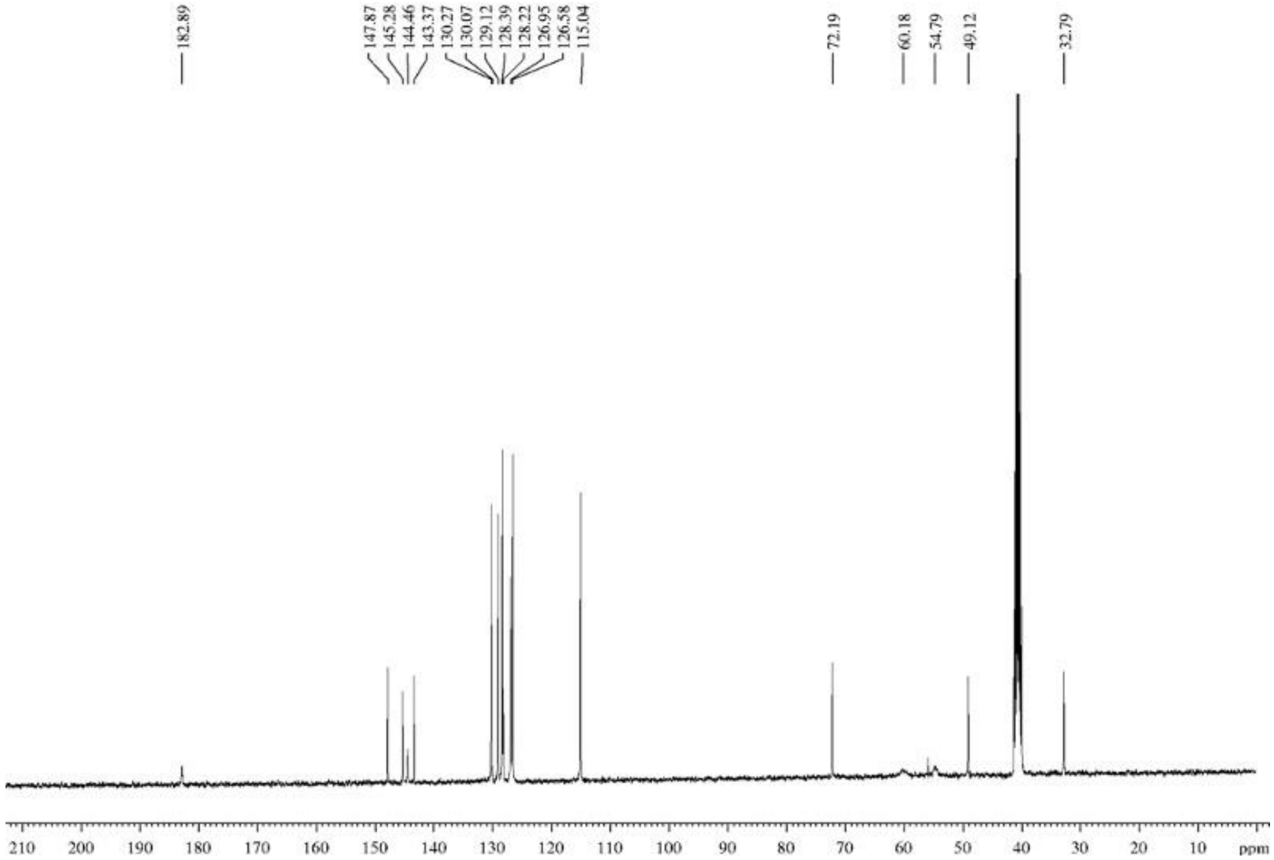

^{13}C NMR spectrum of compound **15** (100 MHz, DMSO-d_6)

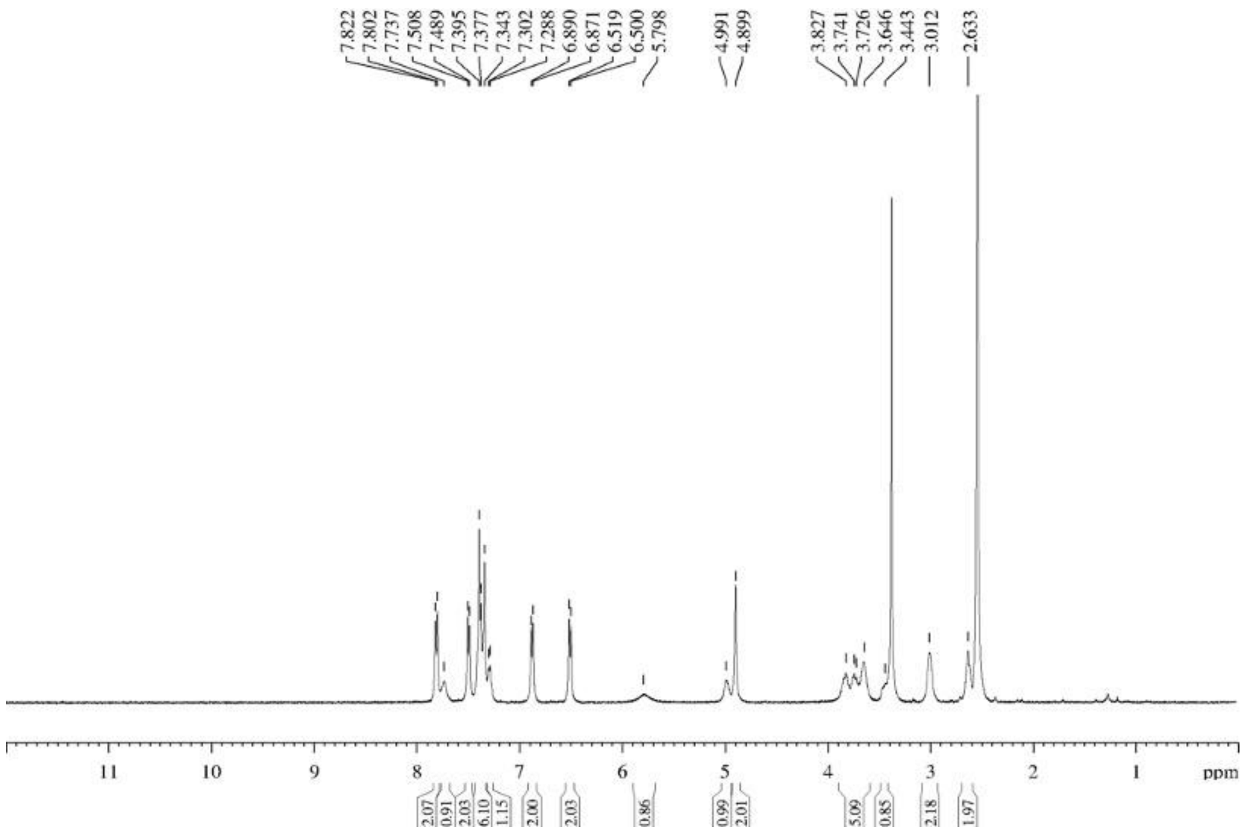

^{1}H NMR spectrum of compound **16** (400 MHz, DMSO-d_6)

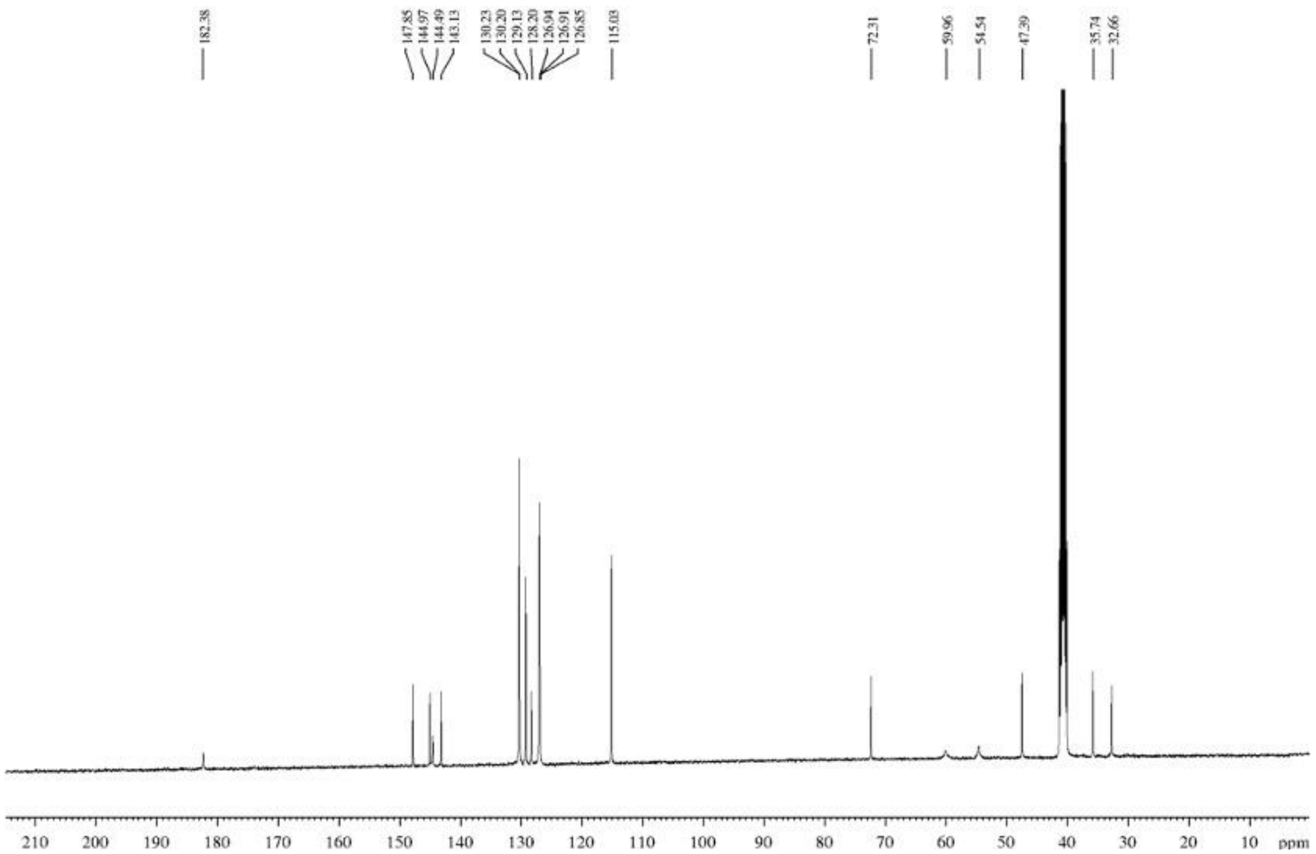

^{13}C NMR spectrum of compound **16** (100 MHz, DMSO-d_6)

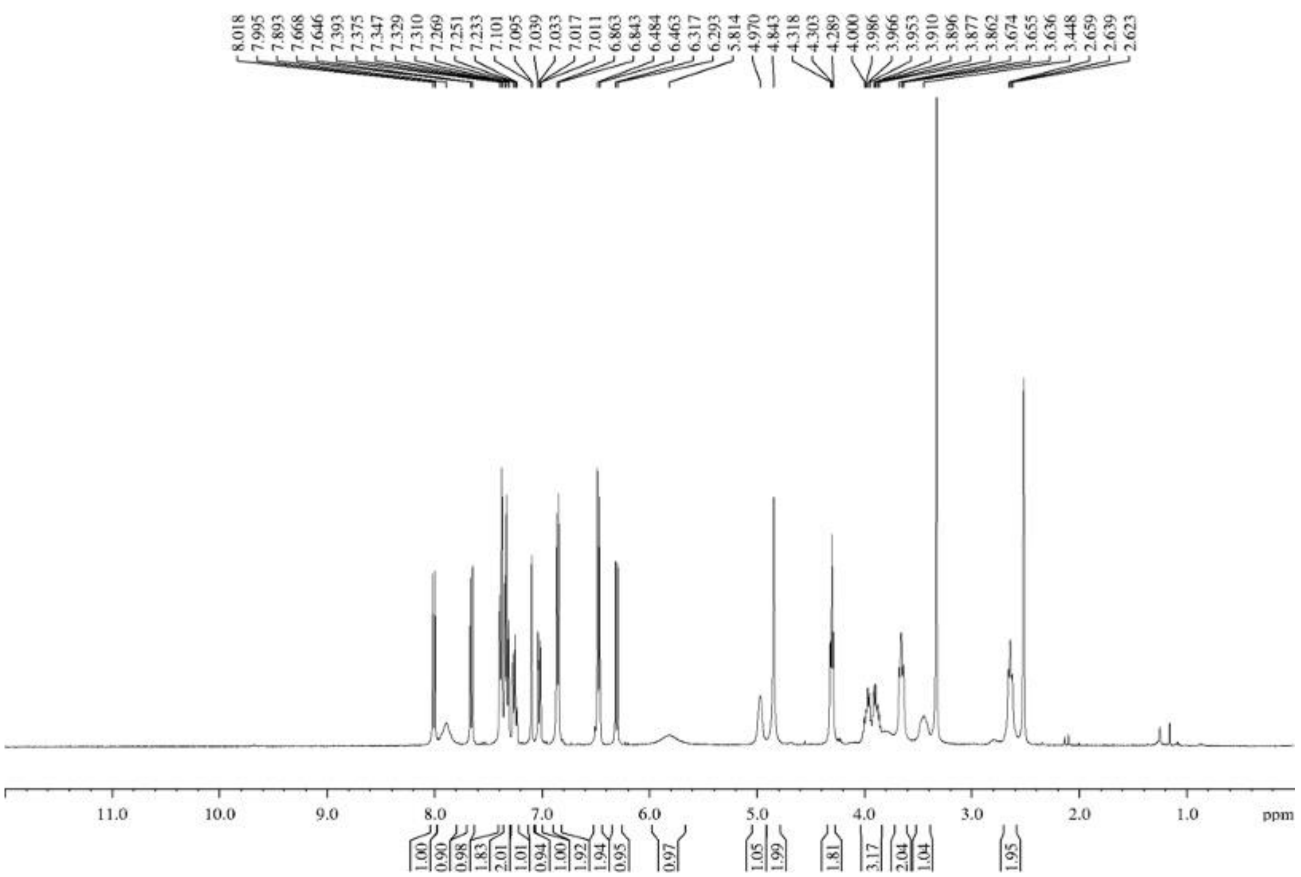

^{1}H NMR spectrum of compound **17** (400 MHz, DMSO-d_6)

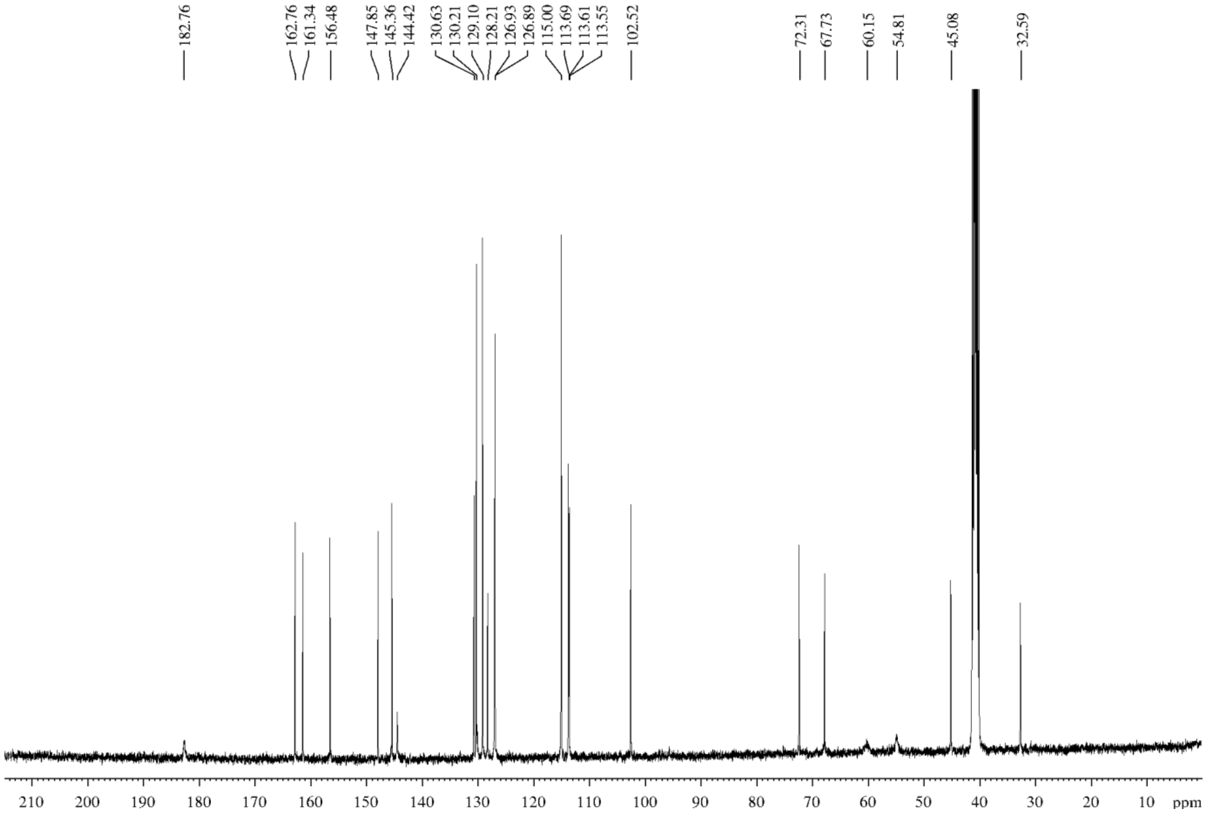

^{13}C NMR spectrum of compound **17** (100 MHz, DMSO-d_6)

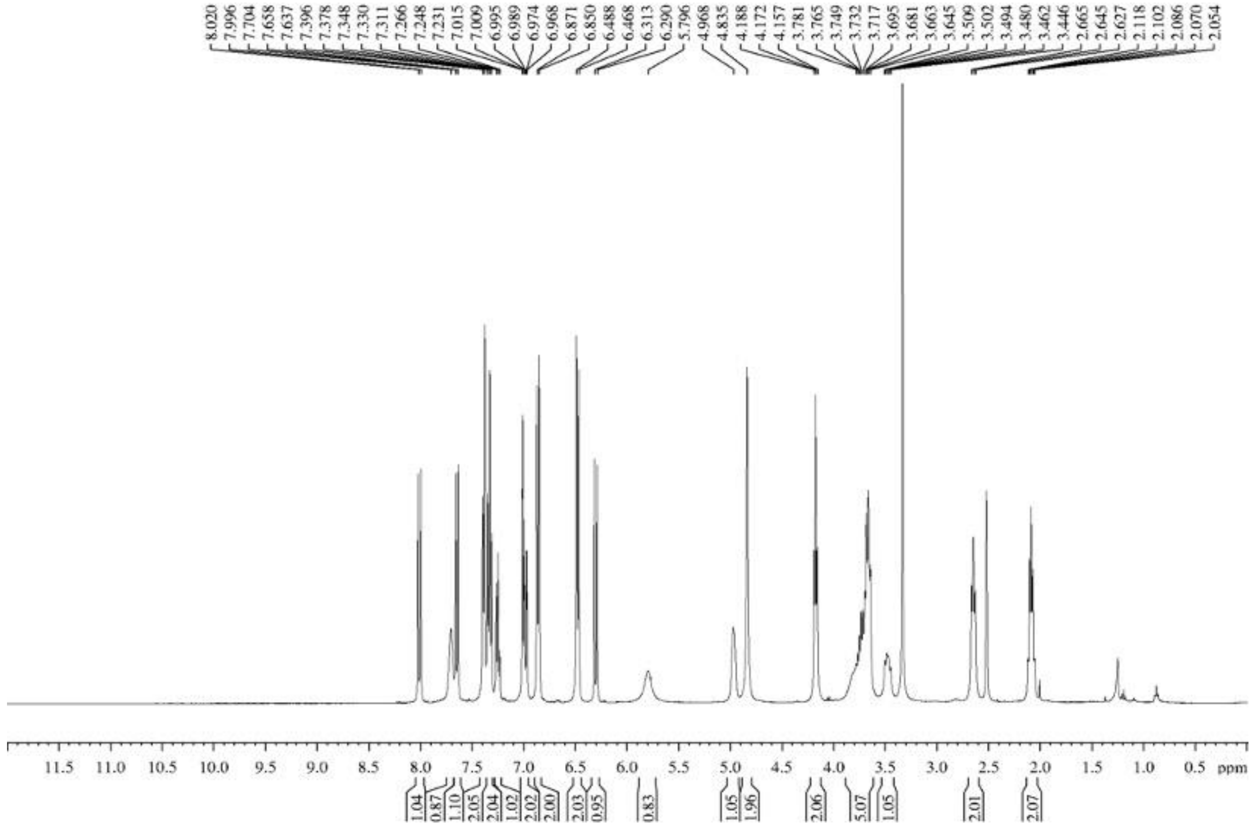

^{1}H NMR spectrum of compound **18** (400 MHz, DMSO-d_6)

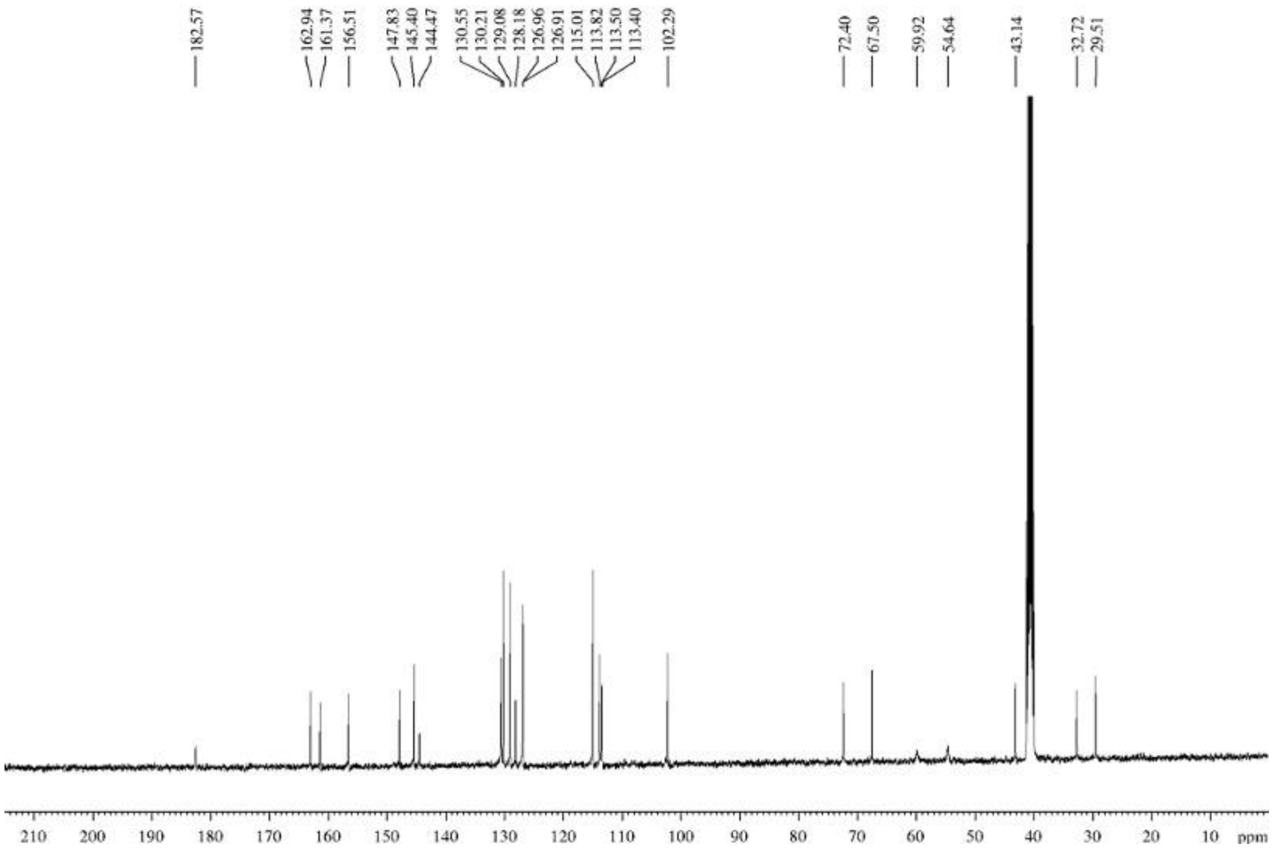

^{13}C NMR spectrum of compound **18** (100 MHz, DMSO-d_6)

Synthesis of aryl and alkyl isothiocyanates **1-9**

A)

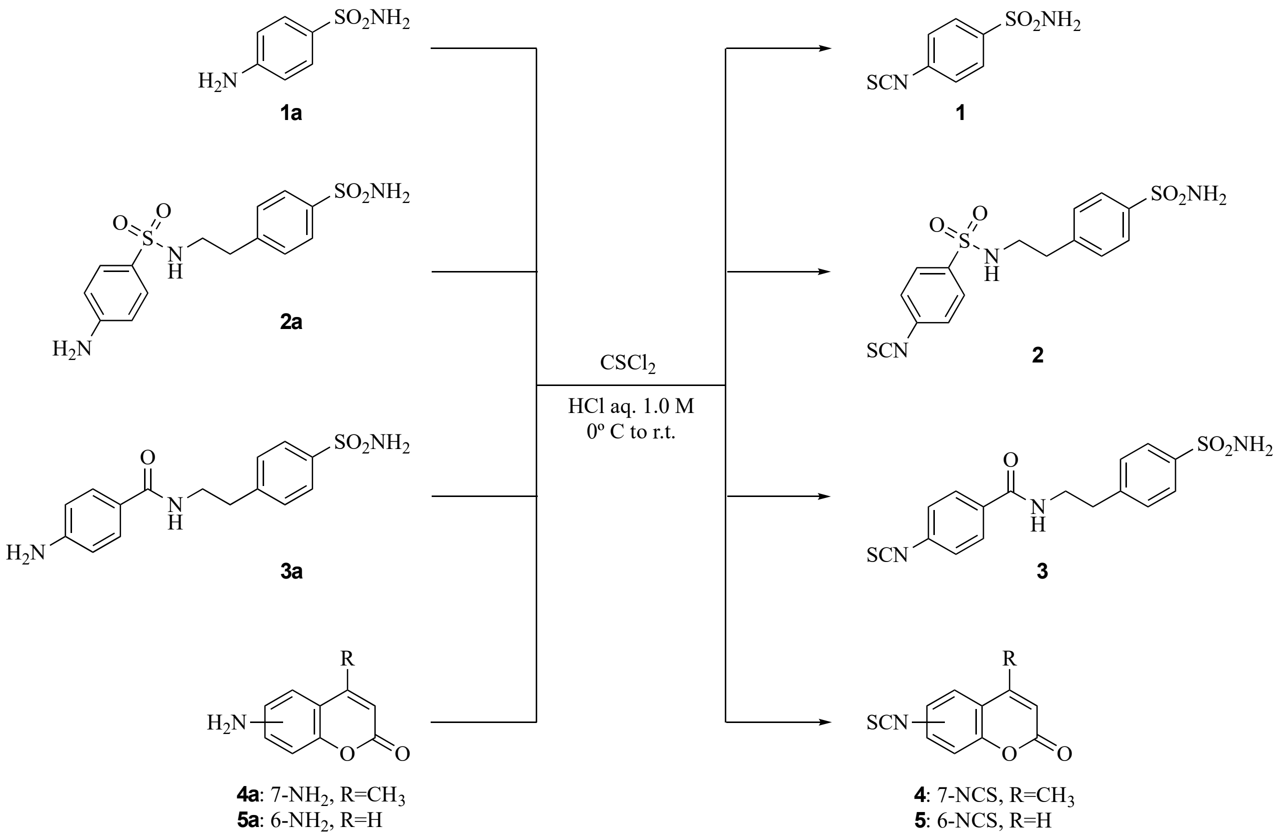

B)

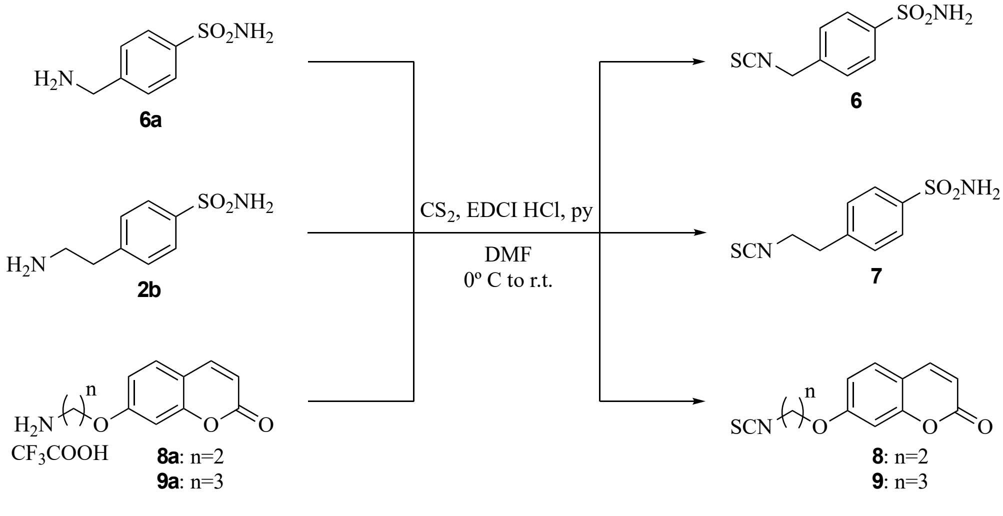

Schemes 1A-B. Synthesis of aryl and alkyl isothiocyanates **1-9**.

General procedure for the synthesis of compounds 1-5.

Thiophosgene (1.25 eq.) was added dropwise at 0 °C to a solution of the appropriate primary aniline **1a**-**5a** (1.0 g, 1.0 eq.) in HCl aq. 1.0 M (15 mL). The reaction mixture was stirred at RT for 3h. The suspension was quenched with slush; the resulting precipitate was filtered and washed with H_2O to give the corresponding isothiocyanates **1-5** which were used without further purification.

4-Isothiocyanatobenzenesulfonamide (1).

Obtained according to the above procedure using 4-isothiocyanatobenzenesulfonamide (**1a**). White solid; yield 94%; **^{1}H NMR** (400 MHz, DMSO-d_6): δ (ppm) 7.88 (d, 2H, J = 8.3 Hz, Ar-H), 7.63 (d, 2H, J = 8.3 Hz, Ar-H), 7.52 (s, 2H, exchange with D_2O, SO_2NH_2).

Experimental data were in agreement with reported data.[1]

4-Isothiocyanato-*N*-(4-sulfamoylphenethyl)benzenesulfonamide (2).

Obtained according to the above procedure using 4-amino-*N*-(4-sulfamoylphenethyl) benzenesulfonamide (**2a**). White solid; yield 83%; **^{1}H NMR** (400 MHz, DMSO-d_6): δ (ppm) 7.85 (t, 1H, J=5.6 Hz, exchange with D_2O, SO_2NH), 7.79 (d, 2H, J=8.5 Hz, 2xAr-H), 7.71 (d, 2H, J=8.2 Hz, 2xAr-H), 7.60 (d, 2H, J=8.5 Hz, 2xAr-H), 7.34 (d, 2H, J=8.2 Hz, 2xAr-H), 7.28 (s, 2H, exchange with D_2O, SO_2NH_2), 3.02 (q, 2H, J=6.8 Hz, CH_2), 2.76 (t, 2H, J=7.4 Hz, CH_2).

Experimental data were in agreement with reported data. [2]

4-Isothiocyanato-*N*-(4-sulfamoylphenethyl)benzamide (3).

Obtained according to the above procedure using 4-amino-*N*-(4-sulfamoylphenethyl)benzamide (**3a**). White solid; yield 89%; m.p.: 248-251° C; silica gel TLC Rf: 0.48 (MeOH/DCM 10% v/v); **^{1}H NMR** (400 MHz, DMSO-d_6): δ (ppm) 8.68 (t, 1H, J=5.2 Hz, exchange with D_2O, CONH), 7.86 (d, 2H, J=8.2 Hz, 2xAr-H), 7.74 (d, 2H, J=8.3 Hz, 2xAr-H), 7.51 (d, 2H, J=8.2 Hz, 2xAr-H), 7.42 (d, 2H,

J=8.3 Hz, 2xAr-*H*), 7.27 (s, 2H, exchange with D_2O, SO_2NH_2), 3.51 (q, 2H, *J*=6.2 Hz, CH_2), 2.92 (t, 2H, *J*=7.1 Hz, CH_2); **^{13}C NMR** (100 MHz, DMSO-d_6): δ (ppm) 166.0, 144.8, 143.1 134.5, 133.5, 130.2, 129.8, 128.6, 127.0, 126.8, 41.6, 35.7; **MS (ESI positive) *m*/*z*:** 362.1 $[M + H]^+$.

7-Isothiocyanato-4-methyl-2*H*-chromen-2-one (4).

Obtained according to the above procedure using 7-amino-4-methyl-2*H*-chromen-2-one (**4a**). Pale yellow solid; yield: 91%; **^{1}H NMR** (400 MHz, DMSO-d_6): δ (ppm) 7.89 (d, 1H, *J*=8.6 Hz, Ar-*H*), 7.59 (d, 1H, *J*=2.1 Hz Ar-*H*), 7.49 (dd, 1H, *J*=8.2 Hz, 2.1 Hz, Ar-*H*), 6.48 (s, 1H, Ar-*H*), 2.47 (s, 3H, CH_3).

Experimental data were in agreement with reported data. [3]

6-Isothiocyanato-2H-chromen-2-one (5).

Obtained according to the above procedure using 6-amino-2H-chromen-2-one (**5a**). Pale yellow solid; yield: 88%; **^{1}H NMR** (400 MHz, DMSO-d_6): δ (ppm) 8.04 (d, 1H, *J*=9.1 Hz, Ar-*H*), 7.86 (d, 1H, *J*=2.4 Hz, Ar-*H*), 7.67 (dd, 1H, *J*=8.8 Hz, 2.4 Hz, Ar-*H*), 7.49 (d, 1H, *J*=9.1 Hz, Ar-*H*), 6.61 (d, 1H, *J*=9.1 Hz, Ar-*H*).

Experimental data were in agreement with reported data. [4]

General procedure for the synthesis of compounds 6-9.

The appropriate alkylamine **2b**, **6a**, **8a** or **9a** (1.0 g, 1.0 eq.) was dissolved in anhydrous dimethylformamide (2 mL), under inert atmosphere. The solution was then cooled at 0 °C and treated with pyridine (3.0 eq.), carbon disulfide (2.0 eq.) and EDCI hydrochloride (2.5 eq.). The reaction mixture was stirred at room temperature overnight and then quenched with slush and HCl 1M, to afford the corresponding isothiocyanates **6-9** which were used without further purification.

4-(Isothiocyanatomethyl)benzenesulfonamide (6).

Obtained according to the above procedure using 4-(aminomethyl)benzenesulfonamide (**6a**). Yellow solid; yield: 80%; **^{1}H NMR** (400 MHz, DMSO-d_6): δ (ppm) 7.80 (d, 2H, J=7.8 Hz, Ar-H), 7.51 (d, 2H, J=7.8 Hz, Ar-H), 7.36 (s, 2H, exchange with D_2O, SO_2NH_2), 5.03 (s, 2H, CH_2).

Experimental data were in agreement with reported data. [5]

4-(2-Isothiocyanatoethyl)benzenesulfonamide (7).

Obtained according to the above procedure using 4-(aminoethyl)benzenesulfonamide (**2b**). White solid; yield: 78 %; **^{1}H NMR** (400 MHz, DMSO-d_6): δ (ppm) 7.82 (d, 2H, J=7.8 Hz, Ar-H), 7.53 (d, 2H, J=7.8 Hz, Ar-H), 7.37 (s, 2H, exchange with D_2O, SO_2NH_2), 4.00 (t, 2H, J=6.5 Hz, CH_2), 3.08 (t, 2H, J=6.5 Hz, CH_2).

Experimental data were in agreement with reported data. [6]

7-(2-Isothiocyanatoethoxy)-2*H*-chromen-2-one (8).

Obtained according to the above procedure using 2-((2-oxo-2*H*-chromen-7-yl)oxy)ethan-1-aminium 2,2,2-trifluoroacetate (**8a**). Off-White solid; yield 94%; m.p.: 183-185° C; silica gel TLC Rf: 0.49 (MeOH/DCM 10% v/v); **^{1}H NMR** (400 MHz, DMSO-d_6): δ (ppm) 8.04 (d, 1H, J=9.5 Hz, Ar-H), 7.70 (d, 1H, J=8.8 Hz, Ar-H), 7.09 (d, 1H, J=2.0 Hz, Ar-H), 7.04 (dd, 1H, J=8.2 Hz, 2.0 Hz Ar-H), 6.35 (d, 1H, J=9.5 Hz, Ar-H), 4.38 (t, 2H, J=5.0 Hz, CH_2), 4.13 (t, 2H, J=5.0 Hz, CH_2); **^{13}C NMR** (100 MHz, DMSO-d_6): δ (ppm) 161.4, 160.6, 155.8, 144.7, 130.1, 129.7, 113.7, 113.3, 113.0, 102.0, 67.0, 45.0; **MS (ESI positive) *m/z*:** 248.0 $[M + H]^+$.

7-(3-Isothiocyanatopropoxy)-2*H*-chromen-2-one (9).

Obtained according to the above procedure using 3-((2-oxo-2*H*-chromen-7-yl)oxy)propan-1-aminium 2,2,2-trifluoroacetate (**9a**). White solid; yield 86%; m.p.: 186-188° C; silica gel TLC Rf: 0.51 (MeOH/DCM 10% v/v); **^{1}H NMR** (400 MHz, DMSO-d_6): δ (ppm) 8.03 (d, 1H, J=9.5 Hz, Ar-

H), 7.69 (d, 1H, *J*=8.8 Hz, Ar-*H*), 7.06 (d, 1H, *J*=2.0 Hz, Ar-*H*), 7.02 (dd, 1H, 8.2 Hz, 2.0 Hz, Ar-*H*), 6.33 (d, 1H, J=9.5 Hz, Ar-*H*), 4.23 (t, 2H, *J*=6.2 Hz, CH_2), 3.89 (t, 2H, *J*=6.2 Hz, CH_2), 2.17 (m, 2H, CH_2); **^{13}C NMR** (100 MHz, DMSO-d_6): δ (ppm) 161.9, 160.7, 155.8, 144.7, 130.0, 128.2, 113.2, 113.1, 113.0, 101.8, 66.0, 42.7, 29.4; **MS (ESI positive) *m/z*:** 262.0 [M + H]$^+$.

Synthesis of **2a, 3a, 8a** and **9a.**

A)

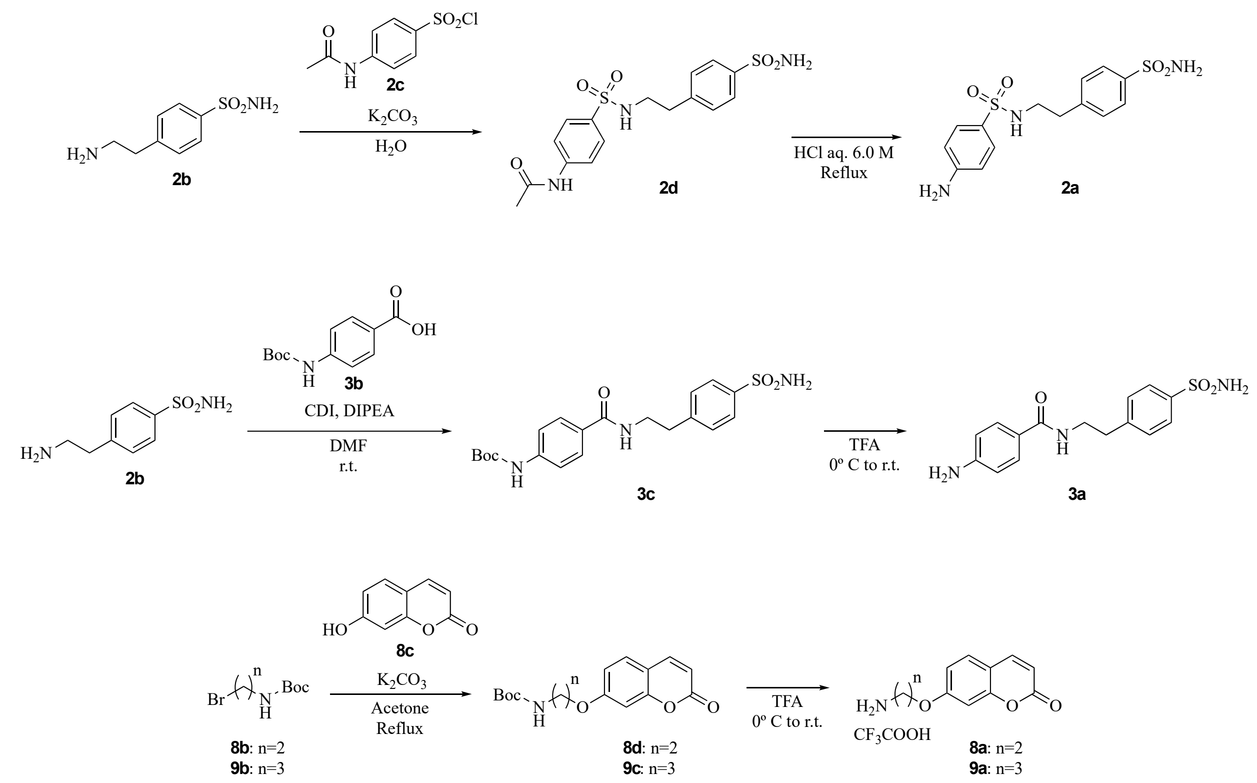

Scheme 2A-C. Synthesis of primary anilines **2a**, **3a** and alkylamines **8a**, **9a**

Synthesis of 4-amino-*N*-(4-sulfamoylphenethyl) benzenesulfonamide (2a).

A stirred suspension of 4-(aminoethyl)benzenesulfonamide **2b (**1.0 g, 1.2 eq.) in water (30 mL) was treated with K_2CO_3 (2.0 eq.) and 4-acetamidobenzenesulfonyl chloride (1.0 eq.). The reaction mixture was stirred at room temperature overnight then quenched with slush and HCl aq. 6.0 M; the resulting precipitate was filtered and washed with water, affording **2d** as a white solid, which was suspended in HCl aq. 6.0 M and refluxed overnight. The reaction was quenched with slush and NaOH aq. 5 M; the readily formed precipitate was collected by filtration, affording **2a** as a white solid, which was used in the following step without further purification. White solid; yield 71%; **^{1}H NMR** (400 MHz, DMSO-d_6): δ (ppm) 7.76 (d, 2H, *J*=7.5 Hz, 2xAr-*H*), 7.45 (d, 2H, *J*=8.6 Hz, 2xAr-*H*), 7.38 (d, 2H, *J*=7.5 Hz, 2xAr-*H*), 7.30 (s, 2H, exchange with D_2O, SO_2NH_2), 7.24 (t, 1H, *J*=5.4 Hz, exchange with D_2O, SO_2NH), 6.64 (d, 2H, *J*=8.6 Hz, 2xAr-*H*), 5.95 (bs, 2H, exchange with D_2O, Ar-NH_2), 2.95 (q, 2H, *J*=6.8 Hz, CH_2), 2.77 (t, 2H, *J*=7.4 Hz, CH_2).

Experimental data were in agreement with reported data.[8]

Synthesis of 4-amino-*N*-(4-sulfamoylphenethyl)benzamide (3a).

Di(1*H*-imidazol-1-yl)methanone was added to a stirred solution of 4-((*tert*-butoxycarbonyl)amino)benzoic acid **3b (**1.0 g, 1.0 eq.) in anhydrous DMF (3 mL), under inert atmosphere. The solution was stirred at room temperature for 30', then treated with 4-(aminoethyl)benzenesulfonamide **2b (**1.2 eq.) and DIPEA (2.5 eq.) and stirred at room temperature overnight. The reaction was quenched with slush and HCl aq. 6.0 M; the readily formed precipitate was filtered and washed with diethyl ether, affording **3c** as a white solid, which was dried and dissolved in ice cold 2,2,2-trifluoroacetic acid (5 ml) and stirred at room temperature for 30'. The solvent was evaporated under reduced pressure, the obtained solid was treated with cold water and NaOH aq. 5 M. The readily formed precipitate was collected by filtration, to afford **3a** as a white solid which was used in the following step without further purification. White solid; yield 78%; **^{1}H NMR** (400 MHz, DMSO-d_6): δ (ppm) 8.13 (t, 1H, *J*=5.1 Hz, exchange with D_2O, CON*H*), 7.78 (d, 2H, *J*=7.2 Hz, 2xAr-*H*), 7.58 (d, 2H, *J*=8.5 Hz, 2xAr-*H*), 7.45 (d, 2H, *J*=7.2 Hz, 2xAr-*H*), 7.31 (s, 2H, exchange with D_2O, SO_2NH_2), 6.57 (d, 2H, *J*=8.5 Hz, 2xAr-*H*), 5.62 (bs, 2H, exchange with D_2O, Ar-NH_2), 3.49 (q, 2H, *J*=6.2 Hz, CH_2), 2.92 (t, 2H, *J*=7.1 Hz, CH_2).

Experimental data were in agreement with reported data.[8]

General procedure C.

A stirred solution of 7-hydroxy-2*H*-chromen-2-one **8c** (2.0 g, 1.0 eq.) in acetone (30 mL) was treated with K_2CO_3 (2.0 eq.) and the appropriate *N*-Boc alkyl bromide **8b**, **9b** (1.2 eq.) and refluxed overnight. The solvent was evaporated under reduced pressure, the obtained solid was treated with slush and HCl aq. 1.0 M, collected by filtration, washed with diethyl ether and dried, to afford the corresponding derivatives **8d** or **9c**. The appropriate derivative was dissolved in ice cold 2,2,2-trifluoroacetic acid (5 mL) and stirred at room temperature for 30'. The solvent was evaporated under reduced pressure

and the resulting solid was triturated with diethyl ether and collected by filtration, affording the corresponding derivatives **8a** or **9a** as trifluoroacetate salts.

2-((2-Oxo-2*H*-chromen-7-yl)oxy)ethan-1-aminium 2,2,2-trifluoroacetate (8a).

Obtained following general procedure **C** using *tert*-butyl (2-bromoethyl)carbamate (**8b**). White solid; yield 65%; **^{1}H NMR** (400 MHz, DMSO-d_6): δ (ppm) 8.06 (d, 1H, J=9.5 Hz, Ar-H), 7.97 (bs, 3H, exchange with D_2O, NH_3^+), 7.72 (d, 1H, J=8.8 Hz, Ar-H), 7.03 (d, 1H, J=2.0 Hz, Ar-H), 7.0 (dd, 1H, J=8.2 Hz, 2.0 Hz, Ar-H), 6.35 (d, 1H, J=9.5 Hz, Ar-H), 4.25 (t, 2H, J=5.0 Hz, CH_2), 3.30 (m, 2H, CH_2).

Experimental data were in agreement with reported data.[9]

3-((2-Oxo-2*H*-chromen-7-yl)oxy)propan-1-aminium 2,2,2-trifluoroacetate (9a).

Obtained following general procedure **C** using *tert*-butyl (3-bromopropyl)carbamate (**9b**). White solid; yield 62%; **^{1}H NMR** (400 MHz, DMSO-d_6): δ (ppm) 8.05 (d, 1H, J=9.5 Hz, Ar-H), 7.78 (bs, 3H, exchange with D_2O, NH_3^+), 7.70 (d, 1H, J=8.8 Hz, Ar-H), 7.05 (d, 1H, J=2.0 Hz, Ar-H), 7.01 (dd, 1H, J=8.2 Hz, 2Hz, Ar-H), 6.35 (d, 1H, J=9.5 Hz, Ar-H), 4.21 (t, 2H, J=6.2 Hz, CH_2), 3.06 (t, 2H, J=6.2 Hz, CH_2), 2.06 (m, 2H, CH_2).

Experimental data were in agreement with reported data.[9]

Table S1. Inhibition percentage of specific binding of compounds **10-18** and **M16** at 1, 10 and 100 μM concentration to cells membranes stably transfected with β_1-, β_2- and β_3-ARs.

	Specific binding (inhibition, %)			Specific binding (inhibition, %)			Specific binding (inhibition, %)		
**Cmp**	β_1-AR**			β_2-AR**			β_3-AR**		
	**1 μM**	**10 μM**	**100 μM**	**1 μM**	**10 μM**	**100 μM**	**1 μM**	**10 μM**	**100 μM**
**10**	4.1 ± 2.2	13.7 ± 3.9	37.2 ± 2.4	10.2 ± 4.1	10.9 ± 2.7	65.2 ± 1.8	8.9 ± 15.3	26.8 ± 6.4	91.5 ± 1.8
**11**	0 ± 4.6	1.1 ± 1.5	27.7 ± 3.8	0.1 ± 7.2	11.9 ± 9.0	30.1 ± 2.7	9.3 ± 4.7	38.0 ± 5.8	78.2 ± 2.7
**12**	0 ± 3.7	0 ± 3.5	41.7 ± 6.3	0 ± 3.6	6.6 ± 6.2	51.7 ± 2.8	9.9 ± 1.7	25.9 ± 3.5	74.1 ± 3.2
**13**	5.4 ± 2.4	14.7 ± 4.9	42.7 ± 4.3	13.2 ± 2.3	21.8 ± 3.5	71.0 ± 3.3	5.8 ± 2.1	32.9 ± 4.6	70.5 ± 1.8
**14**	1.1 ± 2.6	9.8 ± 2.9	27.6 ± 3.1	9.9 ± 3.7	14.7 ± 3.0	62.5 ± 3.2	7.9 ± 1.6	41.5 ± 2.7	84.2 ± 1.3
**15**	6.7 ± 4.8	2.0 ± 2.0	26.6 ± 4.5	2.5 ± 5.4	20.3 ± 6.3	75.7 ± 2.5	10.3 ± 3.2	19.8 ± 3.6	82.9 ± 2.2
**16**	1.8 ± 7.3	6.5 ± 7.9	42.7 ± 7.5	2.4 ± 6.1	11.9 ± 4.6	66.5 ± 1.9	15.6 ± 5.6	21.0 ± 3.5	79.7 ± 2.5
**17**	12.9 ± 5.1	20.0 ± 2.9	68.2 ± 3.5	7.3 ± 4.7	32.4 ± 5.4	85.1 ± 1.4	15.2 ± 4.1	41.3 ± 4.2	80.6 ± 2.1
**18**	3.1 ± 3.2	17.7 ± 3.3	48.0 ± 4.3	5.7 ± 4.6	12.9 ± 6.9	78.6 ± 3.2	19.6 ± 9.5	49.1 ± 2.6	66.4 ± 1.6
**M16**	11.1 ± 8.8	44.3 ± 9.8	93.4 ± 0.9	19.2 ± 3.4	65.3 ± 3.3	100.9±0.7	14.9 ± 4.9	53.9 ± 4.1	98.3 ± 1.5

** Data are reported as inhibition percentage of specific binding towards β_1-, β_2- and β_3-ARs subtypes and are presented as mean ± S.E.M. of three to eight experiments, each one performed in duplicate. Parameters were statistically evaluated with one-way ANOVA.

Figure S1

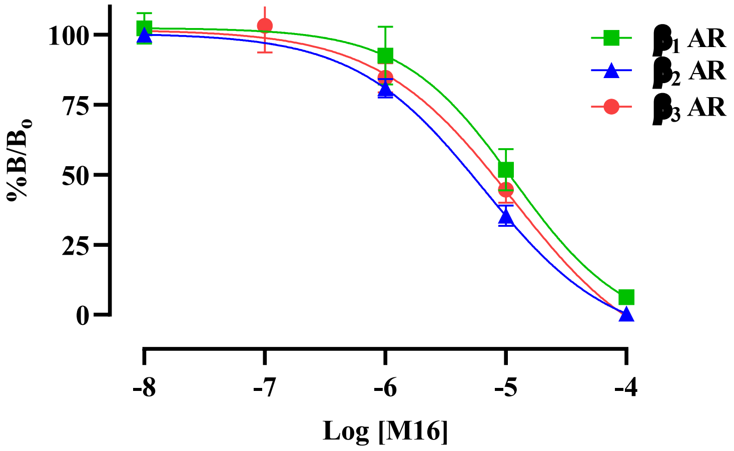

Competition curves showing the effect of increasing concentrations of **M16** on the specific binding of [^{3}H]-CGP12177 (β_1 and β_2) and [^{125}I]-CYP (β_3) to the three subtypes of adrenergic receptors stably expressed in HEK293T cells. Y-axis: normalized Bound/Total bound.

Table S1. MS parameters of compounds **10, 13, 14, 16,** mirabegron and **M16**

	Precursor ion (*m/z*)	Product ion (*m/z*)	DP (V)	EP (V)	CE (V)	CXP (V)
**Mirabegron**	397.2	260.2	89	11	29	19
**M16**	257.2	120	125	11	39	25
**10**	471.2	120	100	11	40	20
		239			25	19
**13**	474.2	120	109	11	40	20
		239			26	19
**14**	460.2	120	96	11	40	20
		239			25	19
**16**	499.2	120	96	11	48	20
		239			26	19

The dwell time was 200 msec for each transition. Q1 and Q3 resolution were set to unit.

Figure S2

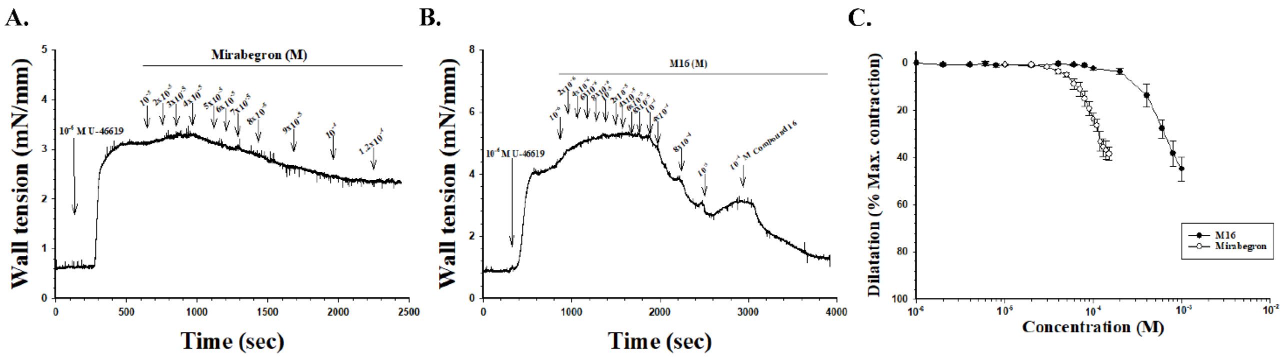

The effects of increasing doses of mirabegron **A**), and **M16 B**) on the wall tension of isolated porcine retinal arterial segments, pre-contracted with the thromboxane A2 analog **U-46619**. The horizontal line above the sample recordings shown in panels A and B indicates the presence of each test compound in the tissue bath. The arrows underneath that line indicate the addition of a new dose of the compound, with the values above the arrows indicating the concentration of the compound in the bath at that point. Panel **C** shows two separate mean concentration-response curves, for each of the compounds. The abscissa expresses the log concentration of the compound, in Molarity. The ordinate presents vasodilation as a percentage of **U-46619** induced maximum contraction of the vessel.

Figure S3

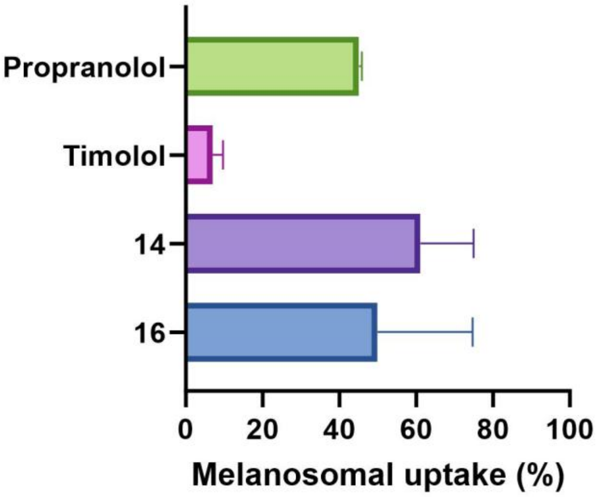

Melanosomal uptake of the coumarin conjugate **14** and benzenesulfonamide conjugate **16** following incubation with porcine ocular melanosomes (0.1 µg/µL) for 2 hours. Propranolol (high-affinity melanin binder) and timolol (intermediate-affinity melanin binder) were included as reference compounds. [10]

References

1) Giovannuzzi, S.; D'Ambrosio, M.; Luceri, C.; Osman, S. M.; Pallecchi, M.; Bartolucci, G.; Nocentini, A.; Supuran, C. T. Aromatic Sulfonamides including a Sulfonic Acid Tail: New Membrane Impermeant Carbonic Anhydrase Inhibitors for Targeting Selectively the Cancer-Associated Isoforms. *Int J Mol Sci*. **2021**, *1*, 461.

2) Cecchi, A.; Ciani, L.; Winum, J-Y.; Montero, J. L.; Scozzafava, A.; Ristori, S.; Supuran, C. T. Carbonic anhydrase inhibitors: design of spin-labeled sulfonamides incorporating TEMPO moieties as probes for cytosolic or transmembrane isozymes. *Bioorg Med Chem Lett*. **2008**, *12*, 3475-80.

3) Voutsadaki, S.; Tsikalas, G. K.; Klontzas, E.; Froudakis, G. E.; Pergantis, S. A.; Demadis, K. D.; Katerinopoulos, H. E. A cyclam-type ‘‘turn on’’ fluorescent sensor selective for mercury ions in aqueous media. *RSC Advances*, **2012**, *2*, 12679–12682.

4) Thacker, P.S.; Srikanth, D.; Angeli, A.; Singh, P.; Chinchilli, K.K.; Arifuddin, M.; Supuran, C.T. Coumarin-Thiourea Hybrids Show Potent Carbonic Anhydrase IX and XIII Inhibitory Action. *Chem Med Chem*. **2021**, *8*, 1252-1256.

5) Casini, A.; Scozzafava, A.; Mincione, F.; Menabuoni, L.; Supuran, C. T. Carbonic anhydrase inhibitors: synthesis of water soluble sulfonamides incorporating a 4-sulfamoylphenylmethylthiourea scaffold, with potent intraocular pressure lowering properties. *J Enzyme Inhib Med Chem*. **2002**, *5*, 333-343.

6) Bozdag, M.; Alafeefy, A. M.; Carta, F.; Ceruso, M.; Al-Tamimi, A. M. S.; Al-Kahtani, A. A.; Alasmary, F. A. S.; Supuran, C. T. Synthesis 4-[2-(2-mercapto-4-oxo-4H-quinazolin-3-yl)-ethyl]-benzenesulfonamides with subnanomolar carbonic anhydrase II and XII inhibitory properties. *Bioorg Med Chem*. **2016**, *18*, 4100-4107.

7) Puccetti, L.; Fasolis, G.; Cecchi, A.; Winum, J-Y.; Gamberi, A.; Montero, J-L.; Scozzafava, A.; Supuran, C. T. Carbonic anhydrase inhibitors: synthesis and inhibition of cytosolic/tumor-

associated carbonic anhydrase isozymes I, II, and IX with sulfonamides incorporating thioureido-sulfanilyl scaffolds. *Bioorg Med Chem Lett*. **2005**, *9*, 2359-2364.

8) Marques, S. M.; Enyedy, E. A.; Supuran, C. T.; Krupenko, N. I.; Krupenko, S. A.; Santos, M. A. Pteridine-sulfonamide conjugates as dual inhibitors of carbonic anhydrases and dihydrofolate reductase with potential antitumor activity. *Bioorg Med Chem*. **2010**, *14*, 5081-5089.

9) Bua, S.; Di Cesare Mannelli, L.; Vullo, D.; Ghelardini, C.; Bartolucci, G.; Scozzafava, A.; Supuran, C. T.; Carta, F. Design and Synthesis of Novel Nonsteroidal Anti-Inflammatory Drugs and Carbonic Anhydrase Inhibitors Hybrids (NSAIDs-CAIs) for the Treatment of Rheumatoid Arthritis. *J Med Chem*. **2017**, *3*, 1159-1170.

10) Hellinen, L.; Hagström, M.; Knuutila, H.; Ruponen, M.; Urtti, A.; Reinisalo, M. Characterization of artificially re-pigmented ARPE-19 retinal pigment epithelial cell model. *Sci Rep*. **2019**, *1*, 13761.
